# Rational design of an epitope-centric vaccine against *Pseudomonas aeruginosa* using pangenomic insights and immunoinformatics approach

**DOI:** 10.3389/fimmu.2025.1617251

**Published:** 2025-09-01

**Authors:** Santhosh Mudipalli Elavarasu, Sasikumar K

**Affiliations:** ^1^ Department of Integrative Biology, School of Biosciences and Technology, Vellore Institute of Technology (VIT), Vellore, Tamil Nadu, India; ^2^ Department of Sensor and Biomedical Technology, School of Electronics Engineering, Vellore Institute of Technology (VIT), Vellore, Tamil Nadu, India

**Keywords:** *Pseudomonas aeruginosa*, pangenome analysis, immunoinformatics, epitope-based vaccine, molecular docking, immune simulation

## Abstract

**Introduction:**

As a highly adaptable opportunistic pathogen, *Pseudomonas aeruginosa* presents a significant threat to people with weakened immune systems. This is because it naturally resists antibiotics and can form biofilms. These factors complicate treatment and underscore the urgent need for innovative therapeutic strategies, such as vaccines, to combat this pathogen.

**Methods:**

A pangenome analysis of *P. aeruginosa* genomes was performed to identify conserved core genes critical for bacterial survival and virulence. LptF, an outer membrane protein, was prioritized as a target for vaccine development. B-cell and T-cell epitopes were predicted from LptF using immunoinformatics tools, and a multi-epitope peptide vaccine was designed. The interaction between the vaccine candidate and Toll-like receptors (TLRs) was investigated through molecular docking and molecular dynamics simulations. Codon optimization and in-silico cloning were carried out to validate the vaccine’s expression potential in *E. coli*. Immune response simulations evaluated the vaccine’s immunogenicity.

**Results:**

Our pangenome analysis identified highly conserved core genes, including LptF, which proved crucial for bacterial virulence. A multi-epitope peptide vaccine was designed using the most immunogenic B-cell and T-cell epitopes derived from LptF. Studies using molecular docking and dynamic simulation have shown stable interactions between the vaccine and TLRs, with the POA_V_RS09 construct exhibiting the highest stability. Codon optimization indicated high expression efficiency in *E. coli*. Immune simulations revealed robust adaptive immune responses, including sustained IgG production, the formation of memory B cells, and the activation of T-cell responses.

**Discussion:**

The POA_V_RS09 vaccine candidate exhibited excellent stability, immunogenic potential, and expression efficiency, making it a promising candidate for combating *P. aeruginosa* infections. This study provides a strong foundation for developing effective therapeutic strategies to address the growing issue of antimicrobial resistance in *P. aeruginosa*. More experimental validation is needed to verify its effectiveness in preclinical and clinical environments.

## Graphical Abstract

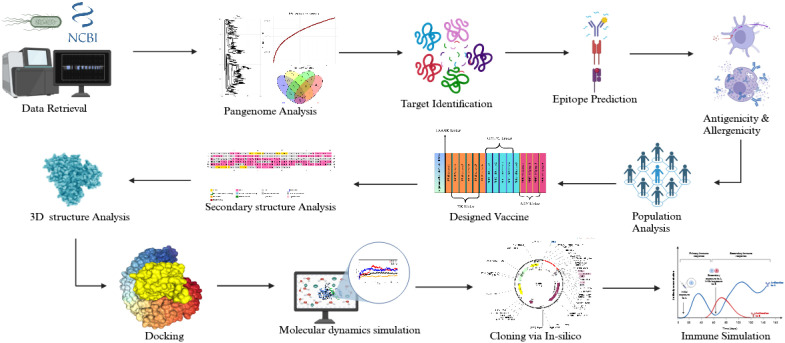

## 1 Introduction

*Pseudomonas aeruginosa* (*P. aeruginosa*), a highly adaptable opportunistic pathogen, is a significant cause of multidrug-resistant (MDR) infections, including diabetic foot infections, ventilator-associated pneumonia, wound infections, septicemia, and catheter-associated urinary tract infections (1). It poses a significant threat, particularly to immunocompromised individuals, due to its intrinsic resistance to antibiotics and its ability to thrive in diverse environments. Furthermore, *P. aeruginosa* can spread through medical equipment, increasing the risk of cross-contamination between patients and complicating infection control in healthcare settings (2). According to the World Health Organization (WHO), antimicrobial resistance (AMR) is expected to cause 10 million deaths annually by 2050, highlighting its severe impact as a global health threat (3). Hospital-acquired infections caused by ESKAPE pathogens, *Enterobacter* species, *P. aeruginosa*, *Staphylococcus aureus, Acinetobacter baumannii, Klebsiella pneumoniae*, and *Enterococcus faecium* are particularly concerning as they employ diverse mechanisms to resist antibiotics, making treatment increasingly challenging (4). Addressing *P. aeruginosa’s* virulence and its role as a key contributor to AMR highlights the urgent need for new therapeutic strategies, such as vaccines, to mitigate its impact (5, 6).

According to the WHO’s 2024 list of critical diseases, *P. aeruginosa* is a high-burden resistant bacterium resistant to last-resort antibiotics (7). Factors contributing to its pathogenicity include secretion systems, biofilm formation, and toxin production. Biofilms protect bacteria from host immune responses and medications, promoting the formation of multidrug-resistant persister cells that cause recurrent infections, particularly in patients with cystic fibrosis (8). *P. aeruginosa* employs its Type III secretion system to inject effector proteins into the host cells, interfering with cellular processes and facilitating immune evasion (9). The bacterium exhibits three primary resistance mechanisms: intrinsic resistance (efflux pumps, antibiotic-inactivating enzymes, limited outer membrane permeability), acquired resistance (mutations or horizontal gene transfer leading to resistance to aminoglycosides, quinolones, and β-lactams), and adaptive resistance (driven by persister cells and biofilm formation) (10). Clinical outcomes of *P. aeruginosa* infections are generally worse than those caused by other bacteria (11–13). During the COVID-19 pandemic, despite a decrease in the overall number of isolates, the percentage of MDR *P. aeruginosa* isolates increased significantly, from 23.8% in 2019 to 38.8% in 2020 (14). This trend was influenced by longer hospital stays, increased ICU admissions, and a greater reliance on empirical antibiotics, primarily due to the severity of cases and the extensive use of mechanical ventilation. This highlights how AMR is exacerbated in healthcare settings during pandemics (15). With the overuse of antibiotics, slow development of new drugs, and increasing complexity of healthcare, AMR is expected to worsen, leading to higher mortality rates and a greater burden on healthcare systems globally. Traditional antibiotics are becoming ineffective against MDR and extensively drug-resistant (XDR) strains, which no longer respond to standard treatments (16). The limited efficacy of last-resort drugs, such as colistin, coupled with their toxicity risks, makes managing resistant infections even more challenging (17). The lack of specific, targeted therapies for resistant infections leaves healthcare providers with limited options, underscoring the need for novel treatments and more effective alternatives to combat AMR (18). Among vaccine development studies for *P. aeruginosa*, outer membrane proteins such as Porin F (OprF) and Lipoprotein I (OprI) have been extensively explored as potential antigen targets (19).

Vaccines are crucial for preventing infections and reducing antibiotic use in low- and middle-income countries, significantly contributing to the fight against AMR. By lowering the incidence of infectious diseases, vaccines help minimize antibiotic misuse and overuse, particularly in populations with limited access to healthcare (20). Vaccines hold significant promise in addressing AMR by preventing infections, reducing antibiotic dependency, and curbing the spread of resistant strains (21). However, designing a vaccine for *P. aeruginosa* has been challenging due to its complex genetic diversity, biofilm formation, and immune evasion capabilities (22). Recent advancements in genomics and immunoinformatics offer new opportunities to overcome these obstacles. Computational tools for identifying novel vaccine candidates pave the way for developing targeted vaccines that can address the diversity of *P. aeruginosa* strains and enhance immune protection (23). In this study, we employed a high-resolution pan-genomic analysis of complete *P. aeruginosa* genomes from the NCBI RefSeq database to identify core, virulence-associated proteins. Among the prioritized candidates, LptF, a component of the LPS transport system, has been classified as a lipotoxin (LPT) due to its ability to trigger strong pro-inflammatory responses via TLR2 activation, particularly in cystic fibrosis. LptF is a pro-inflammatory lipotoxin involved in the excessive induction of IL-8 in cystic fibrosis and remains underexplored as a vaccine target (24). Our pan-genome analysis has identified LptF as a key membrane-associated protein that interacts with virulence factors, such as OprI and LptE, which supports its potential as a new therapeutic candidate (25). Our pipeline integrates reverse vaccinology, structural modeling, and molecular dynamics simulations to design a multi-epitope subunit vaccine construct. Unlike previous studies that relied on reference strains, metabolic enzymes, or limited proteome screening, our approach emphasizes strain-wide conservation, immune accessibility, and functional relevance. This integrative, pathogen-focused design offers a rational and potentially effective strategy for developing a broad-coverage vaccine against MDR *P. aeruginosa*. Using linkers, these epitopes can be linked to effective adjuvants to develop vaccines.

## 2 Materials and methods

### 2.1 Genome data retrieval

A comprehensive dataset of *P. aeruginosa* genomes, all at the “complete” assembly level, was obtained from the National Center for Biotechnology Information (NCBI) database (https://www.ncbi.nlm.nih.gov/) using the NCBI Genome Download Toolkit (26). To ensure comprehensive genomic representation, this dataset included a variety of strains, encompassing both clinical isolates and reference strains.

### 2.2 Pangenome construction and analysis

*P. aeruginosa* strains underwent pangenome analysis using the Roary tool (Version 3.13.0) (27). A diverse set of strains was initially selected to capture extensive genetic variability by collecting whole genomes from the NCBI RefSeq database. These genomes were annotated using Prokka (Version 1.14.6), which converted raw sequences into functional gene and protein data (28). Prokka is used to annotate essential genetic elements such as transfer RNA (tRNA), ribosomal RNA (rRNA), and coding sequences (CDS) for each genome, ensuring consistent annotation across all strains. Roary identifies the core and the accessory genes, revealing the conserved and variable genomic regions among *P. aeruginosa* strains. Core genes from all genomes were extracted from the Roary output for further detailed analysis, providing insights into essential genomic elements and potential targets for vaccine or therapeutic development. This pangenome analysis elucidated the genetic composition of the species and identified potential targets for further therapeutic advancements.

### 2.3 Prediction of subcellular localization

Following the identification of core genes, we employed the PSORTb tool (version 3.0.3) to predict their subcellular localization (29). PSORTb, a robust tool for prokaryotic genome analysis, categorized the core genes based on their predicted cellular locations, including cytoplasmic, periplasmic, and outer membrane regions. This study primarily focused on outer membrane proteins due to their accessibility on the bacterial surface, making them ideal targets for vaccine development. To confirm that the selected outer membrane-associated genes did not show homology with the human proteins, we conducted a comparison against the human proteome using BLASTP analysis (E-value 0.0001) (30). This step was essential to avoid potential cross-reactivity and enhance the specificity of vaccine candidate selection.

### 2.4 Analysis using the virulence factor database

The identified outer membrane proteins were analyzed by comparing them to the Virulence Factor Database (VFDB) using BLASTP [E-value = 0.0001, protein sequences from the VFDB full dataset (set B)] (31). This comparative analysis aimed to determine whether the selected protein candidates possess virulence potential, thereby assessing their suitability as targets for therapeutic or vaccine development. By aligning these outer membrane proteins with known virulence factors, we identified candidates with established roles in pathogenicity, enhancing the selection of proteins with significant implications in host-pathogen interactions. The selected target underwent an additional BLASTP search against the *P. aeruginosa* database for further validation (32). This analysis provided insights into the protein’s potential role, supporting its relevance for subsequent stages of the study.

### 2.5 Immunogenic potential and structural characterization of vaccine candidate

We evaluated the selected sequence’s antigenic potential using the VaxiJen v2.0 (https://www.ddg-pharmfac.net/vaxijen/VaxiJen/VaxiJen.html) server (33) to determine its suitability as an antigenic candidate. The sequence was analyzed with Allertop v2.0 (34) to assess allergenic risk, ensuring it lacked properties that could trigger allergic reactions. We used the ProtParam tool (https://web.expasy.org/protparam/) to determine the physicochemical properties, including molecular weight, instability index, grand average of hydropathicity (GRAVY), and hydrophobicity (35). These analyses provided essential insights into the protein’s suitability for vaccine development by assessing its immunogenicity, safety, and stability.

### 2.6 Signal peptide prediction analysis

SignalP 6.0 (https://services.healthtech.dtu.dk/services/SignalP-6.0/) is a sophisticated bioinformatics tool designed to detect signal peptides in protein sequences and pinpoint their cleavage sites (36). Utilizing protein language models (LMs), it analyzes the N-terminal region of proteins. Based on the predicted pathway and cleavage mechanism, SignalP classifies signal peptides into various types, such as Sec/SPI and Tat/SPI. The tool provides crucial scores, including the S-score for signal peptide probability and the C-score for predicting cleavage sites. This is essential for developing vaccines, as it helps identify secreted or surface-exposed proteins that could serve as potential immunogenic targets.

### 2.7 Prediction of linear B-cell epitopes

For the prediction of linear B-cell epitopes, we utilized BepiPred 2.0, which relies on the Immune Epitope Database (IEDB) Analysis resource (https://www.iedb.org/) (37, 38). This tool analyses amino acid propensity scores and identifies patterns typical of B-cell epitopes, using propensity scales to identify regions likely to consist of these epitopes. Improved accuracy of predictions is achieved by training on known antigen-antibody complexes, with the Random Forest method refining the results. The antigenic potential of the predicted epitopes was further assessed using VaxiJen v2.0 to determine their ability to stimulate an immune response. In this study, it served as an additional screening tool to prioritize epitopes (B and T Cell epitopes) with higher intrinsic antigenic potential before subjecting them to downstream immunoinformatics and structural analyses. Allertop v2.0 assessed allergenicity, ensuring the epitopes would not trigger allergic reactions. Additionally, the toxicity profiles of the selected epitopes were evaluated using the ToxinPred server (39), making sure they had a low risk of allergic reactions was a key step in designing the vaccine.

### 2.8 Prediction of T-cell epitopes (MHC Class I and II)

Epitope prediction for helper (HTL) and cytotoxic (CTL) T lymphocytes was performed using the NetMHCpan 4.1 algorithm provided by the Immune Epitope Database (IEDB) Analysis Resource (40). The focus was on non-structural (NS) proteins, which are conserved across various strains of *P. aeruginosa* and serve as key targets for immune responses. A human-specific approach was employed for CTL epitopes, identifying 10-mer peptides (ten amino acids long) that included 27 common HLA alleles as a reference panel. These epitopes were chosen for their ability to bind to MHC class I molecules and activate cytotoxic T cells, which is essential for targeting and eliminating infected cells. We selected T-cell epitopes based on recommendations from the IEDB for binding predictions. Specifically, we selected epitopes with a percentile rank of ≤ 1% for MHC class I, and a median percentile rank of ≤20% for MHC class II. These thresholds represent high and moderate affinity binders, and we mapped them to our scoring scale (≥ 0.60 for class I and ≥ 0.75 for class II) to include biologically relevant epitopes (41). For HTL epitopes, 15-mer peptides likely to stimulate helper T cells were identified using the IEDB-recommended method. These epitopes were designed to bind to MHC class II molecules, thereby activating B cells and initiating the humoral immune response. The input included antigenic, non-allergenic, and NS proteins from *P. aeruginosa* to ensure the predicted epitope’s efficacy and safety for vaccine development.

### 2.9 Prediction of interferon-γ inducing MHC-II epitopes

In this study, the IFNepitope server was used to predict MHC-II epitopes capable of inducing Interferon-gamma (IFN-γ) responses. This web-based tool leverages a comprehensive dataset from the IEDB, comprising 6,728 non-inducing epitopes and 3,705 confirmed IFN-γ-inducing epitopes (42). Utilizing the Support Vector Machine (SVM) technique, the server combines sequence analysis with predictive algorithms to identify epitopes with a high potential to stimulate IFN-γ production. We also analyzed the IL-4 prediction web server (43), the IL-6 prediction web server (44), the IL-10 prediction web server (45), and the IL-13 prediction web server (46). Epitopes were selected for vaccine development based on their prior assessment for antigenicity and non-allergenicity. This tool also prioritizes safe and immunologically relevant epitopes, which boosts the chances of a successful immune response.

### 2.10 Analysis of population coverage

The finalized T-cell epitopes and their associated HLA binding data were evaluated using the IEDB’s Population Coverage module to determine their global distribution across diverse populations (47). This analysis provided valuable insights into the epitope’s coverage across different demographics and regions, enhancing our understanding of their potential immunological effectiveness. By examining the global distribution of these epitopes, the study highlighted their relevance to diverse demographic groups. This crucial step demonstrated the epitope’s ability to address global healthcare needs, ensuring the vaccine candidate’s potential to protect a wide range of populations, thereby increasing its worldwide applicability and efficacy.

### 2.11 Vaccine design and construction

This study enhanced the vaccine design by incorporating carefully selected adjuvants, linkers, and epitopes to amplify the immune response. Two adjuvants were selected for their immune-boosting properties: RS-09 (APPHALS), a short peptide mimicking bacterial lipopolysaccharide, and Beta-defensin, a potent antimicrobial peptide known for its strong immunological activation (48, 49). Four linkers were used to achieve the best positioning and presentation of the epitopes. The EAAAK linker connected the adjuvants to the epitopes. This rigid helical linker promotes spatial separation between the adjuvant and the epitope region, thereby minimizing potential structural interference and enhancing adjuvant-mediated immune activation. The Alanine-Alanine-Tyrosine (AAY) linker was employed between MHC-I epitopes to enhance processing and presentation by MHC class I molecules. The MHC-II epitopes were separated using the Glycine-Proline-Glycine-Proline-Glycine (GPGPG) linker, which is a flexible and hydrophilic linker that aids in preserving epitope integrity and enhances recognition by helper T cells. Finally, the KK (Lysine-Lysine) linker was used to connect B-cell epitopes, ensuring adequate exposure for B-cell activation while maintaining their conformational flexibility and immunogenicity (50). These strategic additions of adjuvants and linkers were designed to optimize the vaccine’s ability to elicit strong and targeted immune responses, effectively combating the intended disease.

### 2.12 Analysis of the physicochemical properties of the formulated vaccines

The ProtParam server was utilized to conduct a physicochemical analysis of the developed vaccine candidates, assessing their stability and suitability for development (51). We analyzed the amino acid sequences to identify key structural and functional features. We calculated the molecular weight to estimate the proteins’ size, solubility, and potential antigenicity. To assess their biochemical behavior under physiological conditions, we determined the theoretical isoelectric point (pI), which indicated their net charge and acid–base characteristics. We also computed the instability index to predict the likelihood of protein degradation. However, the aliphatic index was evaluated to determine temperature stability based on the contribution of aliphatic amino acids. The GRAVY index was also evaluated to determine the vaccine’s overall hydrophobic or hydrophilic nature, aiding in understanding its solubility and stability.

### 2.13 Secondary structure analysis and prediction

The secondary structure of the developed vaccine was predicted using the PSIPRED tool (52), a widely used online resource for protein structure annotation and prediction. PSIPRED offers comprehensive protein analysis tools (53), with a focus on structural feature prediction. This analysis yielded valuable insights into how the vaccine might interact, its stability, and its functional properties. After entering the amino acid sequence of the final vaccine construct, the PSIPRED server analyzed the sequence and predicted the secondary structure, identifying coil, β-sheet, and α-helical regions. These predictions provide crucial insights about the overall structure and organization of the vaccine’s protein backbone.

### 2.14 Prediction and computational refinement of tertiary structure

To predict the three-dimensional (3D) structure of the developed vaccine and facilitate docking analysis, the ROBETTA server and AlphaFold (54, 55), which employ deep-learning techniques for accurate protein modeling, were utilized. The complete amino acid sequence of the vaccine was entered into both platforms, resulting in the prediction of multiple 3D structures in PDB format. These structures were enhanced in quality and accuracy using the GalaxyRefine tool (56). This tool refines the models by correcting structural errors, optimizing energy levels, and minimizing steric clashes. A comparative analysis of the refined models was conducted, and the best-performing structure, as determined by structural validation using a Ramachandran plot and other quality metrics, was selected for further docking studies.

### 2.15 Molecular docking and interaction studies

We employed molecular docking analysis to examine the interactions between the vaccine construct and Toll-like receptors TLR2 and TLR4, which are critical mediators of innate immune responses to infection. TLR2 was selected for its ability to detect various pathogen-associated molecular patterns and initiate immune responses (57). RS09 is a synthetic TLR4 agonist peptide that stimulates innate immunity. The TLR4 receptor recognizes a TLR4 agonist linked to the N-terminus of the vaccine construct. When TLR4 is activated, it triggers an intracellular signaling process via the NF-kB pathway, resulting in the production of inflammatory cytokines (58, 59). We retrieved the 3D structures of TLR2 and TLR4 from the RCSB PDB database, using IDs 2Z7X and 3FXI for TLR2 and TLR4, respectively, for further analysis (60, 61). Before docking, we thoroughly prepared the receptor structures by removing heteroatoms, water molecules, and bound ligands to ensure accurate analysis. This step was vital to prevent any interference during the docking process. Docking simulations were performed using the HDOCK web server (http://hdock.phys.hust.edu.cn/) (62). It employs a hybrid docking algorithm that combines template-based and free docking methods. In this study, we did blind docking to allow unbiased prediction of potential interaction sites between the vaccine construct and immune receptors. HDOCK, which is known for its intuitive interface and robust protein-protein docking capabilities, facilitated the simulation process by leveraging the refined 3D structure of the vaccine and the immune receptor models of TLR2 and TLR4. The docking affinity scores, indicating the strength of interaction between the vaccine and the receptors, were used to evaluate the results. Additionally, key residues involved in binding interactions were identified, providing insights into how these immune receptors recognize the vaccine. This study helps elucidate how the vaccine may effectively interact with TLR2 and TLR4, key components of the innate immune system, to trigger an immune response.

### 2.16 Molecular dynamics simulation analysis

To conduct molecular dynamics (MD) simulations for 1000ns, we utilized the CHARMM-GUI server’s Solution Builder protocol, applying the CHARMM36 force field to generate the necessary input files (63). The TIP3P water model was used to solvate the protein-protein complexes, creating a realistic simulation environment by enclosing the system in a periodic cubic box extending 10 Å from the protein atoms in all directions (64). Counter ions were added to neutralize the system, ensuring overall charge balance. The Verlet cutoff method was employed with a 10 Å cutoff distance, striking a balance between computational efficiency and accuracy to calculate electrostatic and van der Waals interactions. Bond constraints were applied using the LINCS algorithm to stabilize the simulation by maintaining fixed bond lengths. The Particle Mesh Ewald (PME) method was used to precisely calculate long-range electrostatic interactions, enhancing simulation accuracy in systems with periodic boundary conditions (65). To remove undesirable interactions and stabilize the system, the solvated system was subjected to energy minimization using the steepest descent technique (66). Two equilibration phases followed: the first in the NVT ensemble (constant Number of particles, Volume, and Temperature) to stabilize temperature, and the second in the NPT ensemble (constant number of particles, Pressure, and Temperature) to stabilize pressure. Proper thermostat and barostat techniques maintained constant temperature and pressure levels. This dual equilibration ensured system stability before the production run. The simulation recorded coordinates every 1 ps with a time step of 2 fs, striking a balance between computational efficiency and accuracy. CHARMM-GUI provided Python scripts to convert topology (top) and parameter (itp) files into GROMACS-compatible formats, simplifying input file preparation (67). Following the post-production run, we performed thorough trajectory analyses, including calculating Root Mean Square Deviation (RMSD) for structural stability, Root Mean Square Fluctuation (RMSF) for flexibility, hydrogen bond analysis (HBOND) for molecular interactions, Principal Component Analysis (PCA) for dominant motion patterns, Buried Surface Area (BSA) for evaluating binding stability, and Free Energy Landscape (FEL) analysis for the conformational states of the protein-protein complexes. Free energy calculations were performed for the interaction between TLR complexes and the vaccine construct (POA_V_RS09, POA_V_BDEF) using the MM-PBSA method with a Poisson-Boltzmann approach (68, 69). These approaches account for various energy components, including bonded interactions, van der Waals forces, electrostatic effects, and both polar and non-polar solvation energies. Here in MM-PBSA, the polar solvation energy is derived from the Poisson–Boltzmann equation, utilizing the molecular dynamics (MD) trajectory to compute interaction energies throughout the simulation. These analyses provided valuable insights into structural stability, flexibility, interaction dynamics, and potential conformational changes, enhancing our understanding of protein-protein interactions over time (70–72).

### 2.17 *In silico* cloning and expression analysis

To ensure optimal expression in the desired host, the gene of interest was first subjected to codon optimization using the GenScript program (www.genscript.com), aligning the gene sequence with the host’s preferred codon usage (73). Using SnapGene software (https://www.snapgene.com/), we cloned the vaccine construct via in silico. The result showed that the gene of interest and the pET-28a(+) plasmid did not share any restriction sites. This was addressed by adding specific nucleotide sequences to the gene’s N-terminal and C-terminal ends, which matched the restriction sites XhoI and NdeI, thereby aiding in cloning. These sequences provided suitable restriction sites for the accurate insertion of the gene into the plasmid. The appropriate recombinant plasmid construct was then produced by cloning the codon-optimized gene into the pET-28a(+) plasmid in silico using additional restriction sequences (74).

### 2.18 C-IMMSIM-based immune simulation

The C-IMMSIM server (https://kraken.iac.rm.cnr.it/C-IMMSIM/index.php) (75), a widely used tool for simulating immune responses, was employed to evaluate the in-silico immunological response of the developed vaccine. This server utilizes a simulation-based framework to replicate the function of the human immune system and its organs, with a particular emphasis on primary lymphoid tissues. It uses a position-specific scoring matrix, enhanced by machine learning algorithms, to predict immune reactions. To achieve a total simulation period of 1050 steps, the vaccine and adjuvant were given in three doses: an initial dose, a second dose administered 84 days later, and a third dose administered 1050 days later, spaced eight hours apart. The adjuvant concentration was set to 100, and the injected antigen amount was 1000, following the server’s default parameters. This setup enabled a comprehensive evaluation of the immune response triggered by the vaccine or the adjuvant.

## 3 Results

### 3.1 NCBI data retrieval

A diverse array of 864 complete *P. aeruginosa* genomes, encompassing strains such as PAO1, PA14, PAK, LESB58, and CF39S, was sourced from the NCBI Assembly database utilizing the NCBI-genome-download toolkit. **Supplementary Table S1** contains detailed information on all included genomes, ensuring a comprehensive genomic representation for subsequent analyses.

### 3.2 Pangenome analysis

**Figure 1:**
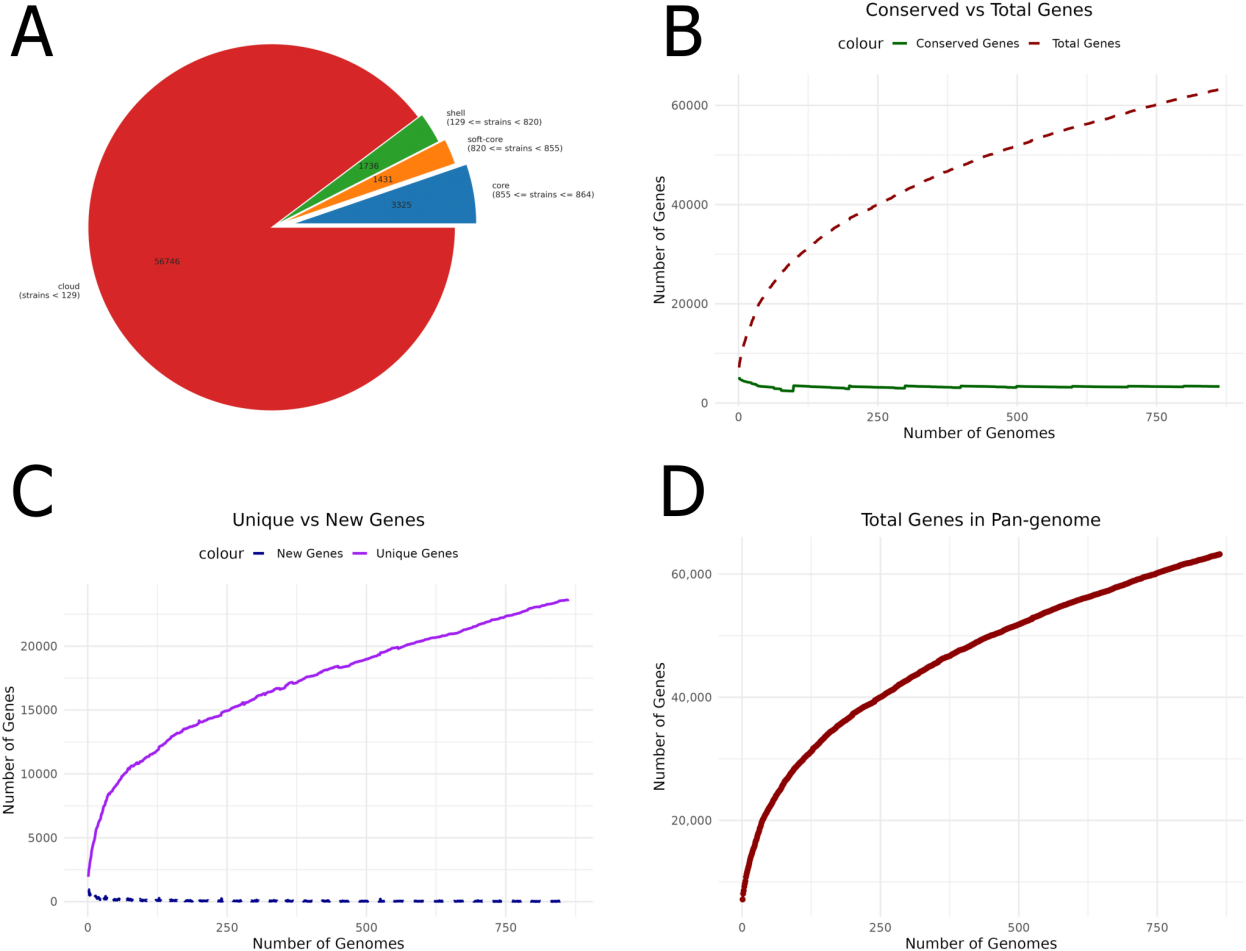
Pangenome analysis of *P. aeruginosa* genomes. **(A)** Pie chart showing the distribution of core, accessory, and unique genes. **(B)** Comparison of conserved genes with the total number of genes, highlighting genetic conservation across genomes. **(C)** Unique versus new gene ratio, emphasizing genome variability. **(D)** The number of genes identified within the pangenome provides insights into overall genomic diversity and its potential impact on vaccine development.

A thorough pangenome analysis was performed on 864 complete genomes of *P. aeruginosa* sourced from the NCBI Assembly database. Genome annotation was executed using Prokka, followed by pangenome analysis with Roary, which identified a total of 63,239 genes. Of these, 3,325 were classified as core genes. Within this core set, 296 genes were consistently present across all genomes, with 79 hypothetical genes excluded from further analysis. Additionally, 3,149 accessory genes were identified in 15–95% of the genomes. The significant genomic diversity revealed by this open pangenome analysis highlights the extensive variability within *P. aeruginosa* (**Figure 1**). The figures were generated using R. This variability provides crucial insights into strain-specific adaptations, pathogenicity, and antibiotic resistance. Furthermore, identifying universally conserved targets among the core genes points to promising candidates for vaccine development applicable across diverse *P. aeruginosa* strains. These findings are pivotal in guiding future research and therapeutic strategies.

### 3.3 Subcellular localization and virulence prediction

PSORTb analysis identified three outer membrane proteins, while the remaining proteins were classified as cytoplasmic or belonging to other categories (**Table 1**). Subsequent BLASTP analysis against the human proteome in NCBI showed no homologous hits for the outer membrane proteins, ensuring their specificity and minimizing the risk of cross-reactivity in vaccine development. BLASTP analysis against the VFDB revealed that only the PAL_1 protein matched known virulence factors, confirming its potential as a relevant target for further therapeutic or vaccine development. Further analysis of PAL_1 against the *P. aeruginosa* database identified the protein as LptF, with an e-value of 0. To validate the conservation of the selected vaccine target LptF across diverse *P. aeruginosa* strains, a multiple sequence alignment was performed using LptF sequences from 864 genomes using Python (76). The conservation analysis revealed that over 98% of the amino acid positions were fully conserved (with 100% identity), and a pairwise sequence identity of greater than 99% was observed among all strains. A corresponding heatmap of the pairwise identity matrix further confirmed the uniform conservation pattern (**Supplementary Figure S1**). These results underscore the evolutionary stability of LptF and support its candidacy as a universal target for vaccine or therapeutic development.

**Table 1:** Subcellular localization predictions for selected proteins based on PsortB analysis.

Sno	Protein	PSORTb result
1	oprB	OuterMembrane – 10.00
2	bamB	OuterMembrane – 10.00
3	pal_1	OuterMembrane – 10.00

### 3.4 Analysis of immunogenic and physicochemical characteristics

The ProtParam tool was used to predict the physicochemical characteristics of the LptF protein. It has a molecular weight of 28.5 kDa and displays slight instability under standard laboratory conditions, with an instability index of 42.30. The GRAVY index of -0.574 indicates its hydrophilic nature. With an aliphatic index of 80.15, which reflects the protein’s thermostability, LptF is considered a strong candidate for vaccine development due to its stability at physiological temperatures. Its potential as an immunogenic candidate is further supported by an antigenicity score of 0.6442 (classified as likely antigenic with a threshold of 0.4) and its classification as non-allergenic by AllerTOP.

### 3.5 Signal peptide prediction

**Figure 2:**
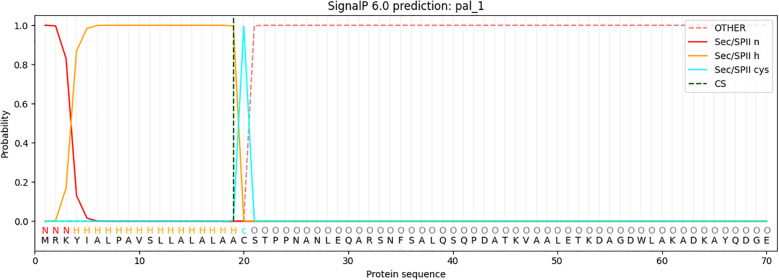
Signal peptide prediction for the LptF protein sequence was performed using SignalP.

The analysis identified a Sec/SPII cleavage site at position 20 of the protein sequence, indicating the presence of a signal peptide that is likely cleaved during the maturation process via the Sec-dependent secretion pathway or the Sec/SPII system (**Figure 2**). With the signal peptide removed, the mature protein sequence begins at position 20. The signal peptide was excluded from further analysis, and the mature protein sequence was used in subsequent bioinformatics analyses. This sequence underwent secondary structure prediction, functional annotation, and potential epitope mapping, all of which are essential for understanding the protein’s biological function and its potential use in vaccine design. This approach ensures that only the biologically relevant mature protein is considered for downstream analyses.

### 3.6 Prediction of B-cell epitope

**Figure 3:**
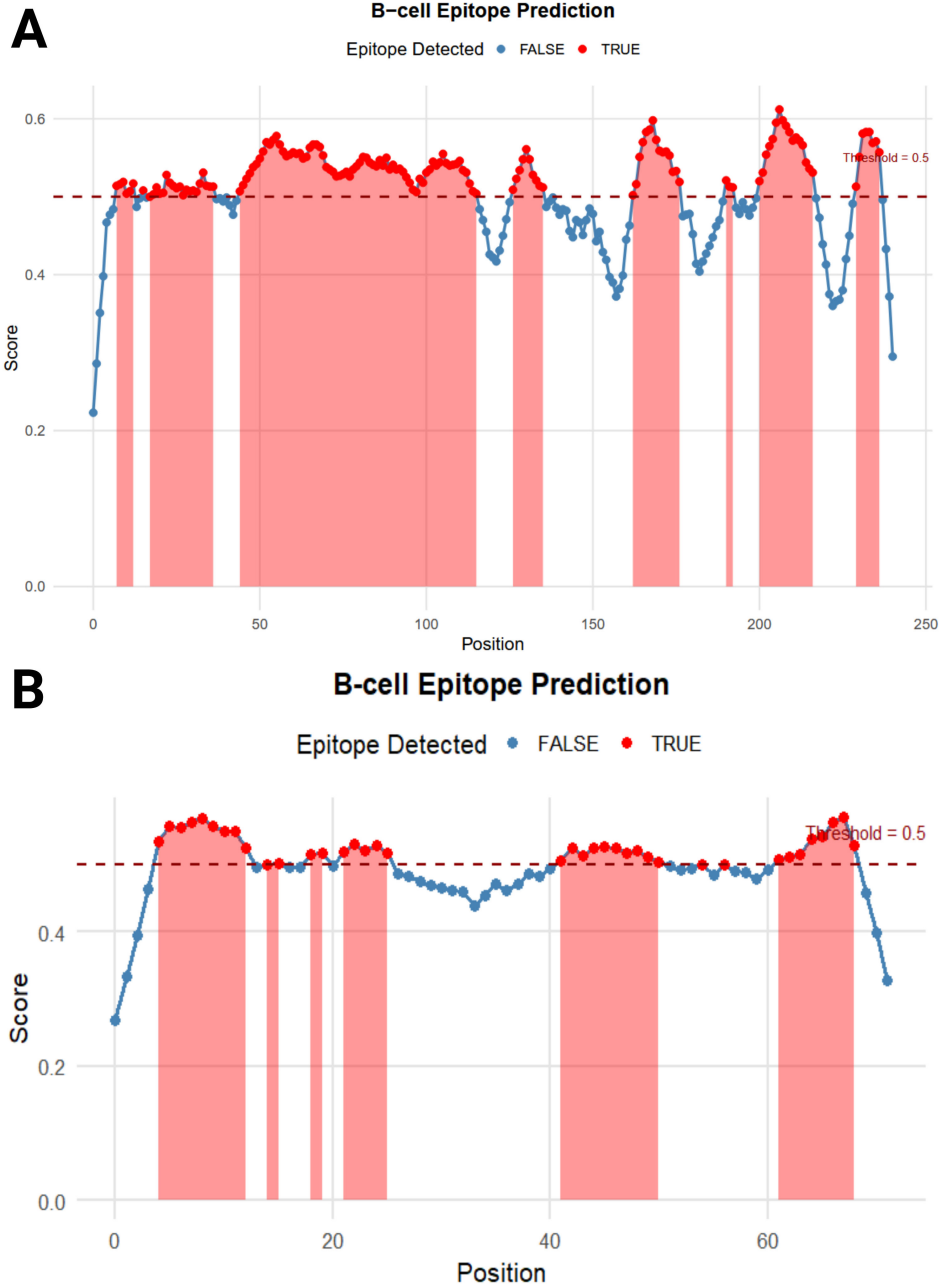
B-cell Epitope Prediction. **(A)** depicts the prediction of epitopes from the entire protein sequence, while **(B)** highlights the re-analyzed 72-mer epitope to ensure no potential epitope is overlooked.

The BepiPred Linear Epitope Prediction 2.0 tool was initially used to predict B-cell epitopes, identifying nine epitopes for the LptF protein. One of these epitopes, a 72-mer, was re-analyzed to ensure no potential epitopes were missed. This re-evaluation revealed eight additional epitopes (**Figure 3**), with figures generated in R (77). They were carefully selected based on several critical factors to confirm the suitability of the identified epitopes for vaccine development. VaxiJen v2.0 predicted high antigenicity scores for these epitopes, indicating their potential to trigger a robust immune response. Additionally, their non-toxic nature was confirmed using ToxinPred, ensuring they would not cause adverse effects. The non-allergenic properties of the epitopes were verified using AllerTOP v2.0, further ensuring their safety. As shown in **Table 2**, the selected epitopes were chosen for further research after careful consideration of these factors. **Supplementary Table S2** provides a detailed analysis of the epitope’s suitability for inclusion in potential vaccine formulations, including their toxicity, allergenicity, and antigenicity profiles.

**Table 2:** Predicted B-cell epitopes for the LptF protein, identified as potential targets for vaccine development.

No	Start	End	Peptide	Length	Antigenicity Score	Probable Antigen	Allergenicity	Toxicity
8	201	217	YGKEYPVASNGTSSGRA	17	1.3767	Antigen	Non-allergen	Non-toxic
5	127	136	DLDKSDLKPG	10	1.1824	Antigen	Non-allergen	Non-toxic
3	18	37	LQSQPDATKVAALETKDAGD	20	0.7914	Antigen	Non-allergen	Non-toxic
1	5	13	GEDQRDVDQ	9	1.4255	Antigen	Non-allergen	Non-toxic
5	42	51	SAQRAQARLD	10	1.2283	Antigen	Non-allergen	Non-toxic
8	62	69	SQLNAKQT	8	1.4671	Antigen	Non-allergen	Non-toxic

### 3.7 Prediction of T-cell epitope (MHC-I and MHC-II)

The MHC-I and MHC-II epitopes were predicted for the LptF protein sequence using NetMHCpan 4.1 from IEDB. The finalized epitopes are presented in **Tables 3**, **4**, with detailed T-cell epitope analyses in **Supplementary Tables S3**, **S4**. While VaxiJen v2.0 is primarily designed for complete proteins, it was utilized here as an additional tool to assess the antigenicity of both MHC class I and II T cell epitopes, supporting selection alongside MHC binding, immunogenicity, and toxicity criteria. Initially, 6,265 MHC-I epitopes were predicted and filtered based on a rank cutoff of 0.5 and a core score cutoff of 0.60. Similarly, 6,130 MHC-II epitopes were filtered using a rank cutoff of 2 and a score of 0.75. These thresholds were chosen because lower rank and score values indicate a higher binding affinity to MHC alleles, which is crucial for identifying effective immunogenic candidates. The finalized epitopes were further assessed for toxicity, antigenicity, and allergenicity to confirm their immunogenic potential while minimizing the risk of adverse reactions. All selected epitopes were predicted to be IL-10 inducers, suggesting their potential to regulate immune responses and prevent excessive inflammation. Notably, epitope 3 exhibited balanced induction of IL-4, IL-6, IL-10, and IL-13, making it a strong vaccine candidate. Epitopes 2 and 4 also induced IL-6 alongside IL-10, supporting a mixed pro-inflammatory and regulatory profile (**Supplementary Table S5**). Interferon-γ scores were computed for MHC-II epitopes to rank those that could elicit a strong immunological response. The chosen MHC-I and MHC-II epitopes, identified according to these criteria, are presented in **Table 4**.

**Table 3:** Finalized MHC-I epitopes identified for the LptF protein.

Allele	Length	Peptide	Score	Rank	Antigenicity Score	Antigen	Allergenicity	Toxicity
HLA-A*01:01	10	YTDSTGSANY	0.9955	0.01	1.3013	Antigen	Non-Allergen	Non-Toxin
HLA-B*57:01	10	QTSRGTMVTF	0.7892	0.22	0.5176	Antigen	Non-Allergen	Non-Toxin
HLA-B*40:01	10	GEDQRDVDQL	0.6866	0.16	1.0027	Antigen	Non-Allergen	Non-Toxin
HLA-A*31:01	10	KSDLKPGAMR	0.6452	0.19	0.9618	Antigen	Non-Allergen	Non-Toxin

**Table 4:** Finalized MHC-II epitopes identified for the LptF protein, optimized for vaccine design.

Allele	Peptide	Score	Rank	Antigenicity Score	Antigen	Allergenicity	IFN-γ Score
HLA-DRB1*03:01	VEVTISNDAKPVAPR	0.9766	0.05	0.4458	Antigen	Non-Allergen	0.0856
HLA-DQA1*01:02/DQB1*06:02	VLRNAEAQLQNASAQ	0.8975	0.01	0.7313	Antigen	Non-Allergen	0.5321
HLA-DRB1*01:01	EAQLQNASAQRAQAR	0.8624	0.61	1.3141	Antigen	Non-Allergen	0.7388
HLA-DQA1*01:02/DQB1*06:02	IVLRNAEAQLQNASA	0.8601	0.03	0.7121	Antigen	Non-Allergen	0.2149
HLA-DQA1*05:01/DQB1*03:01	EAQLQNASAQRAQAR	0.8171	0.31	1.3141	Antigen	Non-Allergen	0.7388
HLA-DQA1*01:02/DQB1*06:02	TIVLRNAEAQLQNAS	0.7961	0.10	0.5276	Antigen	Non-Allergen	0.2391
HLA-DQA1*01:02/DQB1*06:02	EAQLQNASAQRAQAR	0.7768	0.13	1.3141	Antigen	Non-Allergen	0.7388

### 3.8 Vaccine design and conservancy evaluation

**Figure 4:**
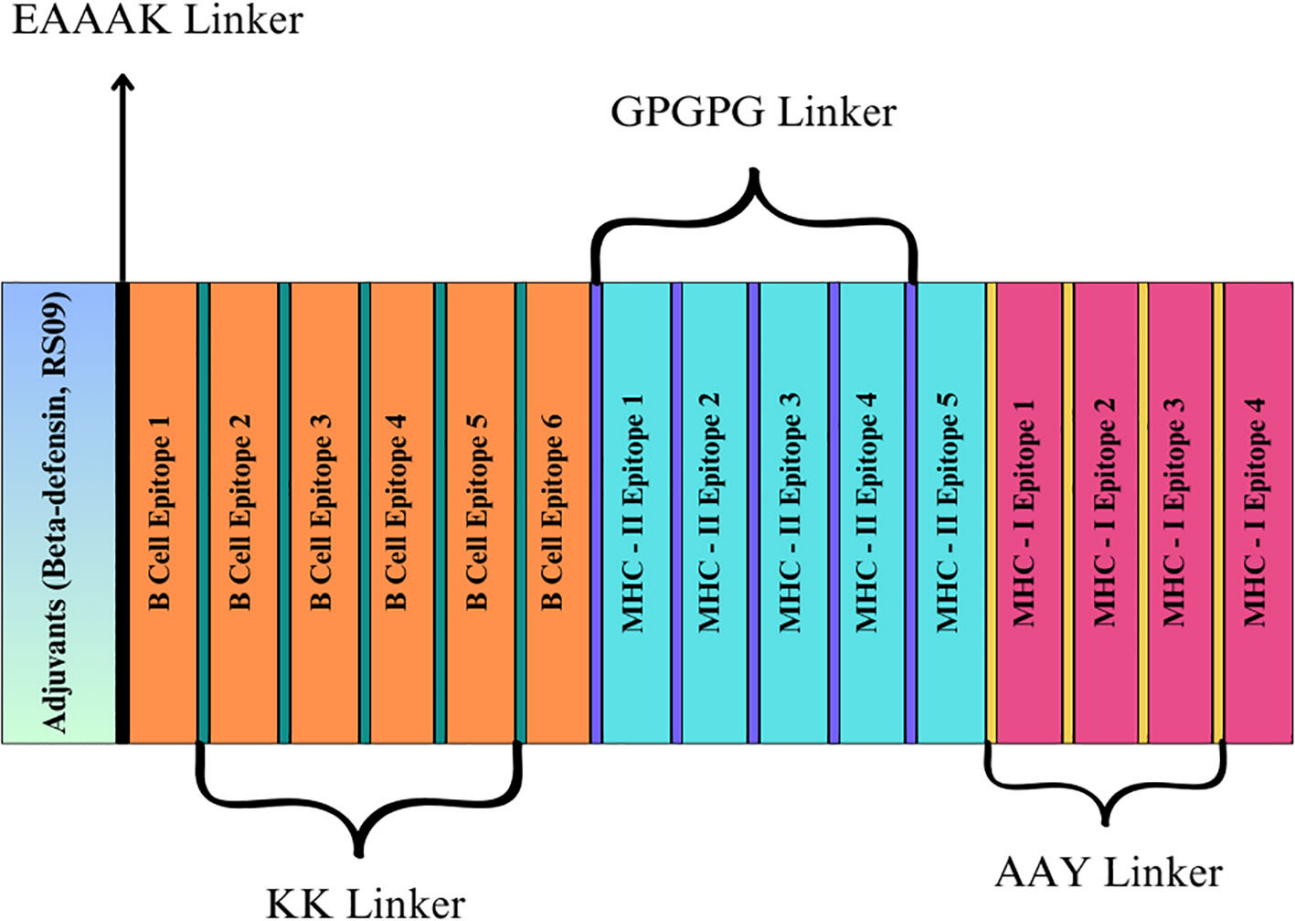
Graphical representation showcasing the formulation of the designed vaccine.

Two vaccine constructs were developed, incorporating adjuvants such as RS-09 and Beta-defensin and with the predicted epitopes from the LptF protein. Each vaccine included the selected epitopes, comprising five MHC-II, four MHC-I, and five B-cell epitopes. Fifteen epitopes were incorporated into the final vaccine constructs (**Figure 4**). The sequences and corresponding lengths of both constructions are described in **Table 5**, and the proposed vaccines ranged from 248 to 283 amino acids. The combined term for these constructs was POA_V. The presence of the chosen epitopes in *P. aeruginosa* was verified using a BLASTP analysis. The results demonstrated 100% sequence similarity across *P. aeruginosa* strains, indicating that the chosen epitopes are conserved and present in all strains.

**Table 5:** Amino acid sequences and sequence lengths of the finalized vaccine constructs.

POA_V	Sequence	Length
POA_V_BDEF	FTQGISNPSSCRRNRGFCLAFWCPGSMRQIGTCFGFPVKCCREAAAKSQLNAKQTKKGEDQR DVDQKKYGKEYPVASNGTSSGRAKKSAQRAQARLDKKDLDKSDLKPGKKLQSQPDATKVAAL ETKDAGDGPGPGVEVTISNDAKPVAPRGPGPGVLRNAEAQLQNASAQGPGPGEAQLQNASAQ RAQARGPGPGIVLRNAEAQLQNASAGPGPGTIVLRNAEAQLQNASAAYYTDSTGSANYAAYQT SRGTMVTFAAYGEDQRDVDQLAAYKSDLKPGAMR	283
POA_V_RS09	APPHALSEAAAKSQLNAKQTKKGEDQRDVDQKKYGKEYPVASNGTSSGRAKKSAQRAQARLDK KDLDKSDLKPGKKLQSQPDATKVAALETKDAGDGPGPGVEVTISNDAKPVAPRGPGPGVLRNAE AQLQNASAQGPGPGEAQLQNASAQRAQARGPGPGIVLRNAEAQLQNASAGPGPGTIVLRNAEA QLQNASAAYYTDSTGSANYAAYQTSRGTMVTFAAYGEDQRDVDQLAAYKSDLKPGAMR	248

### 3.9 Analysis of population coverage

**Figure 5:**
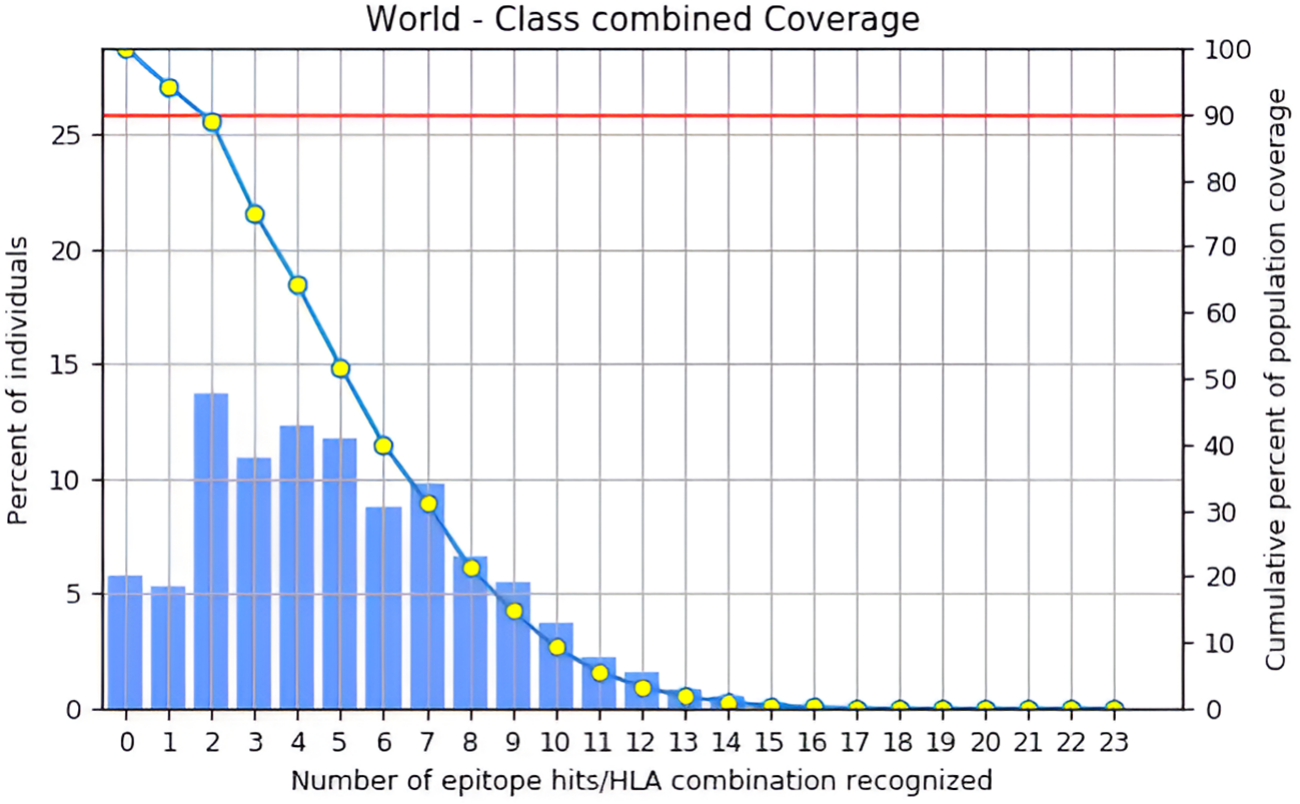
Global population coverage of the designed vaccine was analyzed using the IEDB tool, considering HLA allele frequencies across regions.

Based on estimated population coverage, the vaccine could potentially reach 87.35% of the global population. **Tables 3**, **4** comprehensively analyse the epitope distribution, demonstrating its adaptability across different regions and demographic groups. Additionally, **Figure 5** visually represents the global coverage, underscoring the vaccine’s potential for widespread impact (**Supplementary Table S6**, **Supplementary Figure S2**). Notably, regions such as the United States (98.33%), Kenya (98.58%), Germany (98.26%), Brazil (97.93%), France (98.04%), and Canada (95.58%) showed high predicted population coverage, emphasizing the vaccine’s potential effectiveness across diverse geographic and genetic backgrounds. Moderate coverage was observed in countries such as India (85.56%), Japan (87.60%), and China (89.81%), further confirming the vaccine’s adaptability in densely populated and genetically diverse regions. On the other hand, lower coverage was observed in regions such as the United Kingdom (56.38%), Hong Kong (56.64%), and American Samoa (56.40%), which may be attributed to regional HLA allele distribution patterns. Overall, the population coverage analysis strongly supports the broad usability and potential of the designed vaccine to fight the targeted pathogen worldwide.

### 3.10 Physicochemical property analysis

The physicochemical evaluation of the vaccine candidates POA_V_RS09 and POA_V_BDEF underscores their potential viability. POA_V_RS09, with a molecular weight of 25,734.39 Da comprising 248 amino acids, has an isoelectric point (pI) of 9.43. It exhibits hydrophilicity, as indicated by a GRAVY score of -0.856, and is considered stable with an instability index of 23.29. Similarly, POA_V_BDEF has a molecular weight of 29,763.15 Da, a pI of 9.53, and consists of 283 amino acids. Its instability index, 26.34, also suggests stability, and the GRAVY score of -0.761, which confirms its hydrophilic nature. These favorable stability and solubility properties render both candidates promising for further validation as vaccine prospects.

### 3.11 Secondary structure, tertiary structure, and refinement

The secondary structure of the vaccines was estimated using the PSIPRED approach, concentrating on the ratios of coils, β-sheets, and α-helices due to their immunogenic potential. PSIPRED’s analysis of the vaccine candidates POA_V_RS09 and POA_V_BDEF revealed distinct structural features. POA_V_RS09 comprised 58.87% alpha helices, 4.03% beta strands, and 37.10% random coils. In contrast, POA_V_BDEF consisted of 50.53% alpha helices, 7.07% beta strands, and 42.40% random coils (**Supplementary Figure S3**). These findings indicate a predominance of alpha helices and a significant presence of coils in both candidates, with a relatively low content of beta strands. This structural profile suggests a balance between stability and flexibility, which is beneficial for antigenic presentation in vaccine design. Using ROBETTA and AlphaFold, we modelled the vaccine’s 3D structures. Following structure generation, we refined all the models using GalaxyRefine to improve stereochemical accuracy. Among the generated models, Model 1 demonstrated superior performance for both vaccines, with RMSD values ranging from 0.9744 to 0.9889. Further validation was conducted using QMEAN4 scores and Ramachandran plot analysis to evaluate the structural quality at both global and local levels. For the POA_V_BDEF construct, the ROBETTA model yielded a QMEAN4 score of –0.72, while the AlphaFold model scored –2.67. Similarly, for the POA_V_RS09 construct, the ROBETTA model scored –0.19, compared to –2.24 for the AlphaFold prediction. QMEAN4 integrates four structural descriptors and is widely used to evaluate model quality in the absence of a native structure. These results indicate that the ROBETTA-generated models exhibit superior reliability and structural accuracy for both constructs. Further structural assessment using Ramachandran plot analysis (**Table 6**) revealed that ROBETTA models have over 96% of residues in favored regions, with only 0.71–0.81% falling in the disallowed areas. In contrast, AlphaFold models had a higher percentage of disallowed residues (up to 2.44%), particularly in functionally important loops and epitope-accessible regions. While AlphaFold has shown remarkable success in protein structure prediction and has been used in several recent vaccine design studies with promising results (78), we opted for ROBETTA-refined models in our research. This decision was based on comparative structural validation, where ROBETTA constructs exhibited fewer steric clashes and better Ramachandran statistics. Therefore, the ROBETTA-generated models were chosen for both POA_V_RS09 and POA_V_BDEF constructs and used in all downstream docking and immunological simulations to ensure structural reliability and predictive robustness. The Ramachandran plot of POA_V_RS09 and POA_V_BDEF shows that the structural value exceeds 90% of residues in favored regions, indicating a good overall geometry (**Supplementary Figure S4**). For the POA_V_BDEF construct, residues like Ser-26 and Gly-134 were located in disallowed areas, while for the POA_V_RS09 construct, residues Pro-100 and Gly-141 were also found in similar disallowed areas. The vaccine models are detailed in **Supplementary Figure S5**.

**Table 6:** Structural validation of POA_V_RS09 and POA_V_BDEF-based vaccine models generated using Robetta and AlphaFold.

Metric	Robetta (RS09)	AlphaFold (RS09)	Robetta (BDEF)	AlphaFold (BDEF)
Total residues	248	248	283	283
Favored regions	96.34%	91.46%	96.09%	92.53%
Allowed regions	2.85%	6.10%	3.20%	5.69%
Disallowed regions	0.81%	2.44%	0.71%	1.78%
Disallowed residues	Pro-100, Gly-141	Asp-28, Asp-30, Pro-138, Glu-227, Asp-228, Gln-229	Ser-26, Gly-134	Val-75, Leu-112, Pro-175, Gly-261, Gly-280
Quality	Better geometry & fewer outliers	More outliers	Better geometry	More outliers
QMEANDisCo Global Score	0.41 ± 0.05	0.55 ± 0.05	0.52 ± 0.05	0.48 ± 0.05
QMEAN	–0.19	–2.24	–0.72	–2.67

### 3.12 Molecular docking analysis

**Figure 6:**
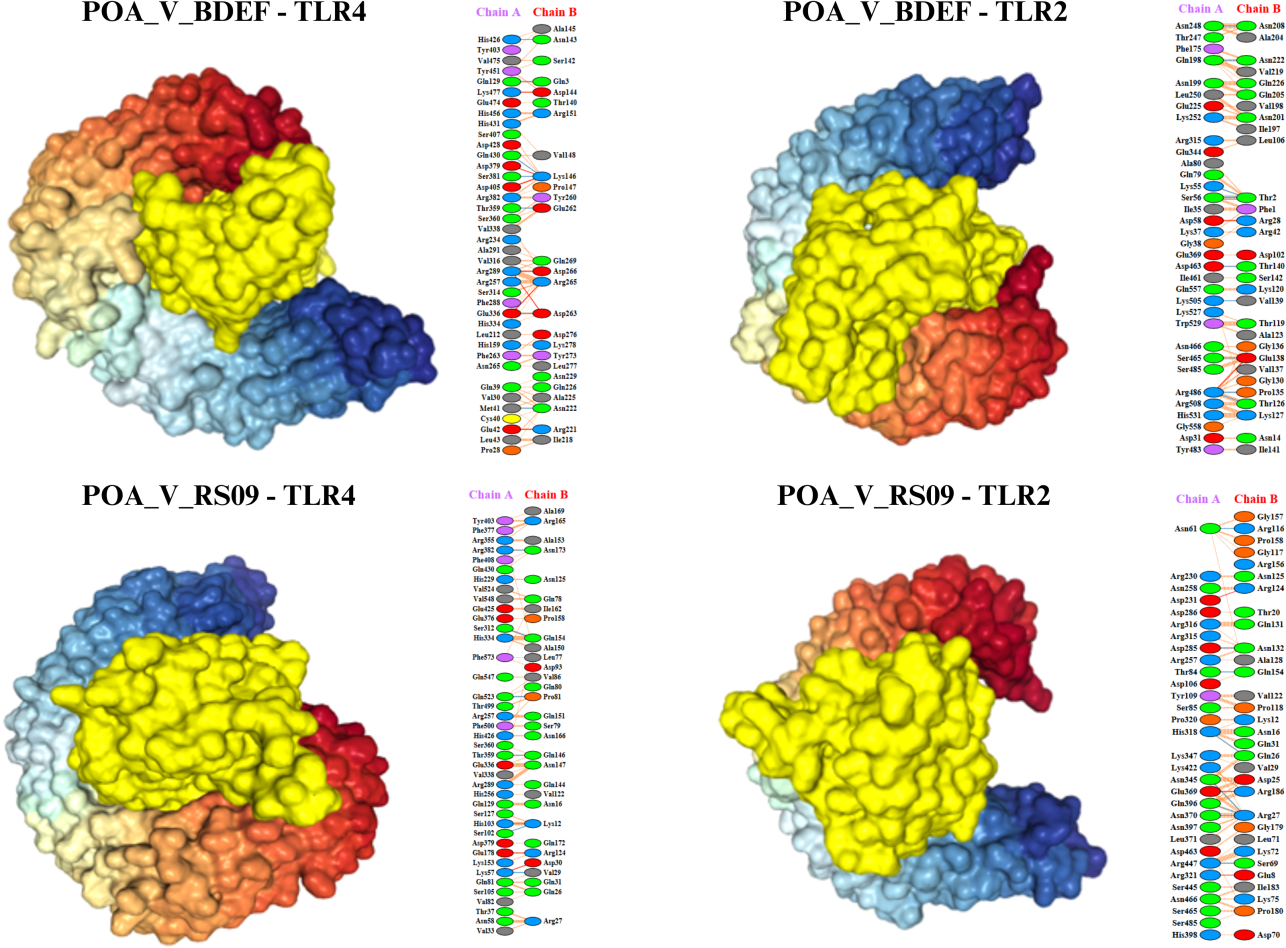
Illustrates the docking models of the vaccine constructs with the receptor, focusing on the lowest binding energy conformations. The identified interaction residues reveal strong binding affinities, highlighting critical contacts that contribute to the complex’s stability and potential efficacy.

The HDOCK server performed docking tests to evaluate the interactions between the suggested vaccine candidates POA_V_RS09 and POA_V_BDEF and the immunological receptors TLR2 and TLR4, respectively. These receptors are vital in recognizing pathogen-associated molecular patterns (PAMPs) and triggering immune responses, such as cytokine production and the recruitment of immune cells. The results indicated strong binding affinities for all complexes, with POA_V_RS09 achieving the highest docking scores of -310.2 (kcal/mol) for TLR4 and -286.76 (kcal/mol) for TLR2 (**Table 7**, **Figure 6**). The MD simulations were conducted to further validate the interactions by examining the stability and conformational behavior of the docked complexes under physiological conditions. Both vaccine candidates exhibited stable interactions, with minimal fluctuations at the receptor-binding interface, suggesting their ability to engage immune receptors and potentially elicit robust immune responses effectively. These findings highlight the promising immunogenic potential of the designed vaccines.

**Table 7:** Molecular docking scores of POA_V_BDEF and POA_V_RS09 with TLR2 and TLR4, showing binding affinities.

Rank	Docking Score (kcal/mol)	Confidence Score	Interface residues	Complex
1	-299.98	0.9526	model_1	TLR2 - POA_V_BDEF
1	-286.76	0.9391	model_1	TLR2 - POA_V_RS09
1	-305.23	0.9571	model_1	TLR4 - POA_V_BDEF
1	-310.2	0.961	model_1	TLR2 - POA_V_RS09

### 3.13 Molecular dynamics simulation analysis

**Figure 7:**
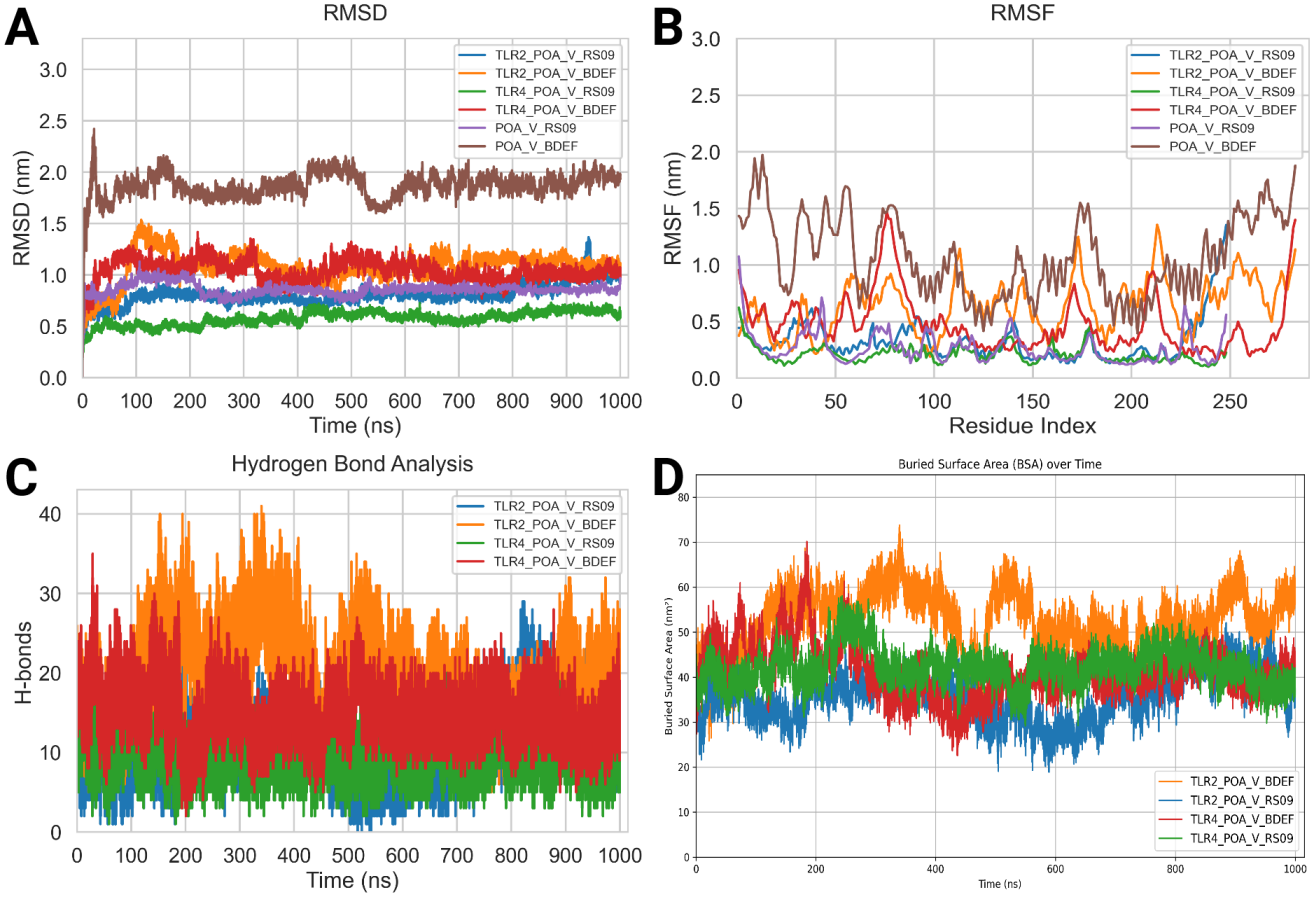
Molecular dynamics (MD) analysis results. **(A)** Root mean square deviation (RMSD) Backbone analysis. **(B)** Root mean square fluctuation (RMSF) Calpha Analysis. **(C)** Hydrogen bond (HBOND) analysis. **(D)** Buried surface analysis (BSA).

The MD simulations for the vaccine complexes (POA_V_RS09 and POA_V_BDEF) with TLR2 and TLR4 were conducted over 1000 ns and revealed notable differences in stability and interaction properties (**Table 8**). The RMSD (backbone) value indicated that the POA_V_RS09 vaccine complex was the most stable, with the TLR4_POA_V_RS09 complex showing the lowest RMSD (0.57 ± 0.06 nm), followed by TLR2_POA_V_RS09 (0.80 ± 0.19) nm. These complexes remained stable throughout the 1000-ns MD simulation. In contrast, the POA_V_BDEF-based vaccine complexes had higher RMSD values, with TLR2_POA_V_BDEF (1.08 ± 0.14 nm) and TLR4_POA_V_BDEF (1.03 ± 0.10 nm), indicating more significant structural deviations and less stable interactions (**Figure 7A**). These complexes slightly fluctuated at the beginning of the MD simulation (0-200ns), and later they equilibrated at 1 nm. To investigate the observed fluctuations, we analyzed the backbone RMSD and Calpha RMSF of TLR2 and TLR4. Both receptors exhibited considerable structural stability, with average RMSD values of TLR2 in POA_V_BDEF at (0.33 ± 0.03 nm), TLR2 in POA_V_RS09 at (0.40 ± 0.07 nm), TLR4 in POA_V_BDEF at (0.26 ± 0.03 nm), and TLR4 in POA_V_RS09 at (0.23 ± 0.03 nm). The RMSF profiles also indicated stable conformations across all complexes, TLR2 in POA_V_BDEF at (0.15 ± 0.08 nm), TLR2 in POA_V_RS09 at (0.16 ± 0.14 nm), TLR4 in POA_V_BDEF at (0.15 ± 0.07 nm), and TLR4 in POA_V_RS09 at (0.14 ± 0.07 nm), as illustrated in **Supplementary Figure S6**. The predicted POA_V_BDEF complex displayed enhanced flexibility, primarily attributed to the presence of less structured epitope and linker regions **Supplementary Figure S7**. This inherent structural looseness likely accounts for the comparatively elevated average RMSD observed across its associated complexes. When coming to the vaccine stability in residue wise, RMSF (Calpha) analysis showed that the RS09 vaccine complexes were more rigid, with the TLR4_POA_V_RS09 complex showing the lowest RMSF (0.21 ± 0.07 nm) and the TLR2_POA_V_RS09 complex showing (0.32 ± 0.18 nm), indicating minimal flexibility at the interaction interface and that all the residues were around 0.5nm. Conversely, the POA_V_BDEF-based vaccine complexes had higher RMSF values, with TLR2_POA_V_BDEF (0.64 ± 0.23 nm) and TLR4_POA_V_BDEF (0.47 ± 0.25 nm), suggesting increased flexibility and dynamic behaviour (**Figure 7B**). In contrast, the POA_V_BDEF vaccine construct exhibited pronounced fluctuations, particularly in regions interacting with TLR4 and TLR2. For the TLR4_POA_V_BDEF complex, notable flexibility was observed in the N-terminal linker region (residues 53–57), as well as in combined epitope and linker segments spanning residues 50–85, 165–180, and 207–216, in addition to the C-terminal end. Similarly, the TLR2_POA_V_BDEF complex showed continuous fluctuation across the linker (55–60), the epitope region (73–83), the extended linker–epitope stretch (109–150), and residues 173–180 and 195–220, along with the C-terminal region. In contrast to the POA_V_BDEF construct, the POA_V_RS09-based vaccine formulation demonstrated notably greater structural stability.

**Table 8:** Post-MD analysis averages for protein-protein complexes, including RMSD, RMSF, and H-bond values, reflecting structural stability and interactions.

Complexes	RMSD (nm)	RMSF (nm)	Avg. H-bond
POA_V_RS09_TLR2	0.80 ± 0.19	0.32 ± 0.18	11
POA_V_RS09_TLR4	0.57 ± 0.06	0.21 ± 0.07	10
POA_V_BDEF_TLR2	1.08 ± 0.14	0.64 ± 0.23	19
POA_V_BDEF_TLR4	1.03 ± 0.10	0.47 ± 0.25	14
POA_V_RS09_APO	0.83 ± 0.10	0.26 ± 0.13	–
POA_V_BDEF_APO	1.75 ± 0.25	1.07 ± 0.35	–

PCA was performed on the vaccine constructs extracted from their respective TLR2 and TLR4 complexes to evaluate their conformational dynamics. The POA_V_RS09 construct, when analyzed post-interaction with both TLR2 and TLR4, exhibited compact PCA clusters, indicating limited conformational fluctuations and stable structural behavior throughout the 1000 ns simulation. In contrast, the POA_V_BDEF construct displayed broader dispersions in PCA space, suggesting greater structural flexibility and reduced conformational stability. This trend remained consistent when the standalone vaccine models were analyzed, where POA_V_RS09 continued to show tight clustering and structural integrity, while POA_V_BDEF exhibited higher variability. These results align with earlier RMSD and RMSF analyses, collectively highlighting POA_V_RS09 as the more stable and potentially immunogenic vaccine candidate. (**Supplementary Figure S8**). Hydrogen bond (HBOND) analysis was conducted over the 1000 ns molecular dynamics simulation using GROMACS. The default criteria were used, which include a donor–acceptor distance cutoff of 0.35 nm and a hydrogen–donor–acceptor angle cutoff of ≥150° (i.e., ≤30° deviation from linearity), consistent with established definitions for biologically relevant hydrogen bonds. The analysis focused specifically on the intermolecular hydrogen bonds formed between the vaccine constructs and the TLR receptors. The POA_V_BDEF vaccine complexes exhibited a higher average number of hydrogen bonds (19 with TLR2 and 14 with TLR4) compared to the POA_V_RS09 complexes (11 with TLR2 and 10 with TLR4). However, the relatively higher RMSD and RMSF values observed in the POA_V_BDEF complexes suggest that these additional hydrogen bonds may be less stable or more transient (**Figure 7C**). The buried surface area (BSA) during the 1000 ns simulation at the interface of the TLR4_POA_V_RS09 complex was 42.09 nm² ± 3.98, indicating stable interactions and low variability. This was closely followed by TLR4_POA_V_BDEF, with a BSA of 41.12 nm² ± 5.83, showing a similar interaction pattern. In contrast, TLR2_POA_V_BDEF had a higher BSA of 52.37 nm² ± 6.60, while TLR2_POA_V_RS09 showed a BSA of 35.70 nm² ± 5.19, both with higher standard deviations, suggesting relatively fewer stable interactions (**Figure 7D**). Analysis of the apo forms revealed that POA_V_BDEF exhibited the highest RMSD (1.75 ± 0.25 nm) and RMSF (1.07 ± 0.35 nm), indicating significant conformational flexibility in the absence of receptor binding. In contrast, POA_V_RS09 exhibited lower deviation (0.83 ± 0.10 nm RMSD and 0.26 ± 0.13 nm RMSF), suggesting it remains relatively stable even when unbound. The FEL analysis effectively showed us the structural stability and flexibility of the vaccine-protein complexes. All complexes exhibited energy basins, indicating the presence of metastable states. However, notable differences were observed in the shape and depth of these energy wells. Complexes involving TLR4 exhibited more compact and deeper energy minima compared to those involving TLR2, suggesting a higher degree of structural stability. In particular, the TLR4_POA_V_RS09 complex exhibited a well-defined global minimum, indicating a stable and energetically favorable conformation throughout the simulation. Although the TLR4_POA_V_BDEF complex also reached stable conformations, it showed slightly more conformational variability. Conversely, the TLR2 complexes exhibited broader and more scattered low-energy regions, indicating increased conformational flexibility and less stable interaction patterns. Among them, the TLR2_POA_V_RS09 complex exhibited relatively smoother energy transitions compared to TLR2_POA_V_BDEF, which displayed more rugged features in its energy landscape (**Supplementary Figure S9**).

We calculated the binding free energy using MMPBSA, which revealed notable differences between the POA_V_RS09 and POA_V_BDEF-based vaccine constructs in complex with TLR2 and TLR4 receptors. The TLR2_POA_V_RS09 showed a more favorable binding energy (–1483.14 kJ/mol) than TLR2_POA_V_BDEF (–1335.16 kJ/mol), indicating that RS09 forms a more stable and energy-efficient complex with TLR2, compared to POA_V_BDEF. In contrast, TLR4_POA_V_BDEF exhibited a significantly stronger binding energy (–4600.83 kJ/mol) than TLR4_RS09 (–2682.66 kJ/mol), likely due to its extended area, which enables an increased contact surface. However, prior dynamic and structural analyses, such as RMSF and FEL plots, indicate that POA_V_BDEF is more flexible, particularly at the linker and epitope regions. This flexibility may contribute to reduced structural stability, especially in the TLR2 complex compared to POA_V_RS09.

### 3.14 *In silico* codon adaptation, cloning, and immune simulation

**Figure 8:**
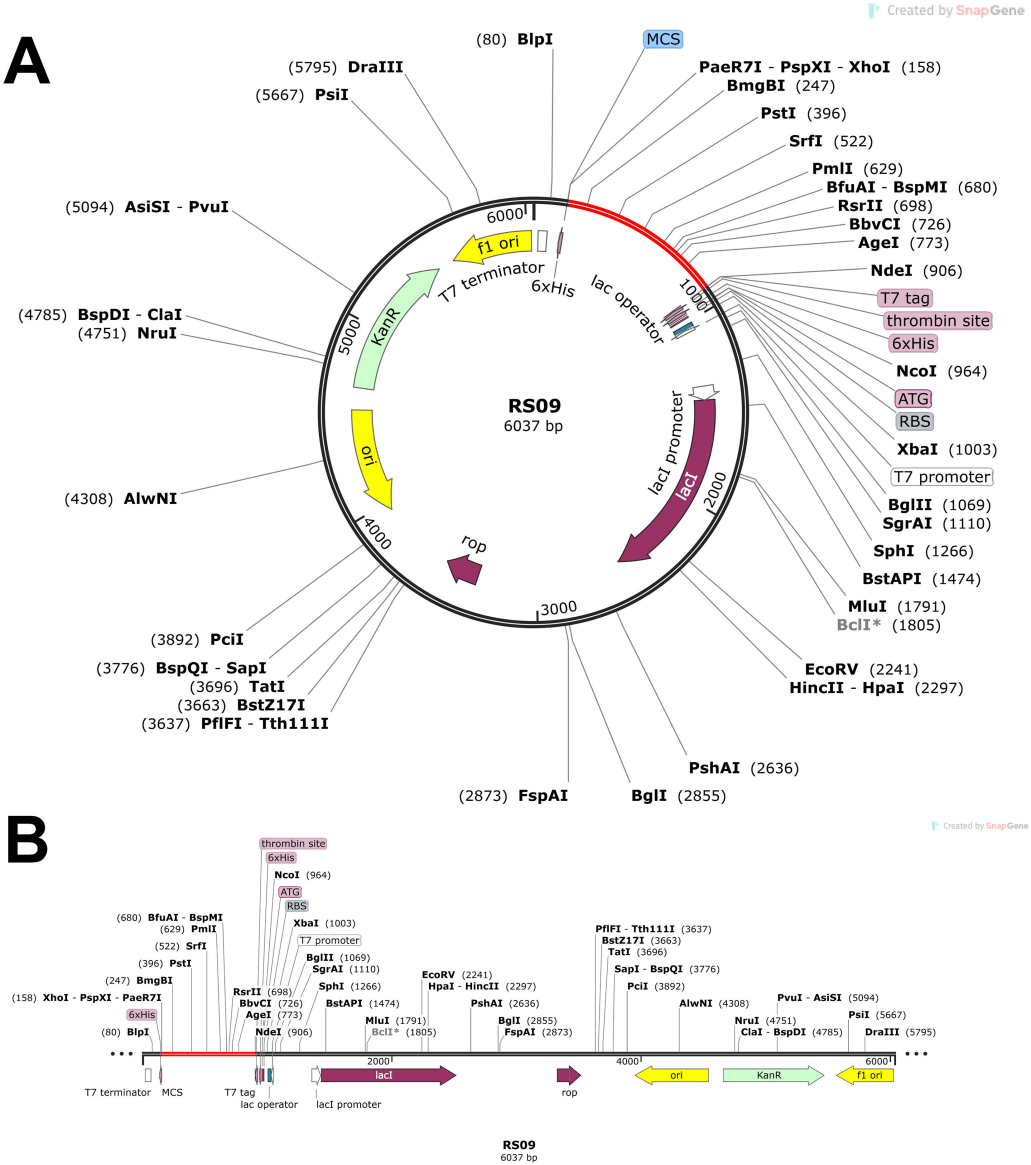
*In silico* cloning of POA_V_RS09. **(A)** Cloning of RS09 (which is POA_V_RS09) into the pET-28a(+) vector. **(B)** final vaccination design with additional restriction sites.

**Figure 9:**
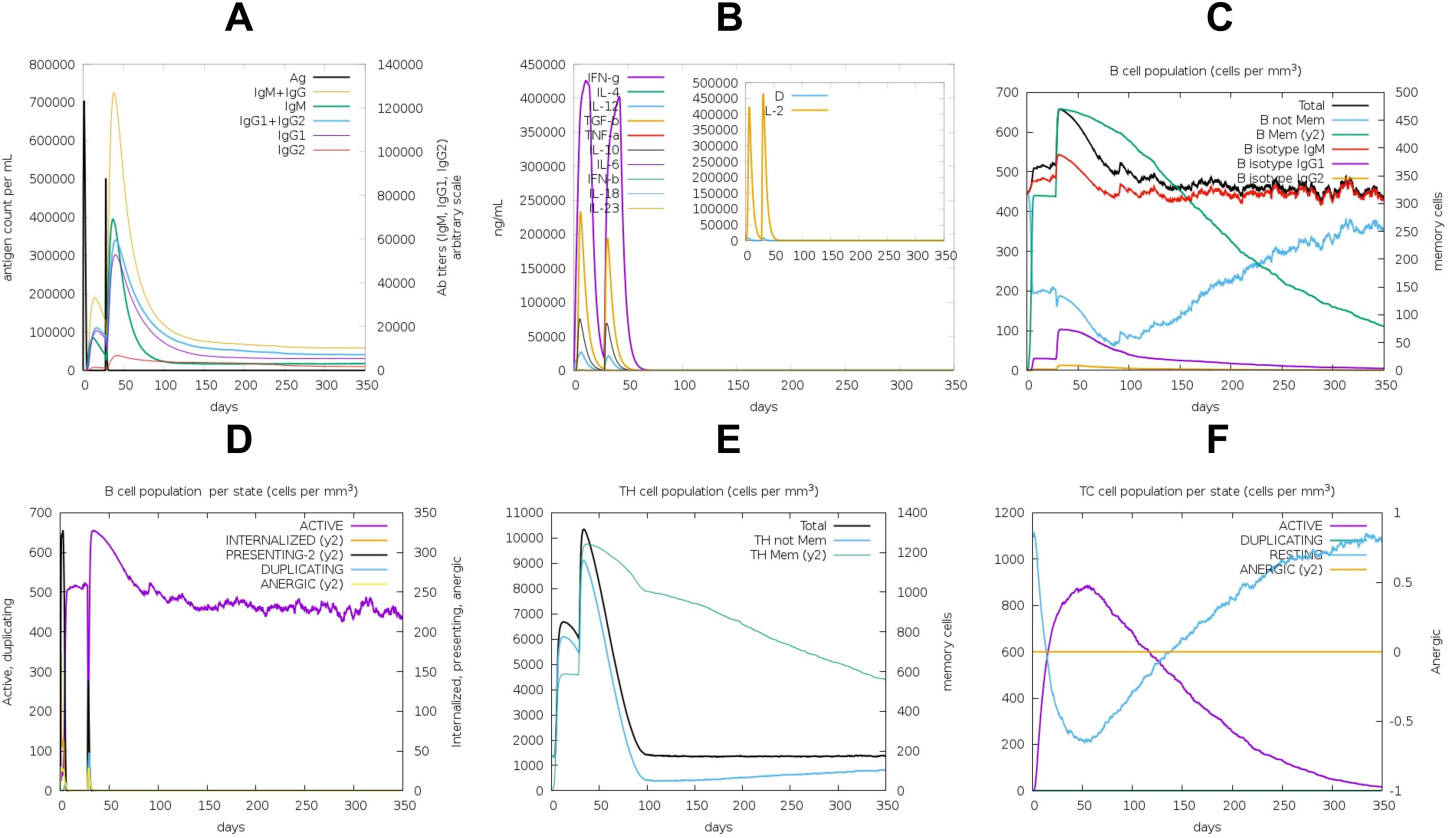
Immune Response Induced by the POA_V_RS09 Vaccine: **(A)** antibody response. **(B)** Cytokine response. **(C)** B-Cell population (cells/mm³). **(D)** B-Cell population by state. **(E)** TH-cell population by state. **(F)** TC-cell population by state.

Codon optimization was performed using GenScript to enhance the expression of the POA_V_RS09 vaccine sequence in *E. coli* K-12. With a GC content of 60.22% and a total length of 744 base pairs, the optimized sequence falls within the ideal range (30–70%) for effective expression in *E. coli*. This balanced GC content ensures efficient transcription and translation, making the sequence suitable for high-level expression in the host. The optimized vaccine sequence was then used for in-silico cloning with SnapGene software, successfully inserting the gene into the pET-28a(+) expression plasmid (**Figure 8**). The immune response dynamics elicited by POA_V_RS09 are shown in **Figure 9**. **Figure 9A** illustrates the antigen (Ag) and antibody responses over a 350-day period, where an early antigen peak, followed by a sharp decline, indicates effective recognition and clearance by the host immune system. This is accompanied by a strong humoral response, characterized by an initial surge in IgM, typical of a primary response, followed by a sustained increase in IgG1 and IgG2, which shows class switching and maturation of the immune response. The dominance of IgG subclasses over time reflects the development of long-term protective immunity. Notably, IgG1 and IgG2 are associated with Th1-type immune responses, which are essential for combating pathogens such as viruses and certain bacteria. **Figure 9B** illustrates cytokine dynamics, where high levels of Interleukin-2 (IL-2) and IFN-γ early on indicate strong T-cell activation and a Th1-biased immune response, which is particularly important for combating pathogens. Their gradual decline over time suggests immune regulation and resolution of inflammation, highlighting the vaccine’s safety profile. **Figure 9C** illustrates the dynamics of the B-cell population, exhibiting an increasing trend in memory B cells and a shift in isotype expression from IgM to IgG, which further validates class switching and the generation of long-lasting humoral memory. **Figure 9D** focuses on B-cell states, showing that active and antigen-internalizing B cells peak early, while anergic cells remain relatively constant, suggesting efficient antigen processing and presentation. The low level of anergic B cells suggests that immune tolerance is not induced, further supporting the vaccine’s immunogenicity. **Figure 9E** presents helper T-cell (TH) populations, with an initial spike in total TH cells, followed by the emergence of memory TH cells, supporting long-term immunity. Finally, **Figure 9F** depicts cytotoxic T-cell (TC) states, showing early activity and duplication followed by a steady increase in resting memory TC cells, which are critical for sustained immune surveillance. This can contribute to the direct killing of bacteria or infected host cells in bacterial infections that evade extracellular immune mechanisms. These outcomes underscore the successful initiation of both humoral and cellular immune responses, supporting the potential for the development of long-lasting immunological memory. Moreover, the observed immune signatures align with the protective responses typically seen in effective bacterial vaccines, validating the rational design of POA_V_RS09, which incorporates TLR agonists, multi-epitope constructs, and immune-enhancing linkers to induce broad, durable immunity against bacterial pathogens.

## 4 Discussion

The opportunistic pathogen *P. aeruginosa* is a significant cause of hospital-acquired infections worldwide. It presents a serious threat to human health, especially in immunocompromised individuals, due to its innate antibiotic resistance and ability to develop biofilms (79, 80). The increasing prevalence of drug-resistant strains has complicated treatment options, highlighting the urgent need for alternative therapeutic strategies (81). Despite progress in antimicrobial therapies, no licensed vaccine exists for *P. aeruginosa*, revealing a critical gap in combating this pathogen (82). Immunoinformatics has emerged as a powerful tool in vaccine development, facilitating the rational design of in silico vaccines, as demonstrated in the development of vaccines against pathogens such as the Ebola virus, SARS-CoV-2, and Mycobacterium tuberculosis (83–85). These approaches hold significant promise for addressing the challenges posed by *P. aeruginosa*.

Previous immunoinformatics-based vaccine studies against *P. aeruginosa* often relied on single-strain datasets or focused on a narrow range of targets. Some selected cytoplasmic proteins have limited surface accessibility, while others used previously known antigens without assessing their conservation across diverse strains. Additionally, several studies selected targets which is completely based on literature without genome-wide screening (86–89). Other broader approaches involving multiple pathogens have also identified shared virulence or essential gene-derived epitopes while filtering for self-tolerance (90). However, such strategies typically lack species-specific optimization, structural validation, and comprehensive strain-level genomic coverage—critical aspects that our study addresses. In this study, we conducted a comprehensive pangenome analysis (91) of 864 *P. aeruginosa* genomes. This extensive dataset enabled robust pangenome analysis and the identification of conserved, surface-exposed, and virulence-associated targets, distinguishing our study from previous investigations. Here we identified 63,239 genes, including 3,325 core genes and 3,149 accessory genes. We focused on conserved core genes essential for bacterial survival and pathogenicity to ensure broad-spectrum coverage. The Pal_1 protein was identified as a potential vaccine candidate, which is classified as an outer membrane protein via PSORTb, and confirmed as a virulence-associated factor through BLASTP analysis against the VFDB. Its sequence was validated against the *P. aeruginosa* database, where it was identified with 100% confidence and an E-value of 0, and it is known as LptF (lipotoxin F). Sequence comparison with the human proteome confirmed the absence of homologous hits, minimizing the risk of adverse cross-reactivity. LptF, an OmpA-like outer membrane protein, plays a crucial role in *P. aeruginosa*’s survival, particularly in stressful environments such as lung colonization in cystic fibrosis, and may serve as an important target for therapeutic strategies (92). LptF remains an underexplored target. Its classification as a lipotoxin, along with evidence from structural proteomics revealing interactions with key membrane proteins like OprI and LptE, further highlights its relevance as a promising vaccine candidate against *P. aeruginosa.* Due to the increasing antibiotic resistance of *P. aeruginosa*, an effective vaccine is urgently needed, and lipotoxins have been identified as potential targets in studies (24). The LptF protein exhibited favorable physicochemical and immunogenic properties, with a molecular weight of 28.5 kDa, thermostability indicated by an aliphatic index of 80.15, and a hydrophilic nature reflected in a GRAVY index of -0.574. Immunogenic analysis revealed its suitability as a vaccine target, with an antigenicity score of 0.6442 and classification as a non-allergen. SignalP analysis showed that the protein has a signal peptide, which is likely removed during maturation. This was accounted for in later analyses that focused on the mature protein sequence. We identified B-cell epitopes and chose high-affinity T-cell epitopes based on their binding affinities to MHC-I and MHC-II molecules, with additional refinement based on their potential to stimulate interferon-γ production. Ultimately, 15 epitopes were incorporated into the vaccine design, comprising four MHC-I epitopes, five MHC-II epitopes, and six B-cell epitopes. Additionally, the cytokine prediction analysis revealed that all selected epitopes possess IL-10-inducing potential, a cytokine shown to be critical in controlling inflammation and enhancing bacterial clearance during *P. aeruginosa* infection (93). The vaccine constructs POA_V_RS09 and POA_V_BDEF were designed, incorporating RS-09 and Beta-defensin as adjuvants. We selected RS09 as it functions as a TLR4 agonist, effectively stimulating the innate immune response. β-defensin was chosen for its dual role in activating both innate and adaptive immunity. Incorporating these adjuvants aimed to enhance the immunogenic potential of the constructs. This strategic design allowed us to evaluate and compare their impact on vaccine performance. These constructs exhibited broad global population coverage (87.35%) and strong immunogenic potential. Secondary structure analysis revealed that both vaccines predominantly consisted of α-helices and random coils, enhancing antigenic presentation. Structural validation through Ramachandran plot analysis ensured the reliability of the tertiary structures. We selected the Robetta server over AlphaFold for tertiary structure prediction because Robetta offers more reliable modeling for synthetic, chimeric constructs involving multiple domains, such as adjuvants, epitopes, and linkers. Unlike AlphaFold, which is optimized for natural protein sequences, Robetta’s *de novo* prediction approach is better suited for handling novel vaccine constructs. For the BDEF-based construct, serine-26 (Ser-26) and glycine-134 (Gly-234) residues were found in disallowed regions, while the RS09-based construct showed proline-100 (Pro-100) and glycine-141 (Gly-141) residues in disallowed regions. These residues were primarily located in loop and linker regions and were not associated with key epitope or adjuvant domains, suggesting that they are unlikely to compromise the overall structural integrity of the protein. Therefore, the refined and validated vaccine structures were subsequently used for molecular docking studies to assess receptor interactions. Molecular docking analyses revealed high-affinity interactions between the vaccine constructs and Toll-like receptors TLR2 and TLR4, which play pivotal roles in initiating innate immune responses. POA_V_RS09 showed superior docking scores, with -310.2 (kcal/mol) for TLR4 and -286.76 (kcal/mol) for TLR2, while MD simulations confirmed the stability of these interactions under physiological conditions. We conducted extensive 1000-ns molecular dynamics simulations to analyze the long-term structural stability and interaction dynamics of the vaccine-receptor complexes. This extended simulation duration exceeds the standard practice in similar studies, providing deeper insights into conformational behavior, particularly in flexible regions such as linkers and epitopes. It enhances the structural validation of our vaccine constructs and reinforces the reliability of our results. The TLR4_POA_V_RS09 complex demonstrated minimal structural fluctuations, with the lowest RMSD and RMSF values, indicating stable interactions compared to POA_V_BDEF. Although POA_V_BDEF demonstrated good structural quality based on validation metrics, MD simulations revealed considerable flexibility, even in its apo form (without receptor binding). This inherent flexibility, especially in the epitope-linker regions, might weaken stable receptor binding and influence immune activation. While some mobility facilitates epitope presentation, too much fluctuation can reduce vaccine effectiveness. These findings underscore the importance of dynamic assessment in conjunction with static validation when evaluating multi-epitope vaccine designs. Further analysis confirmed POA_V_RS09’s stable conformational states, with tighter cluster dispersion and distinct energy minima. MMPBSA analysis showed that although POA_V_BDEF has a strong binding affinity with TLR4, POA_V_RS09 exhibits a more balanced and consistent interaction profile with both TLR2 and TLR4, along with fewer structural fluctuations. These qualities make POA_V_RS09 a robust and dependable adjuvant candidate for the design of multi-epitope vaccines. Codon optimization for POA_V_RS09 allowed efficient expression in *E. coli* (K-12), and in silico cloning into pET-28a(+) validated its expression potential. Immune simulations demonstrated robust adaptive immune responses, characterized by sustained IgG production, memory B-cell formation, and effective cytokine engagement, rendering POA_V_RS09 a promising vaccine candidate for long-term immunity. The POA_V_RS09 vaccine demonstrated strong stability, optimal expression potential, and robust immune activation, positioning it as an ideal candidate for further development. By targeting *P. aeruginosa*, a highly resistant pathogen, the POA_V_RS09 vaccine could offer a valuable strategy for preventing infections and addressing the global threat of antimicrobial resistance, ultimately improving patient outcomes. In this study, we designed two separate vaccine constructs using RS09 and β-defensin adjuvants to independently evaluate their immunostimulatory potential. This separation allows for comparative assessment of construct stability, population coverage, and immunogenicity. However, future studies could explore the integration of both adjuvants into a single construct, as combinatorial adjuvants have been shown to enhance immune responses more effectively than individual components (78). One limitation of this study is that it lacks experimental validation. While our computational approach provides a cost-effective and time-efficient method for epitope screening, future *in vitro* and *in vivo* studies (e.g., ELISA, ELISPOT) are essential to confirm immunogenicity and support vaccine development of the POA_V_RS09 vaccine candidate against *P. aeruginosa*. However, we have thoroughly examined the structural and immunological characteristics of the vaccine candidate through in silico methods, including 1000 ns molecular dynamics simulations, epitope mapping, TLR docking, population coverage analysis, and immunogenicity prediction. Long-timescale MD simulations allow for the capture of biologically relevant conformational changes, showing that microsecond to millisecond scale simulations can uncover protein folding pathways and slow structural transitions. This supports the use of 1000 ns MD to study dynamic molecular interactions (94). Several previous studies have demonstrated that immunoinformatics-based vaccine designs can reliably predict antigenic determinants and immune interactions, often correlating well with experimental outcomes (95–97). These findings support the translational relevance of computational predictions in the early stages of vaccine design. Further validation using comprehensive *in vitro* assays is also necessary to evaluate the safety profile and immunogenic potential of the POA_V_RS09-based vaccine, including its ability to induce pro-inflammatory cytokines, activate T cells, and generate specific antibody responses. Such investigations will provide valuable insights into the clinical feasibility of POA_V_RS09 as a vaccine candidate for *P. aeruginosa* infections.

## 5 Conclusion

This study utilized an integrated pangenome and immunoinformatics approach to develop an epitope-based peptide vaccine targeting *P. aeruginosa*. Through pangenome analysis, we identified LptF as a promising and underexplored vaccine target, specifically. From LptF, we predicted potential epitopes. The resulting vaccine candidate, POA_V_RS09, demonstrated promising immune response outcomes and strong binding affinity to immunological receptors (TLRs). Notably, the 1000-ns molecular dynamics simulation provided valuable insights into the structural stability of the vaccine–receptor complexes over an extended timescale, reinforcing the robustness of the construct under physiological conditions. This computational strategy holds significant potential for addressing the escalating issue of antimicrobial resistance, particularly in resource-limited settings and low-income countries. This strategy provides a comprehensive and practical approach to combating infections by targeting conserved NS proteins, identifying high-affinity B-cell and T-cell epitopes, and utilizing suitable adjuvants. Future studies should assess the vaccine’s safety, effectiveness, and scalability through *in vitro* investigations, animal model testing, and ensuing clinical trials. To transform this computational framework into a valuable tool for combating *P. aeruginosa* resistance to multiple drugs, these steps are crucial.

## Data Availability

The original contributions presented in the study are included in the article/**Supplementary Material**. Further inquiries can be directed to the corresponding author.
