# Supplementary material for: Rational design of an epitope-centric vaccine against Pseudomonas aeruginosa using pangenomic insights and immunoinformatics approach
Source: Front Immunol. 2025 Sep 1;16:1617251. doi: 10.3389/fimmu.2025.1617251 (PMC12434008; doi:10.3389/fimmu.2025.1617251)
Supplement: Supplementary file 1 [file Table1.docx]

**Rational Design of an Epitope-Centric Vaccine Against *Pseudomonas aeruginosa* using Pangenomic Insights and Immunoinformatics Approach**

**Supplementary Table 1: Collection of *Pseudomonas aeruginosa* data from the NCBI Database**

| **Data Retrieval from NCBI Database** | | | |
| --- | --- | --- | --- |
| Asembly Accession | Organism Name | Strain Name | Assembly Level |
| GCF_000829885.1 | *Pseudomonas aeruginosa* | strain=FRD1 | Complete Genome |
| GCF_000829275.1 | *Pseudomonas aeruginosa* | strain=NCGM1900 | Complete Genome |
| GCF_000981825.1 | *Pseudomonas aeruginosa* | strain=Carb01 63 | Complete Genome |
| GCF_000829255.1 | *Pseudomonas aeruginosa* | strain=NCGM1984 | Complete Genome |
| GCF_001077475.1 | *Pseudomonas aeruginosa* | strain=F9676 | Complete Genome |
| GCF_000816985.1 | *Pseudomonas aeruginosa* | strain=F22031 | Complete Genome |
| GCF_001293085.1 | *Pseudomonas aeruginosa* | strain=PA1RG | Complete Genome |
| GCF_001516385.1 | *Pseudomonas aeruginosa* | strain=M37351 | Complete Genome |
| GCF_001518975.1 | *Pseudomonas aeruginosa* | strain=USDA-ARS-USMARC-41639 | Complete Genome |
| GCF_001516345.1 | *Pseudomonas aeruginosa* | strain=H47921 | Complete Genome |
| GCF_001516005.1 | *Pseudomonas aeruginosa* | strain=T52373 | Complete Genome |
| GCF_001516105.1 | *Pseudomonas aeruginosa* | strain=T63266 | Complete Genome |
| GCF_001457615.1 | *Pseudomonas aeruginosa* | strain=NCTC10332 | Complete Genome |
| GCF_001482325.1 | *Pseudomonas aeruginosa* | strain=12-4-4(59) | Complete Genome |
| GCF_001548335.1 | *Pseudomonas aeruginosa* | strain=IOMTU 133 | Complete Genome |
| GCF_001516265.1 | *Pseudomonas aeruginosa* | strain=F30658 | Complete Genome |
| GCF_001548135.1 | *Pseudomonas aeruginosa* | strain=8380 | Complete Genome |
| GCF_001547955.1 | *Pseudomonas aeruginosa* | strain=NCGM257 | Complete Genome |
| GCF_001447845.1 | *Pseudomonas aeruginosa* | strain=VA-134 | Complete Genome |
| GCF_001542835.1 | *Pseudomonas aeruginosa* | strain=F9670 | Complete Genome |
| GCF_001618925.1 | *Pseudomonas aeruginosa* | strain=ATCC 27853 | Complete Genome |
| GCF_001687285.1 | *Pseudomonas aeruginosa* | strain=ATCC 27853 | Complete Genome |
| GCF_001606045.1 | *Pseudomonas aeruginosa* | strain=N17-1 | Complete Genome |
| GCF_001632245.1 | *Pseudomonas aeruginosa* | strain=BAMCPA07-48 | Complete Genome |
| GCF_001722045.1 | *Pseudomonas aeruginosa* | strain=PA_D21 | Complete Genome |
| GCF_001721805.1 | *Pseudomonas aeruginosa* | strain=PA_D16 | Complete Genome |
| GCF_900095805.1 | *Pseudomonas aeruginosa* | na | Complete Genome |
| GCF_001750705.1 | *Pseudomonas aeruginosa* | strain=FA-HZ1 | Complete Genome |
| GCF_001679685.1 | *Pseudomonas aeruginosa* | strain=PA121617 | Complete Genome |
| GCF_001729505.1 | *Pseudomonas aeruginosa* | strain=ATCC 15692 | Complete Genome |
| GCF_001722025.1 | *Pseudomonas aeruginosa* | strain=PA_D5 | Complete Genome |
| GCF_001721765.1 | *Pseudomonas aeruginosa* | strain=PA_D2 | Complete Genome |
| GCF_001721745.1 | *Pseudomonas aeruginosa* | strain=PA_D1 | Complete Genome |
| GCF_001721845.1 | *Pseudomonas aeruginosa* | strain=PA_D25 | Complete Genome |
| GCF_001721785.1 | *Pseudomonas aeruginosa* | strain=PA_D9 | Complete Genome |
| GCF_001792875.1 | *Pseudomonas aeruginosa* | strain=PA11803 | Complete Genome |
| GCF_001792855.1 | *Pseudomonas aeruginosa* | strain=PA8281 | Complete Genome |
| GCF_001874465.1 | *Pseudomonas aeruginosa* | na | Complete Genome |
| GCF_001879525.1 | *Pseudomonas aeruginosa* | strain=Paer4_119 | Complete Genome |
| GCF_001721825.1 | *Pseudomonas aeruginosa* | strain=PA_D22 | Complete Genome |
| GCF_001792835.1 | *Pseudomonas aeruginosa* | strain=PA1088 | Complete Genome |
| GCF_001870265.1 | *Pseudomonas aeruginosa* | strain=PA7790 | Complete Genome |
| GCF_001900225.1 | *Pseudomonas aeruginosa* | strain=SCVJan | Complete Genome |
| GCF_900070375.1 | *Pseudomonas aeruginosa* | strain=PAO1_Orsay | Complete Genome |
| GCF_001900265.1 | *Pseudomonas aeruginosa* | strain=NHmuc | Complete Genome |
| GCF_001900195.1 | *Pseudomonas aeruginosa* | strain=SCVFeb | Complete Genome |
| GCF_900149285.1 | *Pseudomonas aeruginosa* | na | Complete Genome |
| GCF_001722005.2 | *Pseudomonas aeruginosa* | strain=DN1 | Complete Genome |
| GCF_001515915.2 | *Pseudomonas aeruginosa* | strain=T38079 | Complete Genome |
| GCF_001516245.2 | *Pseudomonas aeruginosa* | strain=F23197 | Complete Genome |
| GCF_001542795.2 | *Pseudomonas aeruginosa* | strain=X78812 | Complete Genome |
| GCF_001516305.2 | *Pseudomonas aeruginosa* | strain=H5708 | Complete Genome |
| GCF_001516165.2 | *Pseudomonas aeruginosa* | strain=W16407 | Complete Genome |
| GCF_002192475.1 | *Pseudomonas aeruginosa* | strain=Pa124 | Complete Genome |
| GCF_002075065.1 | *Pseudomonas aeruginosa* | strain=PA_154197 | Complete Genome |
| GCF_001515845.2 | *Pseudomonas aeruginosa* | strain=S86968 | Complete Genome |
| GCF_001594325.2 | *Pseudomonas aeruginosa* | strain=F63912 | Complete Genome |
| GCF_001516225.2 | *Pseudomonas aeruginosa* | strain=W60856 | Complete Genome |
| GCF_001516185.2 | *Pseudomonas aeruginosa* | strain=W36662 | Complete Genome |
| GCF_001516325.2 | *Pseudomonas aeruginosa* | strain=H27930 | Complete Genome |
| GCF_001516205.2 | *Pseudomonas aeruginosa* | strain=W45909 | Complete Genome |
| GCF_002215345.1 | *Pseudomonas aeruginosa* | strain=PA83 | Complete Genome |
| GCF_002085605.1 | *Pseudomonas aeruginosa* | strain=RIVM-EMC2982 | Complete Genome |
| GCF_002085755.1 | *Pseudomonas aeruginosa* | strain=E6130952 | Complete Genome |
| GCF_002104595.1 | *Pseudomonas aeruginosa* | strain=PASGNDM699 | Complete Genome |
| GCF_002104615.1 | *Pseudomonas aeruginosa* | strain=PASGNDM345 | Complete Genome |
| GCF_002192495.1 | *Pseudomonas aeruginosa* | strain=Pa58 | Complete Genome |
| GCF_002205375.1 | *Pseudomonas aeruginosa* | strain=Pa1242 | Complete Genome |
| GCF_002205335.1 | *Pseudomonas aeruginosa* | strain=Pa84 | Complete Genome |
| GCF_002208645.1 | *Pseudomonas aeruginosa* | strain=Pa1207 | Complete Genome |
| GCF_002205355.1 | *Pseudomonas aeruginosa* | strain=Pa127 | Complete Genome |
| GCF_002223805.1 | *Pseudomonas aeruginosa* | strain=L10 | Complete Genome |
| GCF_002237405.1 | *Pseudomonas aeruginosa* | strain=Ocean-1155 | Complete Genome |
| GCF_002237425.1 | *Pseudomonas aeruginosa* | strain=Ocean-1175 | Complete Genome |
| GCF_002442855.1 | *Pseudomonas aeruginosa* | strain=PA_150577 | Complete Genome |
| GCF_002753655.1 | *Pseudomonas aeruginosa* | strain=12939 | Complete Genome |
| GCF_002812825.1 | *Pseudomonas aeruginosa* | strain=PB369 | Complete Genome |
| GCF_002812845.1 | *Pseudomonas aeruginosa* | strain=PB368 | Complete Genome |
| GCF_002812885.1 | *Pseudomonas aeruginosa* | strain=PB354 | Complete Genome |
| GCF_002812865.1 | *Pseudomonas aeruginosa* | strain=PB353 | Complete Genome |
| GCF_002968695.1 | *Pseudomonas aeruginosa* | strain=AR_0230 | Complete Genome |
| GCF_002968955.1 | *Pseudomonas aeruginosa* | strain=AR_0357 | Complete Genome |
| GCF_002968515.1 | *Pseudomonas aeruginosa* | strain=AR_0360 | Complete Genome |
| GCF_002968585.1 | *Pseudomonas aeruginosa* | strain=AR_0354 | Complete Genome |
| GCF_002946935.1 | *Pseudomonas aeruginosa* | strain=F5677 | Complete Genome |
| GCF_003073615.1 | *Pseudomonas aeruginosa* | strain=AR444 | Complete Genome |
| GCF_003028335.1 | *Pseudomonas aeruginosa* | strain=MRSN12280 | Complete Genome |
| GCF_002997005.1 | *Pseudomonas aeruginosa* | strain=AR_0095 | Complete Genome |
| GCF_002968655.1 | *Pseudomonas aeruginosa* | strain=AR_0353 | Complete Genome |
| GCF_003060845.1 | *Pseudomonas aeruginosa* | strain=JB2 | Complete Genome |
| GCF_003073795.1 | *Pseudomonas aeruginosa* | strain=AR442 | Complete Genome |
| GCF_003073895.1 | *Pseudomonas aeruginosa* | strain=AR439 | Complete Genome |
| GCF_002287725.2 | *Pseudomonas aeruginosa* | strain=PPF-1 | Complete Genome |
| GCF_003073735.1 | *Pseudomonas aeruginosa* | strain=AR445 | Complete Genome |
| GCF_003193645.1 | *Pseudomonas aeruginosa* | strain=AR_0446 | Complete Genome |
| GCF_003206535.1 | *Pseudomonas aeruginosa* | strain=K34-7 | Complete Genome |
| GCF_003288435.1 | *Pseudomonas aeruginosa* | strain=AR_458 | Complete Genome |
| GCF_003319235.1 | *Pseudomonas aeruginosa* | strain=HS9 | Complete Genome |
| GCF_003204335.1 | *Pseudomonas aeruginosa* | strain=AR_0110 | Complete Genome |
| GCF_003194245.1 | *Pseudomonas aeruginosa* | strain=CCUG 70744 | Complete Genome |
| GCF_003288335.1 | *Pseudomonas aeruginosa* | strain=AR_460 | Complete Genome |
| GCF_003369735.1 | *Pseudomonas aeruginosa* | strain=Y89 | Complete Genome |
| GCF_003369775.1 | *Pseudomonas aeruginosa* | strain=Y31 | Complete Genome |
| GCF_003429205.1 | *Pseudomonas aeruginosa* | strain=PABL017 | Complete Genome |
| GCF_003429185.1 | *Pseudomonas aeruginosa* | strain=PABL012 | Complete Genome |
| GCF_003408495.1 | *Pseudomonas aeruginosa* | strain=Y71 | Complete Genome |
| GCF_003369755.1 | *Pseudomonas aeruginosa* | strain=Y82 | Complete Genome |
| GCF_003433235.1 | *Pseudomonas aeruginosa* | strain=24Pae112 | Complete Genome |
| GCF_003571805.1 | *Pseudomonas aeruginosa* | strain=AR_0111 | Complete Genome |
| GCF_003641125.1 | *Pseudomonas aeruginosa* | strain=268 | Complete Genome |
| GCF_003332705.2 | *Pseudomonas aeruginosa* | strain=PA34 | Complete Genome |
| GCF_003571505.1 | *Pseudomonas aeruginosa* | strain=N15-01092 | Complete Genome |
| GCF_003716765.1 | *Pseudomonas aeruginosa* | strain=SP4528 | Complete Genome |
| GCF_003052005.2 | *Pseudomonas aeruginosa* | strain=WCHPA075019 | Complete Genome |
| GCF_003812905.1 | *Pseudomonas aeruginosa* | strain=FDAARGOS_501 | Complete Genome |
| GCF_003798145.1 | *Pseudomonas aeruginosa* | strain=H26023 | Complete Genome |
| GCF_003798105.1 | *Pseudomonas aeruginosa* | strain=H26027 | Complete Genome |
| GCF_900618265.1 | *Pseudomonas aeruginosa* | na | Complete Genome |
| GCF_900618285.1 | *Pseudomonas aeruginosa* | na | Complete Genome |
| GCF_900618245.1 | *Pseudomonas aeruginosa* | na | Complete Genome |
| GCF_003798125.1 | *Pseudomonas aeruginosa* | strain=H25883 | Complete Genome |
| GCF_900637045.1 | *Pseudomonas aeruginosa* | strain=NCTC10728 | Complete Genome |
| GCF_003950015.1 | *Pseudomonas aeruginosa* | strain=IMP-13 | Complete Genome |
| GCF_003812165.1 | *Pseudomonas aeruginosa* | strain=FDAARGOS_532 | Complete Genome |
| GCF_003813005.1 | *Pseudomonas aeruginosa* | strain=FDAARGOS_505 | Complete Genome |
| GCF_900618275.1 | *Pseudomonas aeruginosa* | na | Complete Genome |
| GCF_900618325.1 | *Pseudomonas aeruginosa* | na | Complete Genome |
| GCF_900618255.1 | *Pseudomonas aeruginosa* | na | Complete Genome |
| GCF_900636735.1 | *Pseudomonas aeruginosa* | strain=NCTC11445 | Complete Genome |
| GCF_003812885.1 | *Pseudomonas aeruginosa* | strain=FDAARGOS_571 | Complete Genome |
| GCF_003813025.1 | *Pseudomonas aeruginosa* | strain=FDAARGOS_570 | Complete Genome |
| GCF_003957825.1 | *Pseudomonas aeruginosa* | strain=GIMC5015:PAKB6 | Complete Genome |
| GCF_900618305.1 | *Pseudomonas aeruginosa* | na | Complete Genome |
| GCF_900618335.1 | *Pseudomonas aeruginosa* | na | Complete Genome |
| GCF_004010895.1 | *Pseudomonas aeruginosa* | strain=LW | Complete Genome |
| GCF_900636755.1 | *Pseudomonas aeruginosa* | strain=NCTC12903 | Complete Genome |
| GCF_900636975.1 | *Pseudomonas aeruginosa* | strain=NCTC13715 | Complete Genome |
| GCF_900618315.1 | *Pseudomonas aeruginosa* | na | Complete Genome |
| GCF_004102665.1 | *Pseudomonas aeruginosa* | strain=PAO1161 | Complete Genome |
| GCF_004193735.1 | *Pseudomonas aeruginosa* | strain=1334/14 | Complete Genome |
| GCF_900497025.1 | *Pseudomonas aeruginosa* | na | Complete Genome |
| GCF_004291075.1 | *Pseudomonas aeruginosa* | strain=E80 | Complete Genome |
| GCF_004355125.1 | *Pseudomonas aeruginosa* | strain=AES1M | Complete Genome |
| GCF_004355145.1 | *Pseudomonas aeruginosa* | strain=AES1R | Complete Genome |
| GCF_003411785.2 | *Pseudomonas aeruginosa* | strain=PABL048 | Complete Genome |
| GCF_005305005.1 | *Pseudomonas aeruginosa* | strain=PA298 | Complete Genome |
| GCF_002411865.3 | *Pseudomonas aeruginosa* | strain=97 | Complete Genome |
| GCF_006364735.1 | *Pseudomonas aeruginosa* | strain=FDAARGOS_767 | Complete Genome |
| GCF_006364795.1 | *Pseudomonas aeruginosa* | strain=FDAARGOS_610 | Complete Genome |
| GCF_003626935.1 | *Pseudomonas aeruginosa* | strain=BA7823 | Complete Genome |
| GCF_006971785.1 | *Pseudomonas aeruginosa* | strain=AZPAE15042 | Complete Genome |
| GCF_007559125.1 | *Pseudomonas aeruginosa* | strain=243931 | Complete Genome |
| GCF_007559065.1 | *Pseudomonas aeruginosa* | strain=60503 | Complete Genome |
| GCF_007559085.1 | *Pseudomonas aeruginosa* | strain=A681 | Complete Genome |
| GCF_007559105.1 | *Pseudomonas aeruginosa* | strain=519119 | Complete Genome |
| GCF_007833895.1 | *Pseudomonas aeruginosa* | strain=C79 | Complete Genome |
| GCF_008033745.1 | *Pseudomonas aeruginosa* | strain=IMP68 | Complete Genome |
| GCF_008033765.1 | *Pseudomonas aeruginosa* | strain=IMP66 | Complete Genome |
| GCF_008033725.1 | *Pseudomonas aeruginosa* | strain=IMP67 | Complete Genome |
| GCF_008245185.1 | *Pseudomonas aeruginosa* | strain=HOU1 | Complete Genome |
| GCF_008195485.1 | *Pseudomonas aeruginosa* | strain=CCUG 51971 | Complete Genome |
| GCF_008705235.1 | *Pseudomonas aeruginosa* | strain=E90 | Complete Genome |
| GCF_009497675.1 | *Pseudomonas aeruginosa* | strain=PA59 | Complete Genome |
| GCF_009662315.1 | *Pseudomonas aeruginosa* | strain=AG1 | Complete Genome |
| GCF_009664165.1 | *Pseudomonas aeruginosa* | strain=ST773 | Complete Genome |
| GCF_009648875.1 | *Pseudomonas aeruginosa* | strain=CFSAN084950 | Complete Genome |
| GCF_009676785.1 | *Pseudomonas aeruginosa* | strain=1811-18R001 | Complete Genome |
| GCF_009676885.1 | *Pseudomonas aeruginosa* | strain=KRP1 | Complete Genome |
| GCF_009720425.1 | *Pseudomonas aeruginosa* | strain=T2101 | Complete Genome |
| GCF_009720405.1 | *Pseudomonas aeruginosa* | strain=T2436 | Complete Genome |
| GCF_009676765.1 | *Pseudomonas aeruginosa* | strain=1811-13R031 | Complete Genome |
| GCF_009905195.1 | *Pseudomonas aeruginosa* | strain=INP-43 | Complete Genome |
| GCF_009867055.1 | *Pseudomonas aeruginosa* | strain=YB01 | Complete Genome |
| GCF_009911735.1 | *Pseudomonas aeruginosa* | strain=RD1-3 | Complete Genome |
| GCF_902703185.1 | *Pseudomonas aeruginosa* | strain=PcyII-29 | Complete Genome |
| GCF_902703195.1 | *Pseudomonas aeruginosa* | strain=PcyII-40 | Complete Genome |
| GCF_902703215.1 | *Pseudomonas aeruginosa* | strain=C7-25 | Complete Genome |
| GCF_002812905.2 | *Pseudomonas aeruginosa* | strain=PB350 | Complete Genome |
| GCF_011106815.1 | *Pseudomonas aeruginosa* | strain=PAG5 | Complete Genome |
| GCF_011466835.1 | *Pseudomonas aeruginosa* | strain=CF39S | Complete Genome |
| GCF_012276675.1 | *Pseudomonas aeruginosa* | strain=CMC-115 | Complete Genome |
| GCF_012935295.1 | *Pseudomonas aeruginosa* | strain=AA2 | Complete Genome |
| GCF_012971705.1 | *Pseudomonas aeruginosa* | strain=LYT4 | Complete Genome |
| GCF_013305815.1 | *Pseudomonas aeruginosa* | strain=LIUYANG-C | Complete Genome |
| GCF_011045375.1 | *Pseudomonas aeruginosa* | strain=MS14403 | Complete Genome |
| GCF_013305765.1 | *Pseudomonas aeruginosa* | strain=LIUYANG-E | Complete Genome |
| GCF_013341295.1 | *Pseudomonas aeruginosa* | strain=PAAK088 | Complete Genome |
| GCF_013201095.1 | *Pseudomonas aeruginosa* | strain=PAC6 | Complete Genome |
| GCF_013305845.1 | *Pseudomonas aeruginosa* | strain=LIUYANG-A | Complete Genome |
| GCF_013343535.1 | *Pseudomonas aeruginosa* | strain=DVT412 | Complete Genome |
| GCF_013343455.1 | *Pseudomonas aeruginosa* | strain=DVT417 | Complete Genome |
| GCF_013201115.1 | *Pseudomonas aeruginosa* | strain=PAC1 | Complete Genome |
| GCF_013343435.1 | *Pseudomonas aeruginosa* | strain=DVT419 | Complete Genome |
| GCF_013343415.1 | *Pseudomonas aeruginosa* | strain=DVT421 | Complete Genome |
| GCF_013260445.1 | *Pseudomonas aeruginosa* | strain=YD001 | Complete Genome |
| GCF_013255565.1 | *Pseudomonas aeruginosa* | strain=PSE6684 | Complete Genome |
| GCF_013343575.1 | *Pseudomonas aeruginosa* | strain=DVT401 | Complete Genome |
| GCF_013343555.1 | *Pseudomonas aeruginosa* | strain=DVT410 | Complete Genome |
| GCF_000763245.3 | *Pseudomonas aeruginosa* | strain=CCBH4851 | Complete Genome |
| GCF_013343475.1 | *Pseudomonas aeruginosa* | strain=DVT779 | Complete Genome |
| GCF_013347245.1 | *Pseudomonas aeruginosa* | strain=CDN118 | Complete Genome |
| GCF_013343315.1 | *Pseudomonas aeruginosa* | strain=DVT729 | Complete Genome |
| GCF_013343395.1 | *Pseudomonas aeruginosa* | strain=DVT423 | Complete Genome |
| GCF_013343355.1 | *Pseudomonas aeruginosa* | strain=DVT427 | Complete Genome |
| GCF_013343375.1 | *Pseudomonas aeruginosa* | strain=DVT425 | Complete Genome |
| GCF_013343335.1 | *Pseudomonas aeruginosa* | strain=DVT429 | Complete Genome |
| GCF_013395035.1 | *Pseudomonas aeruginosa* | strain=B18 | Complete Genome |
| GCF_013393685.1 | *Pseudomonas aeruginosa* | strain=SE5369 | Complete Genome |
| GCF_013343495.1 | *Pseudomonas aeruginosa* | strain=DVT414 | Complete Genome |
| GCF_013343515.1 | *Pseudomonas aeruginosa* | strain=DVT413 | Complete Genome |
| GCF_013376775.1 | *Pseudomonas aeruginosa* | strain=CDN129 | Complete Genome |
| GCF_004014755.1 | *Pseudomonas aeruginosa* | strain=PA0750 | Complete Genome |
| GCF_013393705.1 | *Pseudomonas aeruginosa* | strain=SE5416 | Complete Genome |
| GCF_013394455.1 | *Pseudomonas aeruginosa* | strain=SE5443 | Complete Genome |
| GCF_013467605.1 | *Pseudomonas aeruginosa* | strain=ACR22 | Complete Genome |
| GCF_013467585.1 | *Pseudomonas aeruginosa* | strain=ACR20 | Complete Genome |
| GCF_014217315.1 | *Pseudomonas aeruginosa* | strain=JNQH-PA57 | Complete Genome |
| GCF_014155905.1 | *Pseudomonas aeruginosa* | strain=NY3045 | Complete Genome |
| GCF_014467035.1 | *Pseudomonas aeruginosa* | strain=NRD619 | Complete Genome |
| GCF_014490645.1 | *Pseudomonas aeruginosa* | strain=PA3 | Complete Genome |
| GCF_014792125.1 | *Pseudomonas aeruginosa* | strain=R31 | Complete Genome |
| GCF_014854655.1 | *Pseudomonas aeruginosa* | strain=JT86 | Complete Genome |
| GCF_015697605.1 | *Pseudomonas aeruginosa* | strain=C-I-1 | Complete Genome |
| GCF_015697645.1 | *Pseudomonas aeruginosa* | strain=B-I-1 | Complete Genome |
| GCF_015697665.1 | *Pseudomonas aeruginosa* | strain=A-I-1 | Complete Genome |
| GCF_015832055.1 | *Pseudomonas aeruginosa* | na | Complete Genome |
| GCF_015697465.1 | *Pseudomonas aeruginosa* | strain=G-I-1 | Complete Genome |
| GCF_014930935.1 | *Pseudomonas aeruginosa* | strain=KC-Tt-1 | Complete Genome |
| GCF_015832075.1 | *Pseudomonas aeruginosa* | na | Complete Genome |
| GCF_016105665.1 | *Pseudomonas aeruginosa* | strain=TJ2019-017 | Complete Genome |
| GCF_016107485.1 | *Pseudomonas aeruginosa* | strain=MPAO1 | Complete Genome |
| GCF_016126955.1 | *Pseudomonas aeruginosa* | strain=FDAARGOS_1041 | Complete Genome |
| GCF_016064595.1 | *Pseudomonas aeruginosa* | strain=CMC-097 | Complete Genome |
| GCF_016105505.1 | *Pseudomonas aeruginosa* | strain=TJ2019-022 | Complete Genome |
| GCF_016105885.1 | *Pseudomonas aeruginosa* | strain=TJ2014-049 | Complete Genome |
| GCF_016134915.1 | *Pseudomonas aeruginosa* | strain=DL201330 | Complete Genome |
| GCF_013393665.2 | *Pseudomonas aeruginosa* | strain=SE5331 | Complete Genome |
| GCF_013305645.2 | *Pseudomonas aeruginosa* | strain=YT12746 | Complete Genome |
| GCF_016743115.1 | *Pseudomonas aeruginosa* | strain=PA19-3047 | Complete Genome |
| GCF_016743035.1 | *Pseudomonas aeruginosa* | strain=A39-1 | Complete Genome |
| GCF_016584725.1 | *Pseudomonas aeruginosa* | strain=LYSZa7 | Complete Genome |
| GCF_013394475.2 | *Pseudomonas aeruginosa* | strain=SE5458 | Complete Genome |
| GCF_016745155.1 | *Pseudomonas aeruginosa* | strain=SE5357 | Complete Genome |
| GCF_016745135.1 | *Pseudomonas aeruginosa* | strain=SE5352 | Complete Genome |
| GCF_002812925.2 | *Pseudomonas aeruginosa* | strain=PB367 | Complete Genome |
| GCF_904866275.1 | *Pseudomonas aeruginosa* | strain=MINF_7A | Complete Genome |
| GCF_016745175.1 | *Pseudomonas aeruginosa* | strain=SE5429 | Complete Genome |
| GCF_016811975.1 | *Pseudomonas aeruginosa* | strain=152962 | Complete Genome |
| GCF_016745115.1 | *Pseudomonas aeruginosa* | strain=YTSEY8 | Complete Genome |
| GCF_016745195.1 | *Pseudomonas aeruginosa* | strain=1903031130 | Complete Genome |
| GCF_905071885.1 | *Pseudomonas aeruginosa* | strain=MINF_3A | Complete Genome |
| GCF_016864415.1 | *Pseudomonas aeruginosa* | strain=PARM801 | Complete Genome |
| GCF_016925475.1 | *Pseudomonas aeruginosa* | strain=B17932 | Complete Genome |
| GCF_016925455.1 | *Pseudomonas aeruginosa* | strain=B17416 | Complete Genome |
| GCF_017378475.1 | *Pseudomonas aeruginosa* | strain=LYSZa2 | Complete Genome |
| GCF_017378395.1 | *Pseudomonas aeruginosa* | strain=LYSZa5 | Complete Genome |
| GCF_017900915.1 | *Pseudomonas aeruginosa* | strain=LICME WGH-6 | Complete Genome |
| GCF_018138045.1 | *Pseudomonas aeruginosa* | strain=NDTH9845 | Complete Genome |
| GCF_018279285.1 | *Pseudomonas aeruginosa* | strain=ZBX-P12 | Complete Genome |
| GCF_018141625.1 | *Pseudomonas aeruginosa* | strain=PAS6 | Complete Genome |
| GCF_018279305.1 | *Pseudomonas aeruginosa* | strain=ZBX-P11 | Complete Genome |
| GCF_018279265.1 | *Pseudomonas aeruginosa* | strain=ZBX-P13 | Complete Genome |
| GCF_018138065.1 | *Pseudomonas aeruginosa* | strain=WTJH17 | Complete Genome |
| GCF_018279245.1 | *Pseudomonas aeruginosa* | strain=ZBX-P23 | Complete Genome |
| GCF_018409365.1 | *Pseudomonas aeruginosa* | na | Complete Genome |
| GCF_018448985.1 | *Pseudomonas aeruginosa* | strain=PA790 | Complete Genome |
| GCF_018141645.1 | *Pseudomonas aeruginosa* | strain=PAM68 | Complete Genome |
| GCF_018141565.1 | *Pseudomonas aeruginosa* | strain=PAG7 | Complete Genome |
| GCF_019434095.1 | *Pseudomonas aeruginosa* | strain=UNC_PaerCF17 | Complete Genome |
| GCF_019434075.1 | *Pseudomonas aeruginosa* | strain=UNC_PaerCF20 | Complete Genome |
| GCF_019378915.1 | *Pseudomonas aeruginosa* | strain=S-1 | Complete Genome |
| GCF_019379355.1 | *Pseudomonas aeruginosa* | strain=TL3773 | Complete Genome |
| GCF_019364615.1 | *Pseudomonas aeruginosa* | strain=HS17-127 | Complete Genome |
| GCF_019443665.1 | *Pseudomonas aeruginosa* | strain=2020HL-00861 | Complete Genome |
| GCF_019434235.1 | *Pseudomonas aeruginosa* | strain=PA2207 | Complete Genome |
| GCF_019434135.1 | *Pseudomonas aeruginosa* | strain=UNC_PaerCF12 | Complete Genome |
| GCF_019466145.1 | *Pseudomonas aeruginosa* | strain=YY322 | Complete Genome |
| GCF_019710495.1 | *Pseudomonas aeruginosa* | strain=P9W | Complete Genome |
| GCF_019466095.1 | *Pseudomonas aeruginosa* | strain=DJ06 | Complete Genome |
| GCF_019711215.1 | *Pseudomonas aeruginosa* | strain=F092021 | Complete Genome |
| GCF_019720875.1 | *Pseudomonas aeruginosa* | strain=F291007 | Complete Genome |
| GCF_019720855.1 | *Pseudomonas aeruginosa* | strain=SE5419 | Complete Genome |
| GCF_019857485.1 | *Pseudomonas aeruginosa* | strain=ZPPH1 | Complete Genome |
| GCF_019857605.1 | *Pseudomonas aeruginosa* | strain=NDTH7329 | Complete Genome |
| GCF_019857525.1 | *Pseudomonas aeruginosa* | strain=SRRSH1521 | Complete Genome |
| GCF_019857345.1 | *Pseudomonas aeruginosa* | strain=NDTH10366 | Complete Genome |
| GCF_019857305.1 | *Pseudomonas aeruginosa* | strain=SRRSH1408 | Complete Genome |
| GCF_019857405.1 | *Pseudomonas aeruginosa* | strain=ZPPH33 | Complete Genome |
| GCF_019857445.1 | *Pseudomonas aeruginosa* | strain=ZPPH14 | Complete Genome |
| GCF_019915465.1 | *Pseudomonas aeruginosa* | strain=SCAID TST-2021 (7/157) | Complete Genome |
| GCF_019915485.1 | *Pseudomonas aeruginosa* | strain=SCAID PLC1-2021 (16/222) | Complete Genome |
| GCF_019857585.1 | *Pseudomonas aeruginosa* | strain=QZPH16 | Complete Genome |
| GCF_019857365.1 | *Pseudomonas aeruginosa* | strain=WTJH12 | Complete Genome |
| GCF_019857465.1 | *Pseudomonas aeruginosa* | strain=ZPPH2 | Complete Genome |
| GCF_019857285.1 | *Pseudomonas aeruginosa* | strain=SRRSH1002 | Complete Genome |
| GCF_019857425.1 | *Pseudomonas aeruginosa* | strain=ZPPH29 | Complete Genome |
| GCF_019857325.1 | *Pseudomonas aeruginosa* | strain=QZPH41 | Complete Genome |
| GCF_019857245.1 | *Pseudomonas aeruginosa* | strain=SRRSH15 | Complete Genome |
| GCF_019915445.1 | *Pseudomonas aeruginosa* | strain=SCAID WND1-2021 (9/195) | Complete Genome |
| GCF_019857565.1 | *Pseudomonas aeruginosa* | strain=QZPH21 | Complete Genome |
| GCF_019857265.1 | *Pseudomonas aeruginosa* | strain=SRRSH1101 | Complete Genome |
| GCF_019857505.1 | *Pseudomonas aeruginosa* | strain=SRRSH2790 | Complete Genome |
| GCF_019857625.1 | *Pseudomonas aeruginosa* | strain=FAHZU40 | Complete Genome |
| GCF_019857645.1 | *Pseudomonas aeruginosa* | strain=FAHZU31 | Complete Genome |
| GCF_019857545.1 | *Pseudomonas aeruginosa* | strain=SRRSH1120 | Complete Genome |
| GCF_006704595.2 | *Pseudomonas aeruginosa* | strain=PS1793 | Complete Genome |
| GCF_020771755.1 | *Pseudomonas aeruginosa* | na | Complete Genome |
| GCF_018598285.3 | *Pseudomonas aeruginosa* | strain=CCBH28525 | Complete Genome |
| GCF_020990485.1 | *Pseudomonas aeruginosa* | strain=P93127 | Complete Genome |
| GCF_020771675.1 | *Pseudomonas aeruginosa* | na | Complete Genome |
| GCF_020990465.1 | *Pseudomonas aeruginosa* | strain=P4970C | Complete Genome |
| GCF_020990445.1 | *Pseudomonas aeruginosa* | strain=P96131 | Complete Genome |
| GCF_021266605.1 | *Pseudomonas aeruginosa* | strain=PA0523 | Complete Genome |
| GCF_021249245.1 | *Pseudomonas aeruginosa* | strain=Pa608 | Complete Genome |
| GCF_021378395.1 | *Pseudomonas aeruginosa* | strain=Pae1255-NDM1 | Complete Genome |
| GCF_021497405.1 | *Pseudomonas aeruginosa* | strain=SE5431 | Complete Genome |
| GCF_021513375.1 | *Pseudomonas aeruginosa* | strain=R06 | Complete Genome |
| GCF_021497305.1 | *Pseudomonas aeruginosa* | strain=HB2011305RE | Complete Genome |
| GCF_021497425.1 | *Pseudomonas aeruginosa* | strain=SE5452 | Complete Genome |
| GCF_021513535.1 | *Pseudomonas aeruginosa* | strain=R09 | Complete Genome |
| GCF_021513595.1 | *Pseudomonas aeruginosa* | strain=E03 | Complete Genome |
| GCF_021513575.1 | *Pseudomonas aeruginosa* | strain=R01 | Complete Genome |
| GCF_021460035.1 | *Pseudomonas aeruginosa* | strain=MIN-155 | Complete Genome |
| GCF_021513415.1 | *Pseudomonas aeruginosa* | strain=R02 | Complete Genome |
| GCF_021513295.1 | *Pseudomonas aeruginosa* | strain=NCCP15783 | Complete Genome |
| GCF_021513495.1 | *Pseudomonas aeruginosa* | strain=R10 | Complete Genome |
| GCF_021513475.1 | *Pseudomonas aeruginosa* | strain=R03 | Complete Genome |
| GCF_021513675.1 | *Pseudomonas aeruginosa* | strain=E04 | Complete Genome |
| GCF_021184265.1 | *Pseudomonas aeruginosa* | strain=JNQH-PA033 | Complete Genome |
| GCF_021513615.1 | *Pseudomonas aeruginosa* | strain=E02 | Complete Genome |
| GCF_021513515.1 | *Pseudomonas aeruginosa* | strain=R07 | Complete Genome |
| GCF_021184245.1 | *Pseudomonas aeruginosa* | strain=JNQH-PA027 | Complete Genome |
| GCF_021497445.1 | *Pseudomonas aeruginosa* | strain=A0002 | Complete Genome |
| GCF_021497385.1 | *Pseudomonas aeruginosa* | strain=SE5430 | Complete Genome |
| GCF_021398755.1 | *Pseudomonas aeruginosa* | strain=PA8329 | Complete Genome |
| GCF_021513635.1 | *Pseudomonas aeruginosa* | strain=E01 | Complete Genome |
| GCF_021513555.1 | *Pseudomonas aeruginosa* | strain=R08 | Complete Genome |
| GCF_021513455.1 | *Pseudomonas aeruginosa* | strain=R04 | Complete Genome |
| GCF_021513435.1 | *Pseudomonas aeruginosa* | strain=R11 | Complete Genome |
| GCF_021497285.1 | *Pseudomonas aeruginosa* | strain=DL201330 | Complete Genome |
| GCF_021513395.1 | *Pseudomonas aeruginosa* | strain=R05 | Complete Genome |
| GCF_021497325.1 | *Pseudomonas aeruginosa* | strain=YTSY4 | Complete Genome |
| GCF_022569855.1 | *Pseudomonas aeruginosa* | strain=H20 | Complete Genome |
| GCF_022569895.1 | *Pseudomonas aeruginosa* | strain=H17 | Complete Genome |
| GCF_022559545.1 | *Pseudomonas aeruginosa* | strain=PES_P749 | Complete Genome |
| GCF_022569915.1 | *Pseudomonas aeruginosa* | strain=H16 | Complete Genome |
| GCF_022570115.1 | *Pseudomonas aeruginosa* | strain=H08 | Complete Genome |
| GCF_022453765.1 | *Pseudomonas aeruginosa* | strain=HU20 | Complete Genome |
| GCF_022569975.1 | *Pseudomonas aeruginosa* | strain=H10 | Complete Genome |
| GCF_022569935.1 | *Pseudomonas aeruginosa* | strain=H15 | Complete Genome |
| GCF_022570435.1 | *Pseudomonas aeruginosa* | strain=H04 | Complete Genome |
| GCF_022453825.1 | *Pseudomonas aeruginosa* | strain=LS.2c | Complete Genome |
| GCF_022570415.1 | *Pseudomonas aeruginosa* | strain=H05 | Complete Genome |
| GCF_022569995.1 | *Pseudomonas aeruginosa* | strain=H09 | Complete Genome |
| GCF_022570235.1 | *Pseudomonas aeruginosa* | strain=H07 | Complete Genome |
| GCF_022570455.1 | *Pseudomonas aeruginosa* | strain=H03 | Complete Genome |
| GCF_022638175.1 | *Pseudomonas aeruginosa* | strain=E131 | Complete Genome |
| GCF_022638055.1 | *Pseudomonas aeruginosa* | strain=E104 | Complete Genome |
| GCF_021166255.2 | *Pseudomonas aeruginosa* | strain=UNC_PaerCF41 | Complete Genome |
| GCF_021166295.1 | *Pseudomonas aeruginosa* | strain=UNC_PaerCF37 | Complete Genome |
| GCF_022810965.1 | *Pseudomonas aeruginosa* | strain=ST1076_d97burn1 | Complete Genome |
| GCF_022810905.1 | *Pseudomonas aeruginosa* | strain=ST1076_d100blood1 | Complete Genome |
| GCF_021166335.2 | *Pseudomonas aeruginosa* | strain=UNC_PaerCF34 | Complete Genome |
| GCF_021166375.2 | *Pseudomonas aeruginosa* | strain=UNC_PaerCF13 | Complete Genome |
| GCF_021166355.2 | *Pseudomonas aeruginosa* | strain=UNC_PaerCF19 | Complete Genome |
| GCF_021166315.2 | *Pseudomonas aeruginosa* | strain=UNC_PaerCF35 | Complete Genome |
| GCF_022637915.1 | *Pseudomonas aeruginosa* | strain=E113 | Complete Genome |
| GCF_022811025.1 | *Pseudomonas aeruginosa* | strain=ST167_d67burn2 | Complete Genome |
| GCF_022811045.1 | *Pseudomonas aeruginosa* | strain=ST167_d26burn | Complete Genome |
| GCF_022810925.1 | *Pseudomonas aeruginosa* | strain=ST1076_d97burn2 | Complete Genome |
| GCF_022810865.1 | *Pseudomonas aeruginosa* | strain=St1076_d123blood | Complete Genome |
| GCF_022811005.1 | *Pseudomonas aeruginosa* | strain=ST167_d68blood1 | Complete Genome |
| GCF_022570475.1 | *Pseudomonas aeruginosa* | strain=H02 | Complete Genome |
| GCF_019738995.2 | *Pseudomonas aeruginosa* | strain=P8W | Complete Genome |
| GCF_022649105.1 | *Pseudomonas aeruginosa* | strain=E125 | Complete Genome |
| GCF_022670795.1 | *Pseudomonas aeruginosa* | strain=PA1_NCHU | Complete Genome |
| GCF_022699485.1 | *Pseudomonas aeruginosa* | strain=ATCC BAA-2114 | Complete Genome |
| GCF_022811805.1 | *Pseudomonas aeruginosa* | strain=ST1076_d100blood2 | Complete Genome |
| GCF_022811065.1 | *Pseudomonas aeruginosa* | strain=ST167_d57blood | Complete Genome |
| GCF_021166275.2 | *Pseudomonas aeruginosa* | strain=UNC_PaerCF38 | Complete Genome |
| GCF_022649245.1 | *Pseudomonas aeruginosa* | strain=E167 | Complete Genome |
| GCF_022699505.1 | *Pseudomonas aeruginosa* | strain=ATCC BAA-2108 | Complete Genome |
| GCF_022699525.1 | *Pseudomonas aeruginosa* | strain=NY4605 | Complete Genome |
| GCF_022810945.1 | *Pseudomonas aeruginosa* | strain=ST167_d68blood2 | Complete Genome |
| GCF_022810985.1 | *Pseudomonas aeruginosa* | strain=ST167_d67burn1 | Complete Genome |
| GCF_022810885.1 | *Pseudomonas aeruginosa* | strain=ST1076_d118limb1 | Complete Genome |
| GCF_023066845.1 | *Pseudomonas aeruginosa* | strain=PA1616 | Complete Genome |
| GCF_023101265.1 | *Pseudomonas aeruginosa* | strain=AR19640 | Complete Genome |
| GCF_022870565.1 | *Pseudomonas aeruginosa* | strain=CHA | Complete Genome |
| GCF_022870545.1 | *Pseudomonas aeruginosa* | strain=AA43 | Complete Genome |
| GCF_023093975.1 | *Pseudomonas aeruginosa* | strain=34Pae8 | Complete Genome |
| GCF_023093935.1 | *Pseudomonas aeruginosa* | strain=34Pae36 | Complete Genome |
| GCF_022870525.1 | *Pseudomonas aeruginosa* | strain=A5803 | Complete Genome |
| GCF_022870025.1 | *Pseudomonas aeruginosa* | strain=PartH-Paeruginosa-RM8376 | Complete Genome |
| GCF_023066865.1 | *Pseudomonas aeruginosa* | strain=PA1609 | Complete Genome |
| GCF_023066825.1 | *Pseudomonas aeruginosa* | strain=PA1681 | Complete Genome |
| GCF_023101285.1 | *Pseudomonas aeruginosa* | strain=AR19583 | Complete Genome |
| GCF_023101305.1 | *Pseudomonas aeruginosa* | strain=AR19438 | Complete Genome |
| GCF_023272755.1 | *Pseudomonas aeruginosa* | strain=8D | Complete Genome |
| GCF_023380065.1 | *Pseudomonas aeruginosa* | strain=D5 | Complete Genome |
| GCF_023520735.1 | *Pseudomonas aeruginosa* | strain=L00-a | Complete Genome |
| GCF_023612335.1 | *Pseudomonas aeruginosa* | strain=Z154 | Complete Genome |
| GCF_023571485.1 | *Pseudomonas aeruginosa* | strain=UNC_PaerCF25 | Complete Genome |
| GCF_023822305.1 | *Pseudomonas aeruginosa* | strain=US449 | Complete Genome |
| GCF_024089395.1 | *Pseudomonas aeruginosa* | strain=PAO1_Mat-X-1 | Complete Genome |
| GCF_024126335.1 | *Pseudomonas aeruginosa* | strain=PAO1-L | Complete Genome |
| GCF_024126355.1 | *Pseudomonas aeruginosa* | strain=PAO1-N | Complete Genome |
| GCF_024300845.1 | *Pseudomonas aeruginosa* | strain=R20-14 | Complete Genome |
| GCF_024266805.1 | *Pseudomonas aeruginosa* | strain=F13 | Complete Genome |
| GCF_024397355.1 | *Pseudomonas aeruginosa* | strain=D-2 | Complete Genome |
| GCF_024266915.1 | *Pseudomonas aeruginosa* | strain=PA-1 | Complete Genome |
| GCF_024266935.1 | *Pseudomonas aeruginosa* | strain=PA-2 | Complete Genome |
| GCF_024507915.1 | *Pseudomonas aeruginosa* | strain=NWRC-1223 | Complete Genome |
| GCF_024507955.1 | *Pseudomonas aeruginosa* | strain=ATCC 27853 | Complete Genome |
| GCF_022213165.2 | *Pseudomonas aeruginosa* | strain=L1a | Complete Genome |
| GCF_024584495.1 | *Pseudomonas aeruginosa* | strain=PA5083 | Complete Genome |
| GCF_024734815.1 | *Pseudomonas aeruginosa* | strain=SCAID WND1-2022 (148) | Complete Genome |
| GCF_024803805.1 | *Pseudomonas aeruginosa* | strain=Pa150 | Complete Genome |
| GCF_024734795.1 | *Pseudomonas aeruginosa* | strain=SCAID TCT1-2022 (325) | Complete Genome |
| GCF_024760445.1 | *Pseudomonas aeruginosa* | strain=PLL01 | Complete Genome |
| GCF_024972855.1 | *Pseudomonas aeruginosa* | strain=M27432 | Complete Genome |
| GCF_025021745.1 | *Pseudomonas aeruginosa* | strain=PAO1-Holloway | Complete Genome |
| GCF_025021565.1 | *Pseudomonas aeruginosa* | strain=PAO1-UW | Complete Genome |
| GCF_025200845.1 | *Pseudomonas aeruginosa* | strain=HW001G | Complete Genome |
| GCF_025244965.1 | *Pseudomonas aeruginosa* | strain=PLL01 | Complete Genome |
| GCF_025263705.1 | *Pseudomonas aeruginosa* | strain=PA01135 | Complete Genome |
| GCF_025263605.1 | *Pseudomonas aeruginosa* | strain=PA0386 | Complete Genome |
| GCF_025263585.1 | *Pseudomonas aeruginosa* | strain=PA0200 | Complete Genome |
| GCF_025398135.1 | *Pseudomonas aeruginosa* | strain=WTJH36 | Complete Genome |
| GCF_025398835.1 | *Pseudomonas aeruginosa* | strain=NY4593 | Complete Genome |
| GCF_025398095.1 | *Pseudomonas aeruginosa* | strain=WTJH6 | Complete Genome |
| GCF_025398115.1 | *Pseudomonas aeruginosa* | strain=WTJH32 | Complete Genome |
| GCF_025452135.1 | *Pseudomonas aeruginosa* | strain=PA-AUTBAM | Complete Genome |
| GCF_025398075.1 | *Pseudomonas aeruginosa* | strain=WTJH2 | Complete Genome |
| GCF_025723085.1 | *Pseudomonas aeruginosa* | strain=Pa3 | Complete Genome |
| GCF_025908315.1 | *Pseudomonas aeruginosa* | strain=Zw26 | Complete Genome |
| GCF_025790885.1 | *Pseudomonas aeruginosa* | strain=2019CK-00034 | Complete Genome |
| GCF_025985465.1 | *Pseudomonas aeruginosa* | strain=Paer4 | Complete Genome |
| GCF_024652945.3 | *Pseudomonas aeruginosa* | strain=PA30 | Complete Genome |
| GCF_026727715.1 | *Pseudomonas aeruginosa* | strain=B1.2 | Complete Genome |
| GCF_026625905.1 | *Pseudomonas aeruginosa* | strain=BIAI 157 | Complete Genome |
| GCF_026636195.1 | *Pseudomonas aeruginosa* | strain=BIAI 160 | Complete Genome |
| GCF_026727735.1 | *Pseudomonas aeruginosa* | strain=C1.3 | Complete Genome |
| GCF_026727695.1 | *Pseudomonas aeruginosa* | strain=B2.1 | Complete Genome |
| GCF_026727755.1 | *Pseudomonas aeruginosa* | strain=C4.2 | Complete Genome |
| GCF_026636135.1 | *Pseudomonas aeruginosa* | strain=SMC4386 | Complete Genome |
| GCF_026870135.1 | *Pseudomonas aeruginosa* | strain=M6A146 | Complete Genome |
| GCF_027171385.1 | *Pseudomonas aeruginosa* | strain=Jade-X | Complete Genome |
| GCF_027570535.1 | *Pseudomonas aeruginosa* | strain=PALA11 | Complete Genome |
| GCF_027571035.1 | *Pseudomonas aeruginosa* | strain=PALA43 | Complete Genome |
| GCF_027570435.1 | *Pseudomonas aeruginosa* | strain=PALA7 | Complete Genome |
| GCF_027570855.1 | *Pseudomonas aeruginosa* | strain=PALA33 | Complete Genome |
| GCF_027571135.1 | *Pseudomonas aeruginosa* | strain=PALA44 | Complete Genome |
| GCF_027571365.1 | *Pseudomonas aeruginosa* | strain=PALA38 | Complete Genome |
| GCF_027570615.1 | *Pseudomonas aeruginosa* | strain=PALA15 | Complete Genome |
| GCF_027571345.1 | *Pseudomonas aeruginosa* | strain=PALA54 | Complete Genome |
| GCF_027570655.1 | *Pseudomonas aeruginosa* | strain=PALA17 | Complete Genome |
| GCF_027570875.1 | *Pseudomonas aeruginosa* | strain=PALA34 | Complete Genome |
| GCF_027571095.1 | *Pseudomonas aeruginosa* | strain=PALA35 | Complete Genome |
| GCF_027570595.1 | *Pseudomonas aeruginosa* | strain=PALA14 | Complete Genome |
| GCF_027570935.1 | *Pseudomonas aeruginosa* | strain=PALA51 | Complete Genome |
| GCF_027570835.1 | *Pseudomonas aeruginosa* | strain=PALA32 | Complete Genome |
| GCF_027570915.1 | *Pseudomonas aeruginosa* | strain=PALA53 | Complete Genome |
| GCF_027570415.1 | *Pseudomonas aeruginosa* | strain=PALA2 | Complete Genome |
| GCF_027570675.1 | *Pseudomonas aeruginosa* | strain=PALA19 | Complete Genome |
| GCF_027570975.1 | *Pseudomonas aeruginosa* | strain=PALA56 | Complete Genome |
| GCF_027570775.1 | *Pseudomonas aeruginosa* | strain=PALA25 | Complete Genome |
| GCF_027570555.1 | *Pseudomonas aeruginosa* | strain=PALA12 | Complete Genome |
| GCF_027570795.1 | *Pseudomonas aeruginosa* | strain=PALA26 | Complete Genome |
| GCF_027359235.1 | *Pseudomonas aeruginosa* | strain=NF143349 | Complete Genome |
| GCF_027570955.1 | *Pseudomonas aeruginosa* | strain=PALA55 | Complete Genome |
| GCF_027570635.1 | *Pseudomonas aeruginosa* | strain=PALA16 | Complete Genome |
| GCF_027571115.1 | *Pseudomonas aeruginosa* | strain=PALA36 | Complete Genome |
| GCF_027571015.1 | *Pseudomonas aeruginosa* | strain=PALA42 | Complete Genome |
| GCF_027570575.1 | *Pseudomonas aeruginosa* | strain=PALA13 | Complete Genome |
| GCF_027570475.1 | *Pseudomonas aeruginosa* | strain=PALA4 | Complete Genome |
| GCF_027570455.1 | *Pseudomonas aeruginosa* | strain=PALA9 | Complete Genome |
| GCF_027571075.1 | *Pseudomonas aeruginosa* | strain=PALA30 | Complete Genome |
| GCF_027571195.1 | *Pseudomonas aeruginosa* | strain=PALA47 | Complete Genome |
| GCF_027570495.1 | *Pseudomonas aeruginosa* | strain=PALA6 | Complete Genome |
| GCF_027571055.1 | *Pseudomonas aeruginosa* | strain=PALA24 | Complete Genome |
| GCF_027571285.1 | *Pseudomonas aeruginosa* | strain=PALA1 | Complete Genome |
| GCF_027571155.1 | *Pseudomonas aeruginosa* | strain=PALA40 | Complete Genome |
| GCF_027570715.1 | *Pseudomonas aeruginosa* | strain=PALA20 | Complete Genome |
| GCF_027570515.1 | *Pseudomonas aeruginosa* | strain=PALA8 | Complete Genome |
| GCF_027571175.1 | *Pseudomonas aeruginosa* | strain=PALA45 | Complete Genome |
| GCF_027571325.1 | *Pseudomonas aeruginosa* | strain=PALA50 | Complete Genome |
| GCF_027570735.1 | *Pseudomonas aeruginosa* | strain=PALA22 | Complete Genome |
| GCF_027570995.1 | *Pseudomonas aeruginosa* | strain=PALA39 | Complete Genome |
| GCF_027594965.1 | *Pseudomonas aeruginosa* | strain=NY8709 | Complete Genome |
| GCF_027595005.1 | *Pseudomonas aeruginosa* | strain=NY7770 | Complete Genome |
| GCF_027595025.1 | *Pseudomonas aeruginosa* | strain=NY7610 | Complete Genome |
| GCF_027594685.1 | *Pseudomonas aeruginosa* | strain=NY5085 | Complete Genome |
| GCF_027571215.1 | *Pseudomonas aeruginosa* | strain=PALA52 | Complete Genome |
| GCF_027570895.1 | *Pseudomonas aeruginosa* | strain=PALA37 | Complete Genome |
| GCF_027594985.1 | *Pseudomonas aeruginosa* | strain=NY8688 | Complete Genome |
| GCF_027571305.1 | *Pseudomonas aeruginosa* | strain=PALA48 | Complete Genome |
| GCF_027570755.1 | *Pseudomonas aeruginosa* | strain=PALA23 | Complete Genome |
| GCF_027570815.1 | *Pseudomonas aeruginosa* | strain=PALA29 | Complete Genome |
| GCF_028404085.1 | *Pseudomonas aeruginosa* | strain=2868 | Complete Genome |
| GCF_028404065.1 | *Pseudomonas aeruginosa* | strain=2866 | Complete Genome |
| GCF_028404005.1 | *Pseudomonas aeruginosa* | strain=2858 | Complete Genome |
| GCF_028403965.1 | *Pseudomonas aeruginosa* | strain=2856 | Complete Genome |
| GCF_028404025.1 | *Pseudomonas aeruginosa* | strain=2880 | Complete Genome |
| GCF_028403985.1 | *Pseudomonas aeruginosa* | strain=2867 | Complete Genome |
| GCF_028404045.1 | *Pseudomonas aeruginosa* | strain=2857 | Complete Genome |
| GCF_028404105.1 | *Pseudomonas aeruginosa* | strain=2881 | Complete Genome |
| GCF_028622895.1 | *Pseudomonas aeruginosa* | strain=2022CK-00828 | Complete Genome |
| GCF_028622015.1 | *Pseudomonas aeruginosa* | strain=MF1 | Complete Genome |
| GCF_028743595.1 | *Pseudomonas aeruginosa* | strain=B-3509 | Complete Genome |
| GCF_028751785.1 | *Pseudomonas aeruginosa* | strain=HS18-89 | Complete Genome |
| GCF_028961985.1 | *Pseudomonas aeruginosa* | strain=0201761-1 | Complete Genome |
| GCF_029201245.1 | *Pseudomonas aeruginosa* | strain=SNDPR-01 | Complete Genome |
| GCF_029857015.1 | *Pseudomonas aeruginosa* | strain=HS337 | Complete Genome |
| GCF_029916825.1 | *Pseudomonas aeruginosa* | strain=59 | Complete Genome |
| GCF_028595525.2 | *Pseudomonas aeruginosa* | strain=P23 | Complete Genome |
| GCF_029856995.1 | *Pseudomonas aeruginosa* | strain=HS204 | Complete Genome |
| GCF_029962185.1 | *Pseudomonas aeruginosa* | strain=2022CK-00160 | Complete Genome |
| GCF_029961425.1 | *Pseudomonas aeruginosa* | strain=2021CK-01283 | Complete Genome |
| GCF_029961405.1 | *Pseudomonas aeruginosa* | strain=2021CK-01229 | Complete Genome |
| GCF_029961505.1 | *Pseudomonas aeruginosa* | strain=2021CK-01494 | Complete Genome |
| GCF_029961605.1 | *Pseudomonas aeruginosa* | strain=2021CK-01851 | Complete Genome |
| GCF_029961325.1 | *Pseudomonas aeruginosa* | strain=2021CK-01162 | Complete Genome |
| GCF_029962205.1 | *Pseudomonas aeruginosa* | strain=2021CK-01633 | Complete Genome |
| GCF_029961305.1 | *Pseudomonas aeruginosa* | strain=2021CK-01158 | Complete Genome |
| GCF_029961485.1 | *Pseudomonas aeruginosa* | strain=2021CK-01536 | Complete Genome |
| GCF_029961245.1 | *Pseudomonas aeruginosa* | strain=2021CK-01159 | Complete Genome |
| GCF_029961625.1 | *Pseudomonas aeruginosa* | strain=2022CK-00069 | Complete Genome |
| GCF_029961585.1 | *Pseudomonas aeruginosa* | strain=2020CK-00217 | Complete Genome |
| GCF_029961445.1 | *Pseudomonas aeruginosa* | strain=2021CK-01315 | Complete Genome |
| GCF_029961545.1 | *Pseudomonas aeruginosa* | strain=2020CK-00220 | Complete Genome |
| GCF_028595865.2 | *Pseudomonas aeruginosa* | strain=P9 | Complete Genome |
| GCF_029961565.1 | *Pseudomonas aeruginosa* | strain=2020CK-00218 | Complete Genome |
| GCF_029961265.1 | *Pseudomonas aeruginosa* | strain=2021CK-01157 | Complete Genome |
| GCF_029961465.1 | *Pseudomonas aeruginosa* | strain=2021CK-01445 | Complete Genome |
| GCF_029961665.1 | *Pseudomonas aeruginosa* | strain=2021CK-01256 | Complete Genome |
| GCF_029961385.1 | *Pseudomonas aeruginosa* | strain=2021CK-01227 | Complete Genome |
| GCF_029961645.1 | *Pseudomonas aeruginosa* | strain=2022CK-00096 | Complete Genome |
| GCF_029958385.1 | *Pseudomonas aeruginosa* | strain=Li010 | Complete Genome |
| GCF_029962165.1 | *Pseudomonas aeruginosa* | strain=2022CK-00068 | Complete Genome |
| GCF_029962145.1 | *Pseudomonas aeruginosa* | strain=2020CK-00443 | Complete Genome |
| GCF_029961285.1 | *Pseudomonas aeruginosa* | strain=2021CK-01161 | Complete Genome |
| GCF_029961525.1 | *Pseudomonas aeruginosa* | strain=2020CK-00185 | Complete Genome |
| GCF_029961345.1 | *Pseudomonas aeruginosa* | strain=2021CK-01198 | Complete Genome |
| GCF_030034615.1 | *Pseudomonas aeruginosa* | strain=ZY94 | Complete Genome |
| GCF_030034655.1 | *Pseudomonas aeruginosa* | strain=ZY36 | Complete Genome |
| GCF_030122035.1 | *Pseudomonas aeruginosa* | strain=NY5532 | Complete Genome |
| GCF_030121975.1 | *Pseudomonas aeruginosa* | strain=NY5523 | Complete Genome |
| GCF_030121995.1 | *Pseudomonas aeruginosa* | strain=NY5520 | Complete Genome |
| GCF_030122015.1 | *Pseudomonas aeruginosa* | strain=NY5530 | Complete Genome |
| GCF_030034635.1 | *Pseudomonas aeruginosa* | strain=ZY156 | Complete Genome |
| GCF_030121935.1 | *Pseudomonas aeruginosa* | strain=NY5524 | Complete Genome |
| GCF_030121955.1 | *Pseudomonas aeruginosa* | strain=NY5525 | Complete Genome |
| GCF_030121875.1 | *Pseudomonas aeruginosa* | strain=NY5507 | Complete Genome |
| GCF_030122115.1 | *Pseudomonas aeruginosa* | strain=NY11254 | Complete Genome |
| GCF_030121895.1 | *Pseudomonas aeruginosa* | strain=NY5510 | Complete Genome |
| GCF_030121915.1 | *Pseudomonas aeruginosa* | strain=NY5511 | Complete Genome |
| GCF_030028275.1 | *Pseudomonas aeruginosa* | strain=SF416 | Complete Genome |
| GCF_030034675.1 | *Pseudomonas aeruginosa* | strain=ZY1710 | Complete Genome |
| GCF_030122055.1 | *Pseudomonas aeruginosa* | strain=NY5535 | Complete Genome |
| GCF_030122075.1 | *Pseudomonas aeruginosa* | strain=NY11173 | Complete Genome |
| GCF_030122135.1 | *Pseudomonas aeruginosa* | strain=NY13932 | Complete Genome |
| GCF_030122155.1 | *Pseudomonas aeruginosa* | strain=NY13936 | Complete Genome |
| GCF_030121855.1 | *Pseudomonas aeruginosa* | strain=NY5506 | Complete Genome |
| GCF_030122095.1 | *Pseudomonas aeruginosa* | strain=NY11210 | Complete Genome |
| GCF_022700635.2 | *Pseudomonas aeruginosa* | strain=PAD8 | Complete Genome |
| GCF_951691365.1 | *Pseudomonas aeruginosa* | strain=4782MK | Complete Genome |
| GCF_951805275.2 | *Pseudomonas aeruginosa* | strain=3796A | Complete Genome |
| GCF_030253475.1 | *Pseudomonas aeruginosa* | strain=22112 | Complete Genome |
| GCF_951802375.2 | *Pseudomonas aeruginosa* | strain=3541 | Complete Genome |
| GCF_030369855.1 | *Pseudomonas aeruginosa* | strain=WS27-3 | Complete Genome |
| GCF_030369835.1 | *Pseudomonas aeruginosa* | strain=Fe11-1 | Complete Genome |
| GCF_030369815.1 | *Pseudomonas aeruginosa* | strain=Fe8-1 | Complete Genome |
| GCF_030388285.1 | *Pseudomonas aeruginosa* | strain=LG2-2 | Complete Genome |
| GCF_030444495.1 | *Pseudomonas aeruginosa* | strain=CW41 | Complete Genome |
| GCF_030436095.1 | *Pseudomonas aeruginosa* | strain=PA272 | Complete Genome |
| GCF_030444635.1 | *Pseudomonas aeruginosa* | strain=TNP004 | Complete Genome |
| GCF_030413195.1 | *Pseudomonas aeruginosa* | strain=PA942 | Complete Genome |
| GCF_030444675.1 | *Pseudomonas aeruginosa* | strain=LG03 | Complete Genome |
| GCF_030489625.1 | *Pseudomonas aeruginosa* | strain=PA2500 | Complete Genome |
| GCF_030489645.1 | *Pseudomonas aeruginosa* | strain=PA2209 | Complete Genome |
| GCF_030490735.1 | *Pseudomonas aeruginosa* | strain=PA2818 | Complete Genome |
| GCF_030505595.1 | *Pseudomonas aeruginosa* | strain=PA1045 | Complete Genome |
| GCF_030563765.1 | *Pseudomonas aeruginosa* | strain=AU10241 | Complete Genome |
| GCF_030564045.1 | *Pseudomonas aeruginosa* | strain=AU10241 | Complete Genome |
| GCF_030563585.1 | *Pseudomonas aeruginosa* | strain=AU10241 | Complete Genome |
| GCF_028366415.1 | *Pseudomonas aeruginosa* | strain=2023CK-00048 | Complete Genome |
| GCF_030758435.1 | *Pseudomonas aeruginosa* | strain=NY11352 | Complete Genome |
| GCF_030908445.1 | *Pseudomonas aeruginosa* | strain=002 | Complete Genome |
| GCF_030908425.1 | *Pseudomonas aeruginosa* | strain=pae001 | Complete Genome |
| GCF_021497365.2 | *Pseudomonas aeruginosa* | strain=SE5418 | Complete Genome |
| GCF_030866905.1 | *Pseudomonas aeruginosa* | strain=ZYPA192 | Complete Genome |
| GCF_030866885.1 | *Pseudomonas aeruginosa* | strain=SRMPA3860 | Complete Genome |
| GCF_031212645.1 | *Pseudomonas aeruginosa* | strain=NY11084 | Complete Genome |
| GCF_031221745.1 | *Pseudomonas aeruginosa* | strain=NY7570 | Complete Genome |
| GCF_031223175.1 | *Pseudomonas aeruginosa* | strain=NY4383 | Complete Genome |
| GCF_031224875.1 | *Pseudomonas aeruginosa* | strain=NY11384 | Complete Genome |
| GCF_031218375.1 | *Pseudomonas aeruginosa* | strain=NY11104 | Complete Genome |
| GCF_031211485.1 | *Pseudomonas aeruginosa* | strain=SE5356 | Complete Genome |
| GCF_031220265.1 | *Pseudomonas aeruginosa* | strain=NY7723 | Complete Genome |
| GCF_031224255.1 | *Pseudomonas aeruginosa* | strain=NY7583 | Complete Genome |
| GCF_031326325.1 | *Pseudomonas aeruginosa* | strain=ZYPA187 | Complete Genome |
| GCF_031222225.1 | *Pseudomonas aeruginosa* | strain=NY7483 | Complete Genome |
| GCF_031219395.1 | *Pseudomonas aeruginosa* | strain=NY5051 | Complete Genome |
| GCF_031326305.1 | *Pseudomonas aeruginosa* | strain=ZYPA1927 | Complete Genome |
| GCF_021497345.2 | *Pseudomonas aeruginosa* | strain=SE5381 | Complete Genome |
| GCF_031851725.1 | *Pseudomonas aeruginosa* | strain=TJM4 | Complete Genome |
| GCF_032231105.1 | *Pseudomonas aeruginosa* | strain=Paer3125 | Complete Genome |
| GCF_032231085.1 | *Pseudomonas aeruginosa* | strain=Paer2090 | Complete Genome |
| GCF_032231025.1 | *Pseudomonas aeruginosa* | strain=Paer3771 | Complete Genome |
| GCF_032231045.1 | *Pseudomonas aeruginosa* | strain=Paer3554 | Complete Genome |
| GCF_032463705.1 | *Pseudomonas aeruginosa* | strain=R18-20 | Complete Genome |
| GCF_032252955.1 | *Pseudomonas aeruginosa* | strain=PARM_L1 | Complete Genome |
| GCF_032252975.1 | *Pseudomonas aeruginosa* | strain=PARM_Z1 | Complete Genome |
| GCF_032252935.1 | *Pseudomonas aeruginosa* | strain=CP-1 | Complete Genome |
| GCF_032811685.1 | *Pseudomonas aeruginosa* | strain=GOM9 | Complete Genome |
| GCF_032761935.1 | *Pseudomonas aeruginosa* | strain=XM8 | Complete Genome |
| GCF_032598925.1 | *Pseudomonas aeruginosa* | strain=NY3649 | Complete Genome |
| GCF_032681185.1 | *Pseudomonas aeruginosa* | strain=UO299 | Complete Genome |
| GCF_032696765.1 | *Pseudomonas aeruginosa* | strain=2718 | Complete Genome |
| GCF_032941915.1 | *Pseudomonas aeruginosa* | strain=A6-17 | Complete Genome |
| GCF_032699645.1 | *Pseudomonas aeruginosa* | strain=8247 | Complete Genome |
| GCF_032847385.1 | *Pseudomonas aeruginosa* | strain=PA12 | Complete Genome |
| GCF_033224565.1 | *Pseudomonas aeruginosa* | strain=HPA0384 | Complete Genome |
| GCF_033228545.1 | *Pseudomonas aeruginosa* | strain=HPA0310 | Complete Genome |
| GCF_033223225.1 | *Pseudomonas aeruginosa* | strain=HPA0663 | Complete Genome |
| GCF_033221155.1 | *Pseudomonas aeruginosa* | strain=HPA1346 | Complete Genome |
| GCF_033335935.1 | *Pseudomonas aeruginosa* | strain=CDC1270 | Complete Genome |
| GCF_033222185.1 | *Pseudomonas aeruginosa* | strain=HPA0875 | Complete Genome |
| GCF_033217895.1 | *Pseudomonas aeruginosa* | strain=HPA2120 | Complete Genome |
| GCF_033225445.1 | *Pseudomonas aeruginosa* | strain=HPA0124 | Complete Genome |
| GCF_033226745.1 | *Pseudomonas aeruginosa* | strain=HPA0044 | Complete Genome |
| GCF_033394235.1 | *Pseudomonas aeruginosa* | na | Complete Genome |
| GCF_033395015.1 | *Pseudomonas aeruginosa* | na | Complete Genome |
| GCF_033232085.1 | *Pseudomonas aeruginosa* | strain=HPA0118 | Complete Genome |
| GCF_033216995.1 | *Pseudomonas aeruginosa* | strain=HPA2660 | Complete Genome |
| GCF_033219955.1 | *Pseudomonas aeruginosa* | strain=HPA1406 | Complete Genome |
| GCF_033230305.1 | *Pseudomonas aeruginosa* | strain=HPA0170 | Complete Genome |
| GCF_033227745.1 | *Pseudomonas aeruginosa* | strain=HPA0698 | Complete Genome |
| GCF_033219075.1 | *Pseudomonas aeruginosa* | strain=HPA2017 | Complete Genome |
| GCF_033229485.1 | *Pseudomonas aeruginosa* | strain=HPA0271 | Complete Genome |
| GCF_033393195.1 | *Pseudomonas aeruginosa* | na | Complete Genome |
| GCF_033392255.1 | *Pseudomonas aeruginosa* | strain=PAO1F | Complete Genome |
| GCF_033615795.1 | *Pseudomonas aeruginosa* | strain=JBPA | Complete Genome |
| GCF_033807835.1 | *Pseudomonas aeruginosa* | strain=strain KUD2 | Complete Genome |
| GCF_034190815.1 | *Pseudomonas aeruginosa* | strain=136790 | Complete Genome |
| GCF_032292255.1 | *Pseudomonas aeruginosa* | strain=2023CK-01249 | Complete Genome |
| GCF_034438375.1 | *Pseudomonas aeruginosa* | strain=19-3158 | Complete Genome |
| GCF_034480705.1 | *Pseudomonas aeruginosa* | strain=FI-14/157 | Complete Genome |
| GCF_034480885.1 | *Pseudomonas aeruginosa* | strain=FI-17645 | Complete Genome |
| GCF_033375015.1 | *Pseudomonas aeruginosa* | strain=CUVET20-956 | Complete Genome |
| GCF_033375815.1 | *Pseudomonas aeruginosa* | strain=CUVET18-860 | Complete Genome |
| GCF_033374175.1 | *Pseudomonas aeruginosa* | strain=CUVET21-397 | Complete Genome |
| GCF_033971305.1 | *Pseudomonas aeruginosa* | strain=DB-1 | Complete Genome |
| GCF_035197065.1 | *Pseudomonas aeruginosa* | strain=CPA0053 | Complete Genome |
| GCF_035197965.1 | *Pseudomonas aeruginosa* | strain=CPA0087 | Complete Genome |
| GCF_035205985.1 | *Pseudomonas aeruginosa* | strain=BJ86 | Complete Genome |
| GCF_035621175.1 | *Pseudomonas aeruginosa* | strain=2023CK-01336 | Complete Genome |
| GCF_035621255.1 | *Pseudomonas aeruginosa* | strain=ZM21 | Complete Genome |
| GCF_035621395.1 | *Pseudomonas aeruginosa* | strain=PA14-GFP | Complete Genome |
| GCF_035666215.1 | *Pseudomonas aeruginosa* | strain=L3 | Complete Genome |
| GCF_035984435.1 | *Pseudomonas aeruginosa* | strain=Tongji | Complete Genome |
| GCF_036233155.1 | *Pseudomonas aeruginosa* | strain=F034 | Complete Genome |
| GCF_036232085.1 | *Pseudomonas aeruginosa* | strain=F078 | Complete Genome |
| GCF_036233435.1 | *Pseudomonas aeruginosa* | strain=F020 | Complete Genome |
| GCF_036237095.1 | *Pseudomonas aeruginosa* | strain=F002 | Complete Genome |
| GCF_036232645.1 | *Pseudomonas aeruginosa* | strain=F053 | Complete Genome |
| GCF_036232265.1 | *Pseudomonas aeruginosa* | strain=F069 | Complete Genome |
| GCF_036233255.1 | *Pseudomonas aeruginosa* | strain=F029 | Complete Genome |
| GCF_036233555.1 | *Pseudomonas aeruginosa* | strain=F014 | Complete Genome |
| GCF_036232565.1 | *Pseudomonas aeruginosa* | strain=F057 | Complete Genome |
| GCF_036232905.1 | *Pseudomonas aeruginosa* | strain=F040 | Complete Genome |
| GCF_036233315.1 | *Pseudomonas aeruginosa* | strain=F026 | Complete Genome |
| GCF_036233635.1 | *Pseudomonas aeruginosa* | strain=F010 | Complete Genome |
| GCF_036232125.1 | *Pseudomonas aeruginosa* | strain=F076 | Complete Genome |
| GCF_036232185.1 | *Pseudomonas aeruginosa* | strain=F073 | Complete Genome |
| GCF_036232145.1 | *Pseudomonas aeruginosa* | strain=F075 | Complete Genome |
| GCF_036232745.1 | *Pseudomonas aeruginosa* | strain=F048 | Complete Genome |
| GCF_036233175.1 | *Pseudomonas aeruginosa* | strain=F033 | Complete Genome |
| GCF_036233595.1 | *Pseudomonas aeruginosa* | strain=F012 | Complete Genome |
| GCF_036237115.1 | *Pseudomonas aeruginosa* | strain=F001 | Complete Genome |
| GCF_036233195.1 | *Pseudomonas aeruginosa* | strain=F032 | Complete Genome |
| GCF_036232585.1 | *Pseudomonas aeruginosa* | strain=F056 | Complete Genome |
| GCF_036233215.1 | *Pseudomonas aeruginosa* | strain=F031 | Complete Genome |
| GCF_036233535.1 | *Pseudomonas aeruginosa* | strain=F015 | Complete Genome |
| GCF_036233755.1 | *Pseudomonas aeruginosa* | strain=F004 | Complete Genome |
| GCF_036232205.1 | *Pseudomonas aeruginosa* | strain=F072 | Complete Genome |
| GCF_036232405.1 | *Pseudomonas aeruginosa* | strain=F062 | Complete Genome |
| GCF_036232825.1 | *Pseudomonas aeruginosa* | strain=F044 | Complete Genome |
| GCF_036233115.1 | *Pseudomonas aeruginosa* | strain=F036 | Complete Genome |
| GCF_036233135.1 | *Pseudomonas aeruginosa* | strain=F035 | Complete Genome |
| GCF_036233495.1 | *Pseudomonas aeruginosa* | strain=F017 | Complete Genome |
| GCF_036237075.1 | *Pseudomonas aeruginosa* | strain=20 | Complete Genome |
| GCF_036233715.1 | *Pseudomonas aeruginosa* | strain=F006 | Complete Genome |
| GCF_036232455.1 | *Pseudomonas aeruginosa* | strain=F060 | Complete Genome |
| GCF_036233355.1 | *Pseudomonas aeruginosa* | strain=F024 | Complete Genome |
| GCF_036233655.1 | *Pseudomonas aeruginosa* | strain=F009 | Complete Genome |
| GCF_036232865.1 | *Pseudomonas aeruginosa* | strain=F042 | Complete Genome |
| GCF_036232945.1 | *Pseudomonas aeruginosa* | strain=F038 | Complete Genome |
| GCF_036233615.1 | *Pseudomonas aeruginosa* | strain=F011 | Complete Genome |
| GCF_036232345.1 | *Pseudomonas aeruginosa* | strain=F065 | Complete Genome |
| GCF_036232285.1 | *Pseudomonas aeruginosa* | strain=F068 | Complete Genome |
| GCF_036418055.1 | *Pseudomonas aeruginosa* | strain=PA12_L1_25.22_ST207 | Complete Genome |
| GCF_036418275.1 | *Pseudomonas aeruginosa* | strain=PA7_SE3_14.22_ST654_NDM1 | Complete Genome |
| GCF_036417915.1 | *Pseudomonas aeruginosa* | strain=PA26_WM3_03.23_ST27 | Complete Genome |
| GCF_036232105.1 | *Pseudomonas aeruginosa* | strain=F077 | Complete Genome |
| GCF_036236975.1 | *Pseudomonas aeruginosa* | strain=214 | Complete Genome |
| GCF_036232495.1 | *Pseudomonas aeruginosa* | strain=F059 | Complete Genome |
| GCF_036232305.1 | *Pseudomonas aeruginosa* | strain=F067 | Complete Genome |
| GCF_036232525.1 | *Pseudomonas aeruginosa* | strain=F058 | Complete Genome |
| GCF_036233295.1 | *Pseudomonas aeruginosa* | strain=F027 | Complete Genome |
| GCF_036233475.1 | *Pseudomonas aeruginosa* | strain=F018 | Complete Genome |
| GCF_036236015.1 | *Pseudomonas aeruginosa* | strain=382 | Complete Genome |
| GCF_036232245.1 | *Pseudomonas aeruginosa* | strain=F070 | Complete Genome |
| GCF_036233395.1 | *Pseudomonas aeruginosa* | strain=F022 | Complete Genome |
| GCF_036232325.1 | *Pseudomonas aeruginosa* | strain=F066 | Complete Genome |
| GCF_036232385.1 | *Pseudomonas aeruginosa* | strain=F063 | Complete Genome |
| GCF_036232725.1 | *Pseudomonas aeruginosa* | strain=F049 | Complete Genome |
| GCF_036237015.1 | *Pseudomonas aeruginosa* | strain=188 | Complete Genome |
| GCF_036232425.1 | *Pseudomonas aeruginosa* | strain=F061 | Complete Genome |
| GCF_036233095.1 | *Pseudomonas aeruginosa* | strain=F037 | Complete Genome |
| GCF_036237055.1 | *Pseudomonas aeruginosa* | strain=37 | Complete Genome |
| GCF_036232845.1 | *Pseudomonas aeruginosa* | strain=F043 | Complete Genome |
| GCF_036232885.1 | *Pseudomonas aeruginosa* | strain=F041 | Complete Genome |
| GCF_036236995.1 | *Pseudomonas aeruginosa* | strain=199 | Complete Genome |
| GCF_036232065.1 | *Pseudomonas aeruginosa* | strain=LRJ32 | Complete Genome |
| GCF_036232165.1 | *Pseudomonas aeruginosa* | strain=F074 | Complete Genome |
| GCF_036232605.1 | *Pseudomonas aeruginosa* | strain=F055 | Complete Genome |
| GCF_036232705.1 | *Pseudomonas aeruginosa* | strain=F050 | Complete Genome |
| GCF_036232925.1 | *Pseudomonas aeruginosa* | strain=F039 | Complete Genome |
| GCF_036418125.1 | *Pseudomonas aeruginosa* | strain=PA13_L17_27.22_ST207 | Complete Genome |
| GCF_036417705.1 | *Pseudomonas aeruginosa* | strain=PA27_WM3_03.23_ST27 | Complete Genome |
| GCF_036417805.1 | *Pseudomonas aeruginosa* | strain=PA11_WM3_21.22_ST27 | Complete Genome |
| GCF_036233375.1 | *Pseudomonas aeruginosa* | strain=F023 | Complete Genome |
| GCF_036233695.1 | *Pseudomonas aeruginosa* | strain=F007 | Complete Genome |
| GCF_036232365.1 | *Pseudomonas aeruginosa* | strain=F064 | Complete Genome |
| GCF_036232685.1 | *Pseudomonas aeruginosa* | strain=F051 | Complete Genome |
| GCF_036233415.1 | *Pseudomonas aeruginosa* | strain=F021 | Complete Genome |
| GCF_036232625.1 | *Pseudomonas aeruginosa* | strain=F054 | Complete Genome |
| GCF_036232665.1 | *Pseudomonas aeruginosa* | strain=F052 | Complete Genome |
| GCF_036232225.1 | *Pseudomonas aeruginosa* | strain=F071 | Complete Genome |
| GCF_036233575.1 | *Pseudomonas aeruginosa* | strain=F013 | Complete Genome |
| GCF_036234315.1 | *Pseudomonas aeruginosa* | strain=F003 | Complete Genome |
| GCF_036237035.1 | *Pseudomonas aeruginosa* | strain=135 | Complete Genome |
| GCF_036233275.1 | *Pseudomonas aeruginosa* | strain=F028 | Complete Genome |
| GCF_036233455.1 | *Pseudomonas aeruginosa* | strain=F019 | Complete Genome |
| GCF_036232805.1 | *Pseudomonas aeruginosa* | strain=F045 | Complete Genome |
| GCF_036233235.1 | *Pseudomonas aeruginosa* | strain=F030 | Complete Genome |
| GCF_036233335.1 | *Pseudomonas aeruginosa* | strain=F025 | Complete Genome |
| GCF_036232765.1 | *Pseudomonas aeruginosa* | strain=F047 | Complete Genome |
| GCF_036233515.1 | *Pseudomonas aeruginosa* | strain=F016 | Complete Genome |
| GCF_036233735.1 | *Pseudomonas aeruginosa* | strain=F005 | Complete Genome |
| GCF_036418145.1 | *Pseudomonas aeruginosa* | strain=PA18_L3_44.22_ST308_NDM1 | Complete Genome |
| GCF_036417995.1 | *Pseudomonas aeruginosa* | strain=PA19_NI3_44.22_ST207 | Complete Genome |
| GCF_036232785.1 | *Pseudomonas aeruginosa* | strain=F046 | Complete Genome |
| GCF_036233675.1 | *Pseudomonas aeruginosa* | strain=F008 | Complete Genome |
| GCF_036326305.1 | *Pseudomonas aeruginosa* | strain=2023CK-01621 | Complete Genome |
| GCF_022424055.2 | *Pseudomonas aeruginosa* | strain=14182 | Complete Genome |
| GCF_036542105.1 | *Pseudomonas aeruginosa* | strain=CH1 | Complete Genome |
| GCF_036418185.1 | *Pseudomonas aeruginosa* | strain=PA15_L5_37.22_ST773_VIM2 | Complete Genome |
| GCF_036418295.1 | *Pseudomonas aeruginosa* | strain=PA4_YH3_36.21_ST773_NDM1 | Complete Genome |
| GCF_037285345.1 | *Pseudomonas aeruginosa* | strain=GN05219 | Complete Genome |
| GCF_037287545.1 | *Pseudomonas aeruginosa* | strain=GN06858 | Complete Genome |
| GCF_037287535.1 | *Pseudomonas aeruginosa* | strain=GN06251 | Complete Genome |
| GCF_037287585.1 | *Pseudomonas aeruginosa* | strain=GN04844 | Complete Genome |
| GCF_037287465.1 | *Pseudomonas aeruginosa* | strain=GN06816 | Complete Genome |
| GCF_037287505.1 | *Pseudomonas aeruginosa* | strain=GN05356 | Complete Genome |
| GCF_037482125.1 | *Pseudomonas aeruginosa* | strain=S1 | Complete Genome |
| GCF_030183855.2 | *Pseudomonas aeruginosa* | strain=CPO459 | Complete Genome |
| GCF_035557195.1 | *Pseudomonas aeruginosa* | strain=2024CK-00032 | Complete Genome |
| GCF_030183355.2 | *Pseudomonas aeruginosa* | strain=CPO506 | Complete Genome |
| GCF_030185815.2 | *Pseudomonas aeruginosa* | strain=CPO180 | Complete Genome |
| GCF_037914935.1 | *Pseudomonas aeruginosa* | na | Complete Genome |
| GCF_037287405.1 | *Pseudomonas aeruginosa* | strain=GN04922 | Complete Genome |
| GCF_030185915.2 | *Pseudomonas aeruginosa* | strain=CPO174 | Complete Genome |
| GCF_030186885.2 | *Pseudomonas aeruginosa* | strain=CPO100 | Complete Genome |
| GCF_037267025.1 | *Pseudomonas aeruginosa* | strain=GN06288 | Complete Genome |
| GCF_037287425.1 | *Pseudomonas aeruginosa* | strain=GN04821 | Complete Genome |
| GCF_036835625.1 | *Pseudomonas aeruginosa* | strain=IPA34 | Complete Genome |
| GCF_036776515.1 | *Pseudomonas aeruginosa* | strain=1709-25403 | Complete Genome |
| GCF_037890725.1 | *Pseudomonas aeruginosa* | strain=2023CK-01620 | Complete Genome |
| GCF_038396885.1 | *Pseudomonas aeruginosa* | strain=HN232 | Complete Genome |
| GCF_038396595.1 | *Pseudomonas aeruginosa* | strain=LYSZa2 | Complete Genome |
| GCF_038397815.1 | *Pseudomonas aeruginosa* | strain=HN148 | Complete Genome |
| GCF_038396855.1 | *Pseudomonas aeruginosa* | strain=PA98zyfcau | Complete Genome |
| GCF_032843885.3 | *Pseudomonas aeruginosa* | strain=PSA9 | Complete Genome |
| GCF_038435155.1 | *Pseudomonas aeruginosa* | strain=2014S01-136 | Complete Genome |
| GCF_038431935.1 | *Pseudomonas aeruginosa* | strain=2014S09-209 | Complete Genome |
| GCF_038431925.1 | *Pseudomonas aeruginosa* | strain=2016S01-136 | Complete Genome |
| GCF_038431895.1 | *Pseudomonas aeruginosa* | strain=2016S09-057 | Complete Genome |
| GCF_038431905.1 | *Pseudomonas aeruginosa* | strain=2016N06-105 | Complete Genome |
| GCF_038433795.1 | *Pseudomonas aeruginosa* | strain=2014S05-249 | Complete Genome |
| GCF_038420425.1 | *Pseudomonas aeruginosa* | strain=18083286 | Complete Genome |
| GCF_038431915.1 | *Pseudomonas aeruginosa* | strain=2016C02-074 | Complete Genome |
| GCF_038431885.1 | *Pseudomonas aeruginosa* | strain=2016C06-003 | Complete Genome |
| GCF_038432515.1 | *Pseudomonas aeruginosa* | strain=2014S06-172 | Complete Genome |
| GCF_038431945.1 | *Pseudomonas aeruginosa* | strain=2014S07-062 | Complete Genome |
| GCF_019084125.3 | *Pseudomonas aeruginosa* | strain=NCTR 501 | Complete Genome |
| GCF_039622765.1 | *Pseudomonas aeruginosa* | strain=PA_HN004 | Complete Genome |
| GCF_039622885.1 | *Pseudomonas aeruginosa* | strain=PA_HN006 | Complete Genome |
| GCF_039622815.1 | *Pseudomonas aeruginosa* | strain=PA_HN005 | Complete Genome |
| GCF_039555165.1 | *Pseudomonas aeruginosa* | strain=CUVET23-830 | Complete Genome |
| GCF_039512425.1 | *Pseudomonas aeruginosa* | strain=PAE3 | Complete Genome |
| GCF_023572735.3 | *Pseudomonas aeruginosa* | strain=CCBH26428 | Complete Genome |
| GCF_039702325.1 | *Pseudomonas aeruginosa* | strain=TY922 | Complete Genome |
| GCF_036688165.2 | *Pseudomonas aeruginosa* | strain=3860P | Complete Genome |
| GCF_039622755.1 | *Pseudomonas aeruginosa* | na | Complete Genome |
| GCF_030866925.2 | *Pseudomonas aeruginosa* | strain=ZYPA162 | Complete Genome |
| GCF_039830125.1 | *Pseudomonas aeruginosa* | strain=P2550 | Complete Genome |
| GCF_039904215.1 | *Pseudomonas aeruginosa* | strain=MPAO1 | Complete Genome |
| GCF_039908075.1 | *Pseudomonas aeruginosa* | strain=FIDG-26323 | Complete Genome |
| GCF_039908025.1 | *Pseudomonas aeruginosa* | strain=FI-26059 | Complete Genome |
| GCF_038069335.1 | *Pseudomonas aeruginosa* | strain=2024CK-00357 | Complete Genome |
| GCF_039919425.1 | *Pseudomonas aeruginosa* | strain=PAO1DF | Complete Genome |
| GCF_038069315.1 | *Pseudomonas aeruginosa* | strain=2024CK-00356 | Complete Genome |
| GCF_038418245.1 | *Pseudomonas aeruginosa* | strain=2024CK-00455 | Complete Genome |
| GCF_039921825.1 | *Pseudomonas aeruginosa* | na | Complete Genome |
| GCF_039924275.1 | *Pseudomonas aeruginosa* | na | Complete Genome |
| GCF_040008295.1 | *Pseudomonas aeruginosa* | strain=mPAO1 | Complete Genome |
| GCF_040268335.1 | *Pseudomonas aeruginosa* | strain=SRPA1308 | Complete Genome |
| GCF_040084805.1 | *Pseudomonas aeruginosa* | strain=NPA09B | Complete Genome |
| GCF_040562075.1 | *Pseudomonas aeruginosa* | strain=PA3117 | Complete Genome |
| GCF_040429605.1 | *Pseudomonas aeruginosa* | strain=P1110 | Complete Genome |
| GCF_040429595.1 | *Pseudomonas aeruginosa* | strain=Y010 | Complete Genome |
| GCF_040581215.1 | *Pseudomonas aeruginosa* | strain=P113 | Complete Genome |
| GCF_040580825.1 | *Pseudomonas aeruginosa* | strain=T117 | Complete Genome |
| GCF_040571335.1 | *Pseudomonas aeruginosa* | strain=100690 | Complete Genome |
| GCF_040560445.1 | *Pseudomonas aeruginosa* | strain=PA64 | Complete Genome |
| GCF_040581105.1 | *Pseudomonas aeruginosa* | strain=P118 | Complete Genome |
| GCF_040735975.1 | *Pseudomonas aeruginosa* | strain=CI00795 | Complete Genome |
| GCF_040931625.1 | *Pseudomonas aeruginosa* | strain=1001d0_1 | Complete Genome |
| GCF_040931635.1 | *Pseudomonas aeruginosa* | strain=0303d1.1 | Complete Genome |
| GCF_000014625.1 | *Pseudomonas aeruginosa* UCBPP-PA14 | strain=UCBPP-PA14 | Complete Genome |
| GCF_000006765.1 | *Pseudomonas aeruginosa* PAO1 | strain=PAO1 | Complete Genome |
| GCF_013001005.1 | *Pseudomonas aeruginosa* PAO1 | strain=PAO1 | Complete Genome |
| GCF_030444655.1 | *Pseudomonas aeruginosa* PAO1 | strain=PAO1 | Complete Genome |
| GCF_030444595.1 | *Pseudomonas aeruginosa* PAO1 | strain=PAO1 | Complete Genome |
| GCF_000017205.1 | *Pseudomonas aeruginosa* PA7 | strain=PA7 | Complete Genome |
| GCF_000168335.1 | *Pseudomonas aeruginosa* PACS2 | strain=PACS2 | Complete Genome |
| GCF_000026645.1 | *Pseudomonas aeruginosa* LESB58 | strain=LESB58 | Complete Genome |
| GCF_030264075.1 | *Pseudomonas aeruginosa* PA14 | strain=MA3 | Complete Genome |
| GCF_900185255.1 | *Pseudomonas aeruginosa* C-NN2 | na | Complete Genome |
| GCF_000226155.1 | *Pseudomonas aeruginosa* M18 | strain=M18 | Complete Genome |
| GCF_902172305.2 | *Pseudomonas aeruginosa* PAK | strain=PAK | Complete Genome |
| GCF_000223945.1 | *Pseudomonas aeruginosa* 19BR | strain=19BR | Complete Genome |
| GCF_000223965.1 | *Pseudomonas aeruginosa* 213BR | strain=213BR | Complete Genome |
| GCF_033108345.1 | *Pseudomonas aeruginosa* PA103 | strain=PA103 | Complete Genome |
| GCF_000284555.1 | *Pseudomonas aeruginosa* NCGM2.S1 | strain=NCGM2.S1 | Complete Genome |
| GCF_000271365.1 | *Pseudomonas aeruginosa* DK2 | strain=DK2 | Complete Genome |
| GCF_001045685.1 | *Pseudomonas aeruginosa* DSM 50071 = NBRC 12689 | strain=DSM 50071 | Complete Genome |
| GCF_025079255.1 | *Pseudomonas aeruginosa* TBCF10839 | strain=TBCF10839 | Complete Genome |
| GCF_022810825.1 | *Pseudomonas aeruginosa* CI27 | strain=CI27 | Complete Genome |
| GCF_000271985.2 | *Pseudomonas aeruginosa* SJTD-1 | strain=SJTD-1 | Complete Genome |
| GCF_000496605.2 | *Pseudomonas aeruginosa* PA1 | strain=PA1 | Complete Genome |
| GCF_000496645.1 | *Pseudomonas aeruginosa* PA1R | strain=PA1R | Complete Genome |
| GCF_000359505.1 | *Pseudomonas aeruginosa* B136-33 | strain=B136-33 | Complete Genome |
| GCF_000414035.1 | *Pseudomonas aeruginosa* RP73 | strain=RP73 | Complete Genome |
| GCF_900069025.1 | *Pseudomonas aeruginosa* DK1 | strain=DK1 substr. NH57388A | Complete Genome |
| GCF_000508765.1 | *Pseudomonas aeruginosa* LES431 | strain=LES431 | Complete Genome |
| GCF_000496455.2 | *Pseudomonas aeruginosa* DHS01 | strain=DH01 | Complete Genome |
| GCF_000504045.1 | *Pseudomonas aeruginosa* MTB-1 | strain=MTB-1 | Complete Genome |
| GCF_000510305.1 | *Pseudomonas aeruginosa* SCV20265 | strain=SCV20265 | Complete Genome |
| GCF_020978345.1 | *Pseudomonas aeruginosa* SG17M | strain=SG17M | Complete Genome |
| GCF_000524595.1 | *Pseudomonas aeruginosa* YL84 | strain=YL84 | Complete Genome |
