# Supplementary material for: Rational design of an epitope-centric vaccine against Pseudomonas aeruginosa using pangenomic insights and immunoinformatics approach
Source: Front Immunol. 2025 Sep 1;16:1617251. doi: 10.3389/fimmu.2025.1617251 (PMC12434008; doi:10.3389/fimmu.2025.1617251)
Supplement: Supplementary file 2 [file Table2.docx]

**Rational Design of an Epitope-Centric Vaccine Against *Pseudomonas aeruginosa* using Pangenomic Insights and Immunoinformatics Approach**

**Supplementary Table 2:** Overview of B-Cell Epitope Prediction

| No. | Start | End | Peptide | Length | Antigenicity (Threshold 0.4) | Allergenicity | Toxicity |
| --- | --- | --- | --- | --- | --- | --- | --- |
| 1 | 8 | 13 | LEQARS | 6 | -0.4836 ( Probable NON-ANTIGEN ). | NA | Non-toxic |
| 2 | 16 | 16 | S | 1 | NA | NA | NA |
| 3 | 18 | 37 | **LQSQPDATKVAALETKDAGD** | **20** | **0.7914 ( Probable ANTIGEN )** | Non-allergen | Non-toxic |
| 4 | 45 | 116 | AYQDGEDQRDVDQLAYLTNQRIELAK  QTIVLRNAEAQLQNASAQRAQARLDART  AQLDKLRSQLNAKQTSRG | 72 | Splited | NA | NA |
| 5 | 127 | 136 | **DLDKSDLKPG** | **10** | **1.1824 ( Probable ANTIGEN )** | Non-allergen | Non-toxic |
| 6 | 163 | 177 | DSTGSANYNQRLSER | 15 | 1.6064 ( Probable ANTIGEN ) | Allergen | Non-toxic |
| 7 | 191 | 193 | ISP | 3 | NA | NA | NA |
| 8 | 201 | 217 | **YGKEYPVASNGTSSGRA** | **17** | **1.3767 ( Probable ANTIGEN )** | Non-allergen | Non-toxic |
| 9 | 230 | 237 | AKPVAPRS | 8 | 0.0519 ( Probable NON-ANTIGEN ) | Non-allergen | Non-toxic |
| B Cell 72 MER | | | | | | | |
| No. | Start | End | Peptide | Length |  |  |  |
| 1 | 5 | 13 | **GEDQRDVDQ** | **9** | **1.4255 ( Probable ANTIGEN )** | Non-allergen | Non-toxic |
| 2 | 15 | 16 | AY | 2 | NA | NA | NA |
| 3 | 19 | 20 | NQ | 2 | NA | NA | NA |
| 4 | 22 | 26 | IELAK | 5 | NA | NA | NA |
| 5 | 42 | 51 | **SAQRAQARLD** | **10** | **1.2283 ( Probable ANTIGEN )** | Non-allergen | Non-toxic |
| 6 | 55 | 55 | A | 1 | NA | NA | NA |
| 7 | 57 | 57 | L | 1 | NA | NA | NA |
| 8 | 62 | 69 | **SQLNAKQT** | **8** | **1.4671 ( Probable ANTIGEN )** | Non-allergen | Non-toxic |
