# Supplementary material for: Rational design of an epitope-centric vaccine against Pseudomonas aeruginosa using pangenomic insights and immunoinformatics approach
Source: Front Immunol. 2025 Sep 1;16:1617251. doi: 10.3389/fimmu.2025.1617251 (PMC12434008; doi:10.3389/fimmu.2025.1617251)
Supplement: Supplementary file 3 [file Table3.docx]

**Rational Design of an Epitope-Centric Vaccine Against *Pseudomonas aeruginosa* using Pangenomic Insights and Immunoinformatics Approach**

**Supplementary Table 3:** Overview of MHC-I Epitope Prediction

| allele | length | Peptide | score | rank | Antigenicity Score | Probable antigen | Allergenicity | Toxicity |
| --- | --- | --- | --- | --- | --- | --- | --- | --- |
| HLA-A*01:01 | 10 | YTDSTGSANY | 0.995492 | 0.01 | 1.3013 | Antigen | Non-Allergen | Non-Toxin |
| HLA-B*07:02 | 10 | AKPVAPRSSV | 0.91629 | 0.04 | 0.1042 | Non-Antigen | Non-Allergen | Non-Toxin |
| HLA-A*68:01 | 10 | ELAKQTIVLR | 0.867629 | 0.12 | 0.2555 | Non-Antigen | Allergen | Non-Toxin |
| HLA-B*07:02 | 10 | QPDATKVAAL | 0.827177 | 0.07 | 0.5837 | Antigen | Allergen | Non-Toxin |
| HLA-B*57:01 | 10 | QTSRGTMVTF | 0.789164 | 0.22 | 0.5176 | Antigen | Non-Allergen | Non-Toxin |
| HLA-A*03:01 | 10 | ALQSQPDATK | 0.736163 | 0.14 | 1.0414 | Antigen | Allergen | Non-Toxin |
| HLA-B*40:01 | 10 | GEDQRDVDQL | 0.686557 | 0.16 | 1.0027 | Antigen | Non-Allergen | Non-Toxin |
| HLA-A*33:01 | 10 | ELAKQTIVLR | 0.672752 | 0.08 | 0.2555 | Non-Antigen | Allergen | Non-Toxin |
| HLA-A*68:01 | 10 | STGSANYNQR | 0.668637 | 0.35 | 1.4014 | Antigen | Allergen | Non-Toxin |
| HLA-A*11:01 | 10 | ATKVAALETK | 0.667652 | 0.16 | 0.8269 | Antigen | Allergen | Non-Toxin |
| HLA-B*40:01 | 10 | IELAKQTIVL | 0.663865 | 0.18 | -0.191 | Non-Antigen | Non-Allergen | Non-Toxin |
| HLA-A*31:01 | 10 | KSDLKPGAMR | 0.645193 | 0.19 | 0.9618 | Antigen | Non-Allergen | Non-Toxin |
