# Supplementary material for: Rational design of an epitope-centric vaccine against Pseudomonas aeruginosa using pangenomic insights and immunoinformatics approach
Source: Front Immunol. 2025 Sep 1;16:1617251. doi: 10.3389/fimmu.2025.1617251 (PMC12434008; doi:10.3389/fimmu.2025.1617251)
Supplement: Supplementary file 4 [file Table4.docx]

**Rational Design of an Epitope-Centric Vaccine Against *Pseudomonas aeruginosa* using Pangenomic Insights and Immunoinformatics Approach**

**Supplementary Table 4:** Overview of MHC-II Epitope Prediction

| allele | length | peptide | score | rank | Antigenicity Score | Probable antigen | Allergenicity | INFGamma | |
| --- | --- | --- | --- | --- | --- | --- | --- | --- | --- |
| HLA-DRB1*04:05 | 15 | RSNFSALQSQPDATK | 0.9839 | 0.01 | 0.7127 | Antigen | Non-Allergen | -0.063505068 | Negative |
| HLA-DRB1*04:05 | 15 | ARSNFSALQSQPDAT | 0.9798 | 0.01 | 0.743 | Antigen | Allergen | 0.147447 | Positive |
| HLA-DRB1*03:01 | 15 | EVTISNDAKPVAPRS | 0.9791 | 0.04 | 0.3013 | Non-Antigen | Non-Allergen | 0.023992227 | Positive |
| HLA-DRB1*03:01 | 15 | VEVTISNDAKPVAPR | 0.9766 | 0.05 | 0.4458 | Antigen | Non-Allergen | 0.085607353 | Positive |
| HLA-DRB1*04:05 | 15 | QARSNFSALQSQPDA | 0.9697 | 0.06 | 0.6547 | Antigen | Non-Allergen | -0.062136668 | Negative |
| HLA-DRB1*03:01 | 15 | RVEVTISNDAKPVAP | 0.9581 | 0.07 | 0.3591 | Non-Antigen | Non-Allergen | 1 | Positive |
| HLA-DRB1*03:01 | 15 | VTISNDAKPVAPRSS | 0.9565 | 0.07 | 0.2209 | Non-Antigen | Non-Allergen | 1 | Negative |
| HLA-DRB1*04:05 | 15 | SNFSALQSQPDATKV | 0.9515 | 0.12 | 0.5946 | Antigen | Allergen | 0.039074114 | Positive |
| HLA-DRB1*11:01 | 15 | SVRMALLSRGISPER | 0.9084 | 0.38 | 0.3969 | Non-Antigen | Non-Allergen | 2 | Positive |
| HLA-DQA1*01:02/DQB1*06:02 | 15 | VLRNAEAQLQNASAQ | 0.8975 | 0.01 | 0.7313 | Antigen | Non-Allergen | 0.53208248 | Positive |
| HLA-DRB1*04:01 | 15 | RSNFSALQSQPDATK | 0.8744 | 0.28 | 0.7127 | Antigen | Non-Allergen | -0.063505068 | Negative |
| HLA-DRB1*01:01 | 15 | EAQLQNASAQRAQAR | 0.8624 | 0.61 | 1.3141 | Antigen | Non-Allergen | 0.73876412 | Positive |
| HLA-DRB1*11:01 | 15 | DSVRMALLSRGISPE | 0.8617 | 0.6 | 0.3134 | Non-Antigen | Non-Allergen | 0.12958108 | Positive |
| HLA-DQA1*01:02/DQB1*06:02 | 15 | IVLRNAEAQLQNASA | 0.8601 | 0.03 | 0.7121 | Antigen | Non-Allergen | 0.21490656 | Positive |
| HLA-DRB1*15:01 | 15 | VDQLAYLTNQRIELA | 0.8573 | 0.31 | -0.0071 | Non-Antigen | Non-Allergen | 0.10582146 | Positive |
| HLA-DRB1*01:01 | 15 | VDQLAYLTNQRIELA | 0.856 | 0.63 | -0.0071 | Non-Antigen | Non-Allergen | 0.10582146 | Positive |
| HLA-DRB1*01:01 | 15 | RSNFSALQSQPDATK | 0.8527 | 0.65 | 0.7127 | Antigen | Non-Allergen | -0.063505068 | Negative |
| HLA-DRB1*04:05 | 15 | EQARSNFSALQSQPD | 0.8289 | 0.64 | 0.648 | Antigen | Allergen | -0.12916033 | Negative |
| HLA-DQA1*05:01/DQB1*03:01 | 15 | EAQLQNASAQRAQAR | 0.8171 | 0.31 | 1.3141 | Antigen | Non-Allergen | 0.73876412 | Positive |
| HLA-DRB1*11:01 | 15 | GDWLAKADKAYQDGE | 0.814 | 0.82 | 0.2932 | Non-Antigen | Non-Allergen | 0.46140581 | Positive |
| HLA-DRB1*15:01 | 15 | DVDQLAYLTNQRIEL | 0.8015 | 0.56 | 0.0913 | Non-Antigen | Allergen | -0.23500464 | Negative |
| HLA-DRB1*09:01 | 15 | NQRIELAKQTIVLRN | 0.8001 | 0.31 | 0.1822 | Non-Antigen | Non-Allergen | 0.084657544 | Positive |
| HLA-DRB1*04:01 | 15 | ARSNFSALQSQPDAT | 0.7962 | 0.77 | 0.7434 | Antigen | Allergen | 0.147447 | Positive |
| HLA-DQA1*01:02/DQB1*06:02 | 15 | TIVLRNAEAQLQNAS | 0.7961 | 0.1 | 0.5276 | Antigen | Non-Allergen | 0.2390642 | Positive |
| HLA-DRB1*12:01 | 15 | VDQLAYLTNQRIELA | 0.7844 | 0.22 | -0.0071 | Non-Antigen | Non-Allergen | 0.10582146 | Positive |
| HLA-DRB1*01:01 | 15 | DVDQLAYLTNQRIEL | 0.7772 | 1.2 | 0.0913 | Non-Antigen | Allergen | -0.23500464 | Negative |
| HLA-DQA1*01:02/DQB1*06:02 | 15 | EAQLQNASAQRAQAR | 0.7768 | 0.13 | 1.3141 | Antigen | Non-Allergen | 0.73876412 | Positive |
| HLA-DQA1*01:02/DQB1*06:02 | 15 | LRNAEAQLQNASAQR | 0.7767 | 0.13 | 1.0374 | Antigen | Allergen | 0.64440324 | Positive |
| HLA-DRB1*01:01 | 15 | AEAQLQNASAQRAQA | 0.7732 | 1.2 | 1.1419 | Antigen | Allergen | 0.78745985 | Positive |
| HLA-DQA1*05:01/DQB1*03:01 | 15 | AEAQLQNASAQRAQA | 0.7618 | 0.55 | 1.1419 | Antigen | Allergen | 0.78745985 | Positive |
| HLA-DRB1*09:01 | 15 | TNQRIELAKQTIVLR | 0.7553 | 0.45 | 0.3747 | Non-Antigen | Non-Allergen | 0.31264569 | Positive |
| HLA-DRB1*03:01 | 15 | TISNDAKPVAPRSSV | 0.7522 | 1.1 | 0.19 | Non-Antigen | Non-Allergen | 1 | Positive |
