# Supplementary material for: Rational design of an epitope-centric vaccine against Pseudomonas aeruginosa using pangenomic insights and immunoinformatics approach
Source: Front Immunol. 2025 Sep 1;16:1617251. doi: 10.3389/fimmu.2025.1617251 (PMC12434008; doi:10.3389/fimmu.2025.1617251)
Supplement: Supplementary file 5 [file Table5.docx]

**Rational Design of an Epitope-Centric Vaccine Against *Pseudomonas aeruginosa* using Pangenomic Insights and Immunoinformatics Approach**

**Supplementary Table 5:** Cytokine induction profiles of selected epitopes

| Peptide ID | Peptide Sequence | IL4 | IL6 | IL10 | IL13 |
| --- | --- | --- | --- | --- | --- |
| 1 | VEVTISNDAKPVAPR | IL4 Inducer (1.07) | Non IL6 Inducer (0.01) | IL10 Inducer (0.563) | Non IL13 Inducer (0.03) |
| 2 | VLRNAEAQLQNASAQ | Non IL4 Inducer (-0.11) | IL6 Inducer (0.09) | IL10 Inducer (0.418) | IL13 Inducer (0.06) |
| 3 | EAQLQNASAQRAQAR | IL4 Inducer (0.38) | IL6 Inducer (0.11) | IL10 Inducer (0.492) | IL13 Inducer (0.11) |
| 4 | IVLRNAEAQLQNASA | Non IL4 Inducer (-0.12) | IL6 Inducer (0.08) | IL10 Inducer (0.427) | Non IL13 Inducer (0.05) |
| 5 | TIVLRNAEAQLQNAS | Non IL4 Inducer (-0.18) | Non IL6 Inducer (0.05) | IL10 Inducer (0.445) | IL13 Inducer (0.26) |
