# Supplementary material for: Rational design of an epitope-centric vaccine against Pseudomonas aeruginosa using pangenomic insights and immunoinformatics approach
Source: Front Immunol. 2025 Sep 1;16:1617251. doi: 10.3389/fimmu.2025.1617251 (PMC12434008; doi:10.3389/fimmu.2025.1617251)
Supplement: Supplementary file 6 [file Table6.docx]

**Rational Design of an Epitope-Centric Vaccine Against *Pseudomonas aeruginosa* using Pangenomic Insights and Immunoinformatics Approach**

**Supplementary Table 6:** Population coverage calculation results

| population/area | Class I |  |  | Class II |  |  | Class combined | |  |
| --- | --- | --- | --- | --- | --- | --- | --- | --- | --- |
|  | coverage^a^ | average_hit^b^ | pc90^c^ | coverage^a^ | average_hit^b^ | pc90^c^ | coverage^a^ | average_hit^b^ | pc90^c^ |
| Kenya | 44.73% | 0.55 | 0.18 | 97.44% | 4.43 | 1.58 | 98.58% | 4.98 | 2.04 |
| United States | 51.84% | 0.71 | 0.21 | 96.54% | 3.22 | 1.36 | 98.33% | 3.93 | 1.82 |
| Zimbabwe | 40.39% | 0.49 | 0.17 | 97.15% | 5.19 | 1.78 | 98.30% | 5.68 | 2.22 |
| Germany | 59.30% | 0.82 | 0.25 | 95.73% | 4.23 | 1.4 | 98.26% | 5.06 | 2.06 |
| France | 63.27% | 0.92 | 0.27 | 94.65% | 3.86 | 1.28 | 98.04% | 4.78 | 1.97 |
| Brazil | 43.51% | 0.56 | 0.18 | 96.34% | 2.44 | 1.3 | 97.93% | 3 | 1.6 |
| Argentina | 59.14% | 0.67 | 0.24 | 94.78% | 2.71 | 1.2 | 97.87% | 3.37 | 1.63 |
| Russia | 55.50% | 0.81 | 0.22 | 93.82% | 3.33 | 1.19 | 97.25% | 4.14 | 1.65 |
| England | 62.55% | 0.84 | 0.27 | 92.05% | 3.66 | 1.11 | 97.02% | 4.5 | 1.65 |
| West Indies | 54.95% | 0.77 | 0.22 | 93.28% | 3.79 | 1.18 | 96.97% | 4.56 | 1.65 |
| Italy | 64.64% | 0.94 | 0.28 | 87.74% | 2.19 | 0.82 | 95.67% | 3.12 | 1.33 |
| Canada | 0.00% | 0 | 0 | 95.58% | 1.77 | 1.19 | 95.58% | 1.77 | 1.19 |
| Taiwan | 58.69% | 0.77 | 0.24 | 87.28% | 2.36 | 0.79 | 94.74% | 3.14 | 1.26 |
| Iran | 49.10% | 0.69 | 0.2 | 86.29% | 2.52 | 0.73 | 93.02% | 3.21 | 1.15 |
| Turkey | 0.00% | 0 | 0 | 92.66% | 3.1 | 1.11 | 92.66% | 3.1 | 1.11 |
| North Africa | 46.50% | 0.59 | 0.19 | 85.83% | 2.96 | 0.71 | 92.42% | 3.55 | 1.13 |
| Israel | 44.43% | 0.62 | 0.18 | 86.33% | 2.35 | 0.73 | 92.40% | 2.97 | 1.11 |
| Mongolia | 36.25% | 0.41 | 0.16 | 87.66% | 2.2 | 0.81 | 92.13% | 2.61 | 1.09 |
| Chile | 36.69% | 0.48 | 0.16 | 87.37% | 2.15 | 0.79 | 92.00% | 2.63 | 1.09 |
| Spain | 34.51% | 0.41 | 0.15 | 87.00% | 3.2 | 0.77 | 91.49% | 3.61 | 1.08 |
| Central America | 2.77% | 0.03 | 0.1 | 91.03% | 1.63 | 1.03 | 91.28% | 1.66 | 1.03 |
| Portugal | 47.07% | 0.63 | 0.19 | 81.68% | 2.38 | 0.55 | 90.30% | 3.01 | 1.01 |
| China | 33.76% | 0.43 | 0.15 | 84.61% | 2.68 | 0.65 | 89.81% | 3.11 | 0.98 |
| Japan | 46.31% | 0.69 | 0.19 | 76.91% | 2.34 | 0.43 | 87.60% | 3.03 | 0.81 |
| East Asia | 45.40% | 0.67 | 0.18 | 77.25% | 2.32 | 0.44 | 87.58% | 2.99 | 0.81 |
| World | 31.20% | 0.34 | 0.15 | 81.61% | 2.49 | 0.54 | 87.35% | 2.83 | 0.79 |
| Scotland | 20.85% | 0.22 | 0.13 | 82.96% | 2.5 | 0.59 | 86.51% | 2.72 | 0.74 |
| Thailand | 37.14% | 0.44 | 0.16 | 77.80% | 2.35 | 0.45 | 86.05% | 2.8 | 0.72 |
| Korea; South | 46.25% | 0.68 | 0.19 | 73.60% | 2.27 | 0.38 | 85.81% | 2.94 | 0.7 |
| India | 43.18% | 0.55 | 0.18 | 74.58% | 1.96 | 0.39 | 85.56% | 2.51 | 0.69 |
| Australia | 38.45% | 0.46 | 0.16 | 75.20% | 1.86 | 0.4 | 84.74% | 2.32 | 0.66 |
| Netherlands | 0.00% | 0 | 0 | 84.33% | 2.89 | 0.64 | 84.33% | 2.89 | 0.64 |
| Singapore | 40.26% | 0.5 | 0.17 | 71.87% | 1.21 | 0.36 | 83.19% | 1.71 | 0.6 |
| Indonesia | 27.34% | 0.32 | 0.14 | 71.86% | 1.8 | 0.36 | 79.56% | 2.12 | 0.49 |
| Sudan | 44.96% | 0.57 | 0.18 | 58.47% | 0.71 | 0.24 | 77.14% | 1.28 | 0.44 |
| Saudi Arabia | 54.08% | 0.72 | 0.22 | 49.69% | 0.97 | 0.2 | 76.90% | 1.69 | 0.43 |
| New Zealand | 0.00% | 0 | 0 | 72.00% | 1.31 | 0.36 | 72.00% | 1.31 | 0.36 |
| Malaysia | 21.95% | 0.23 | 0.13 | 59.42% | 0.89 | 0.25 | 68.32% | 1.11 | 0.32 |
| Pakistan | 43.77% | 0.63 | 0.18 | 33.37% | 0.69 | 0.15 | 62.53% | 1.31 | 0.27 |
| South Africa | 45.47% | 0.56 | 0.18 | 27.07% | 0.27 | 0.14 | 60.23% | 0.84 | 0.25 |
| American Samoa | 56.40% | 0.75 | 0.23 | 0.00% | 0 | 0 | 56.40% | 0.75 | 0.23 |
| Hong Kong | 0.00% | 0 | 0 | 56.64% | 0.75 | 0.23 | 56.64% | 0.6 | 0.19 |
| United Kingdom | 0.00% | 0 | 0 | 56.38% | 0.38 | 0.16 | 56.38% | 0.38 | 0.16 |
| Average | 37.03 | 0.48 | 0.16 | 69.74 | 2.05 | 0.65 | 79.7 | 2.54 | 0.92 |
| Standard deviation | 19.39 | 0.27 | 0.07 | 30.19 | 1.29 | 0.46 | 22.33 | 1.42 | 0.59 |

^a^ projected population coverage
^b^ average number of epitope hits / HLA combinations recognized by the population
^c^ minimum number of epitope hits / HLA combinations recognized by 90% of the population
