# Supplementary material for: Rational design of an epitope-centric vaccine against Pseudomonas aeruginosa using pangenomic insights and immunoinformatics approach
Source: Front Immunol. 2025 Sep 1;16:1617251. doi: 10.3389/fimmu.2025.1617251 (PMC12434008; doi:10.3389/fimmu.2025.1617251)
Supplement: Supplementary file 7 [file Table7.docx]

**Rational Design of an Epitope-Centric Vaccine Against *Pseudomonas aeruginosa* using Pangenomic Insights and Immunoinformatics Approach**


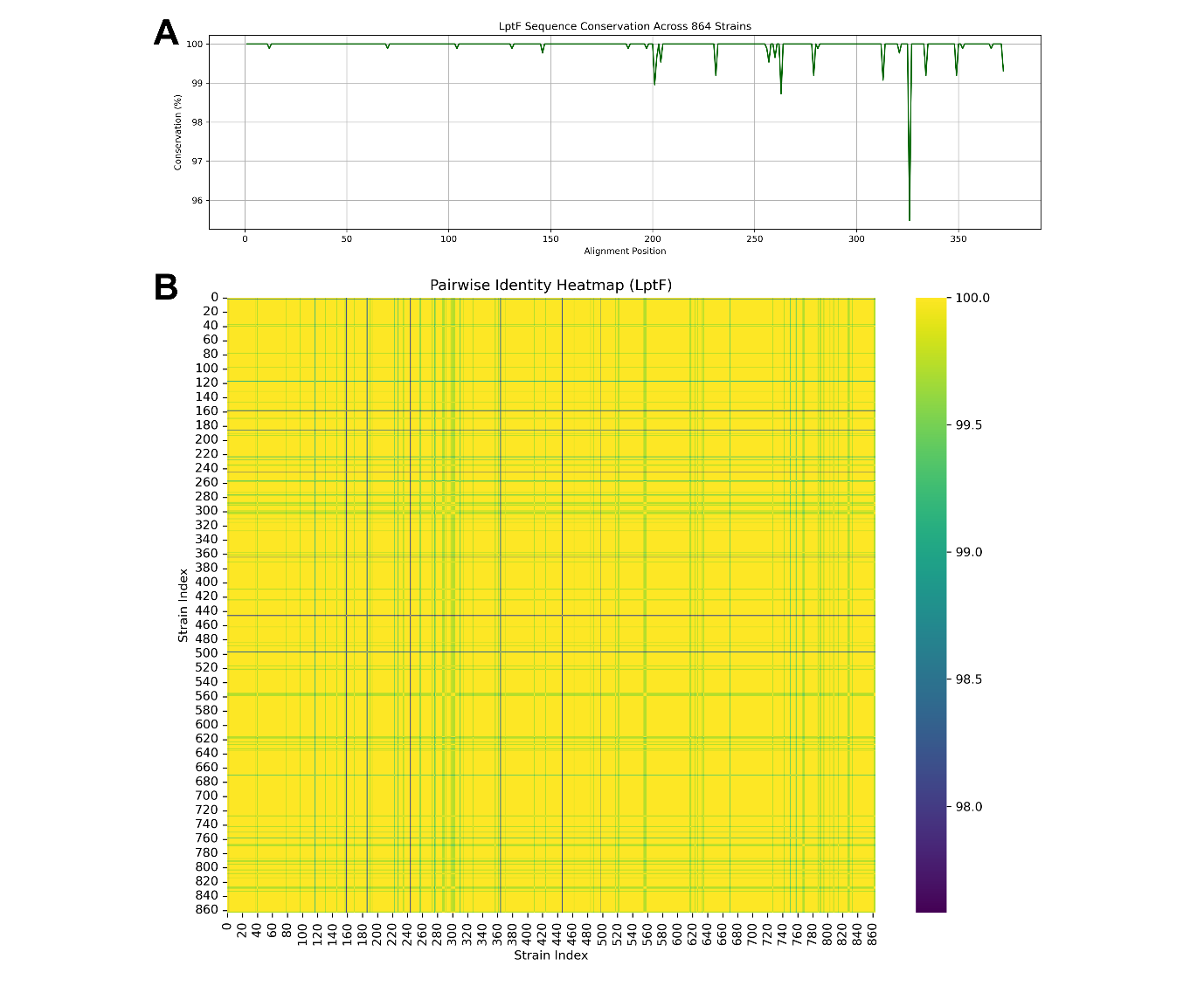


**Supplementary Figure 1:** Conservation analysis of LptF protein across *P. aeruginosa* strains. **(A)** Sequence alignment reveals that more than 98% of LptF amino acid residues are conserved across all strains, indicating strong conservation. **(B)** Pairwise identity heatmap reveals more than 99% similarity among strains, further supporting LptF as a stable and widely conserved vaccine target
