# Supplementary material for: Rational design of an epitope-centric vaccine against Pseudomonas aeruginosa using pangenomic insights and immunoinformatics approach
Source: Front Immunol. 2025 Sep 1;16:1617251. doi: 10.3389/fimmu.2025.1617251 (PMC12434008; doi:10.3389/fimmu.2025.1617251)
Supplement: Supplementary file 10 [file Table10.docx]

**Rational Design of an Epitope-Centric Vaccine Against *Pseudomonas aeruginosa* using Pangenomic Insights and Immunoinformatics Approach**


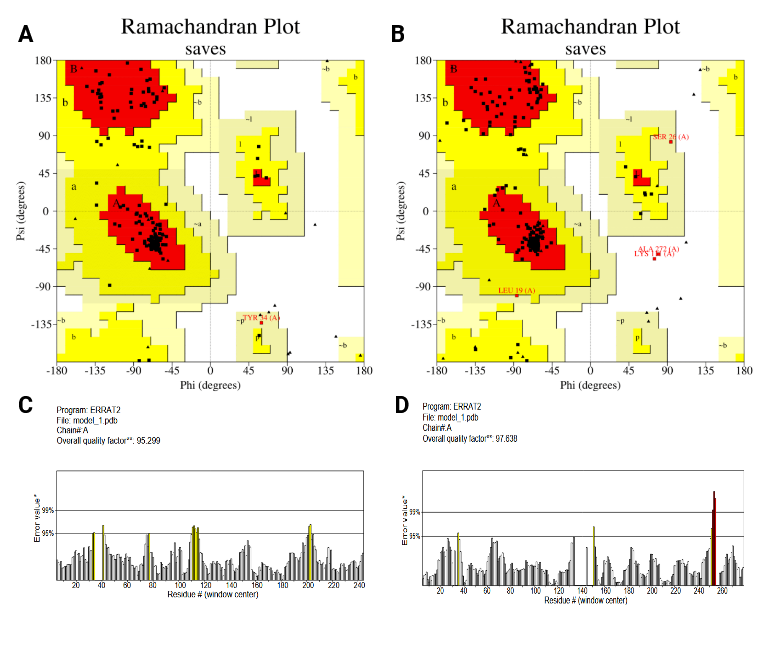


**Supplementary Figure 4:** **Ramachandran plot analysis for the finalized vaccine constructs. (A)** POA_V_RS09, showing 93.6% of residues in favored regions, indicating a highly reliable model. **(B)** POA_V_BDEF, with 90.5% of residues in the favored areas, demonstrating robust structural validation. **Visualization of the overall quality factor plot (ERRAT).** **(C)** POA_V_RS09, **(D)** POA_V_BDEF.
