# Supplementary material for: Rational design of an epitope-centric vaccine against Pseudomonas aeruginosa using pangenomic insights and immunoinformatics approach
Source: Front Immunol. 2025 Sep 1;16:1617251. doi: 10.3389/fimmu.2025.1617251 (PMC12434008; doi:10.3389/fimmu.2025.1617251)
Supplement: Supplementary file 11 [file Table11.docx]

**Rational Design of an Epitope-Centric Vaccine Against *Pseudomonas aeruginosa* using Pangenomic Insights and Immunoinformatics Approach**


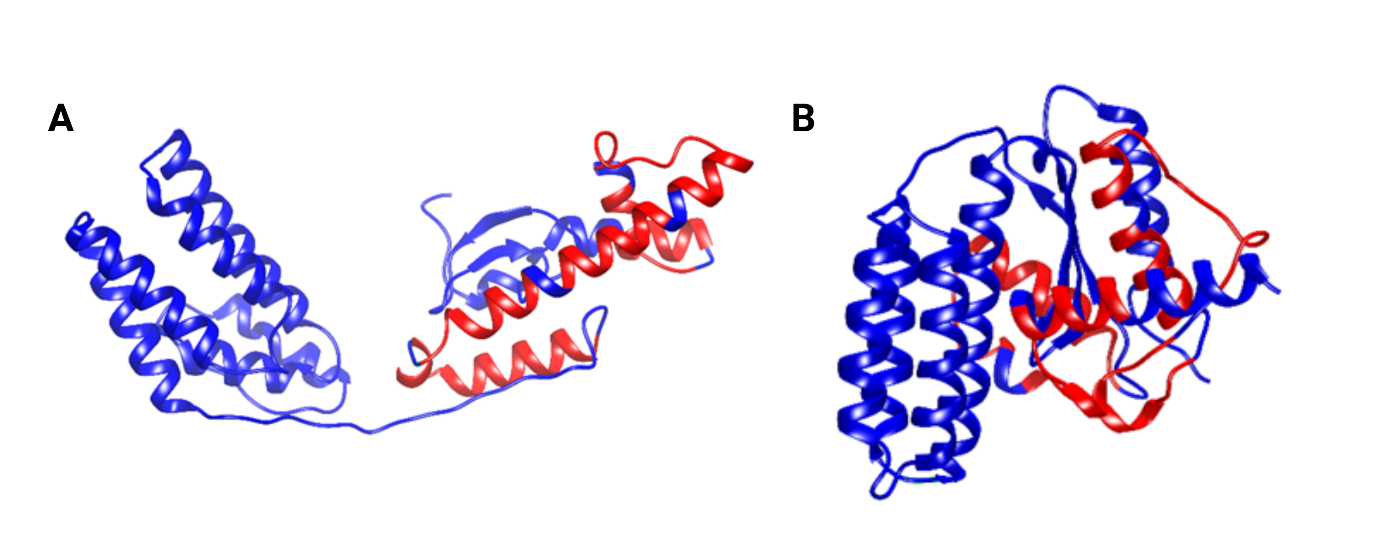


**Supplementary Figure 5:** **The 3D Structure of the POA_V Vaccines, where Red colour depicts the B-Cell epitope.** (A) Displays the POA_V_BDEF. (B) Displays POA_V_RS09.
