# Supplementary material for: Rational design of an epitope-centric vaccine against Pseudomonas aeruginosa using pangenomic insights and immunoinformatics approach
Source: Front Immunol. 2025 Sep 1;16:1617251. doi: 10.3389/fimmu.2025.1617251 (PMC12434008; doi:10.3389/fimmu.2025.1617251)
Supplement: Supplementary file 12 [file Table12.docx]

**Rational Design of an Epitope-Centric Vaccine Against *Pseudomonas aeruginosa* using Pangenomic Insights and Immunoinformatics Approach**


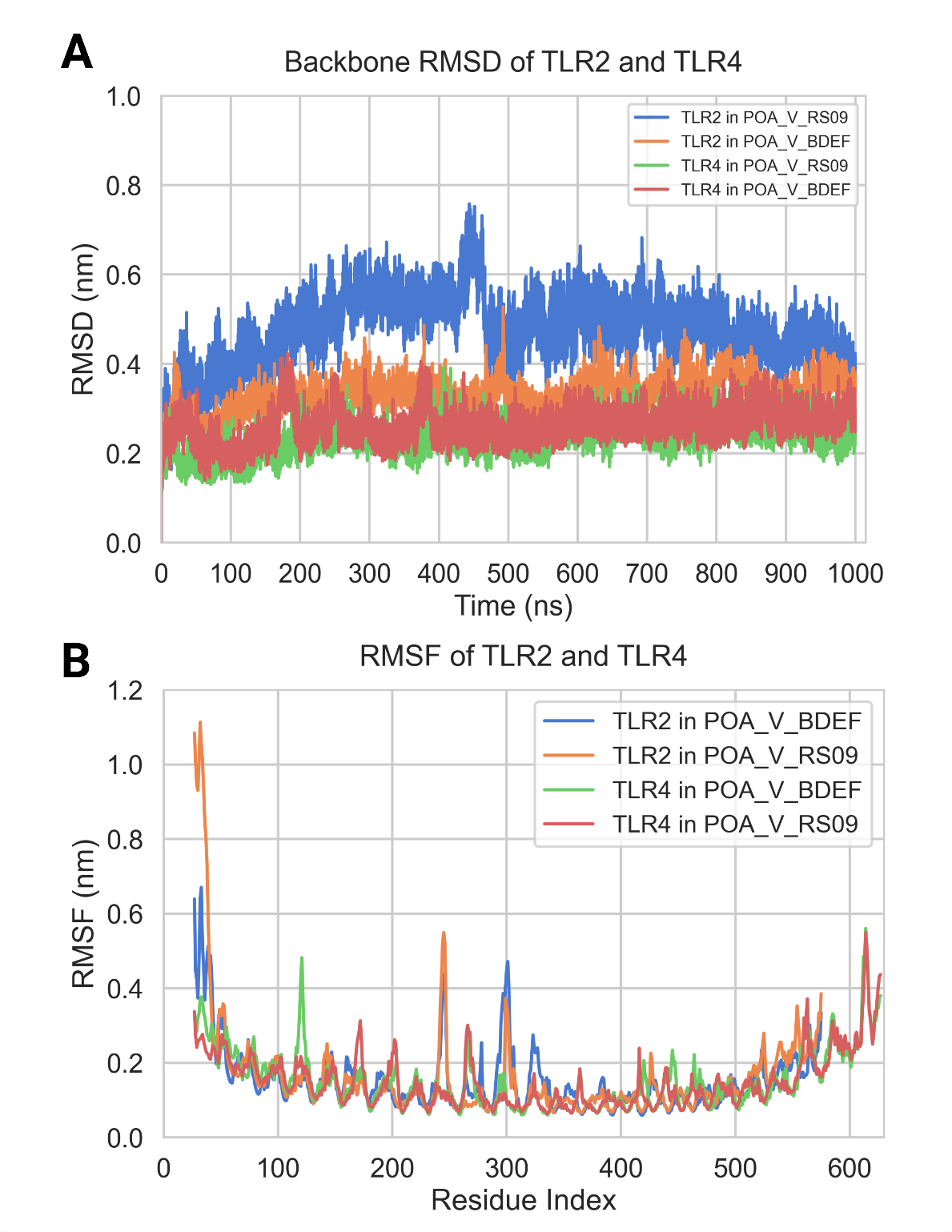


**Supplementary Figure 6:** **Structural Stability Assessment for Standalone TLR2 and TLR4 complexes. (A)** Backbone RMSD. **(B)** Calpha RMSF.
