# Supplementary material for: Rational design of an epitope-centric vaccine against Pseudomonas aeruginosa using pangenomic insights and immunoinformatics approach
Source: Front Immunol. 2025 Sep 1;16:1617251. doi: 10.3389/fimmu.2025.1617251 (PMC12434008; doi:10.3389/fimmu.2025.1617251)
Supplement: Supplementary file 13 [file Table13.docx]

**Rational Design of an Epitope-Centric Vaccine Against *Pseudomonas aeruginosa* using Pangenomic Insights and Immunoinformatics Approach**


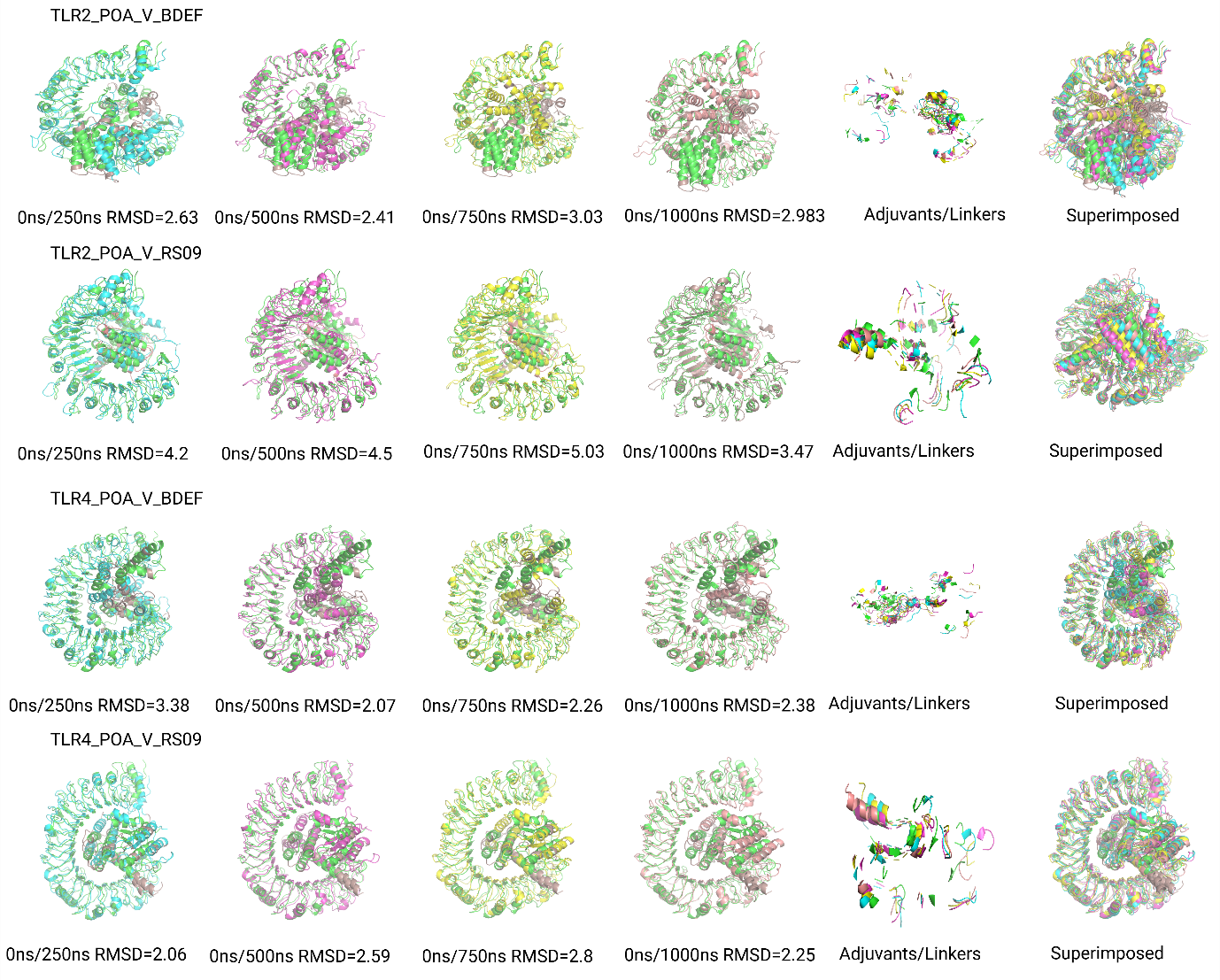


**Supplementary Figure 7:** Superimposed temporal snapshots of vaccine & receptor complexes at 0ns, 250ns, 500ns, 750ns, and 1000 ns. Adjuvant and linker regions were included in the overlays to evaluate structural consistency throughout the simulation.
