# Supplementary material for: Rational design of an epitope-centric vaccine against Pseudomonas aeruginosa using pangenomic insights and immunoinformatics approach
Source: Front Immunol. 2025 Sep 1;16:1617251. doi: 10.3389/fimmu.2025.1617251 (PMC12434008; doi:10.3389/fimmu.2025.1617251)
Supplement: Supplementary file 14 [file Table14.docx]

**Rational Design of an Epitope-Centric Vaccine Against *Pseudomonas aeruginosa* using Pangenomic Insights and Immunoinformatics Approach**


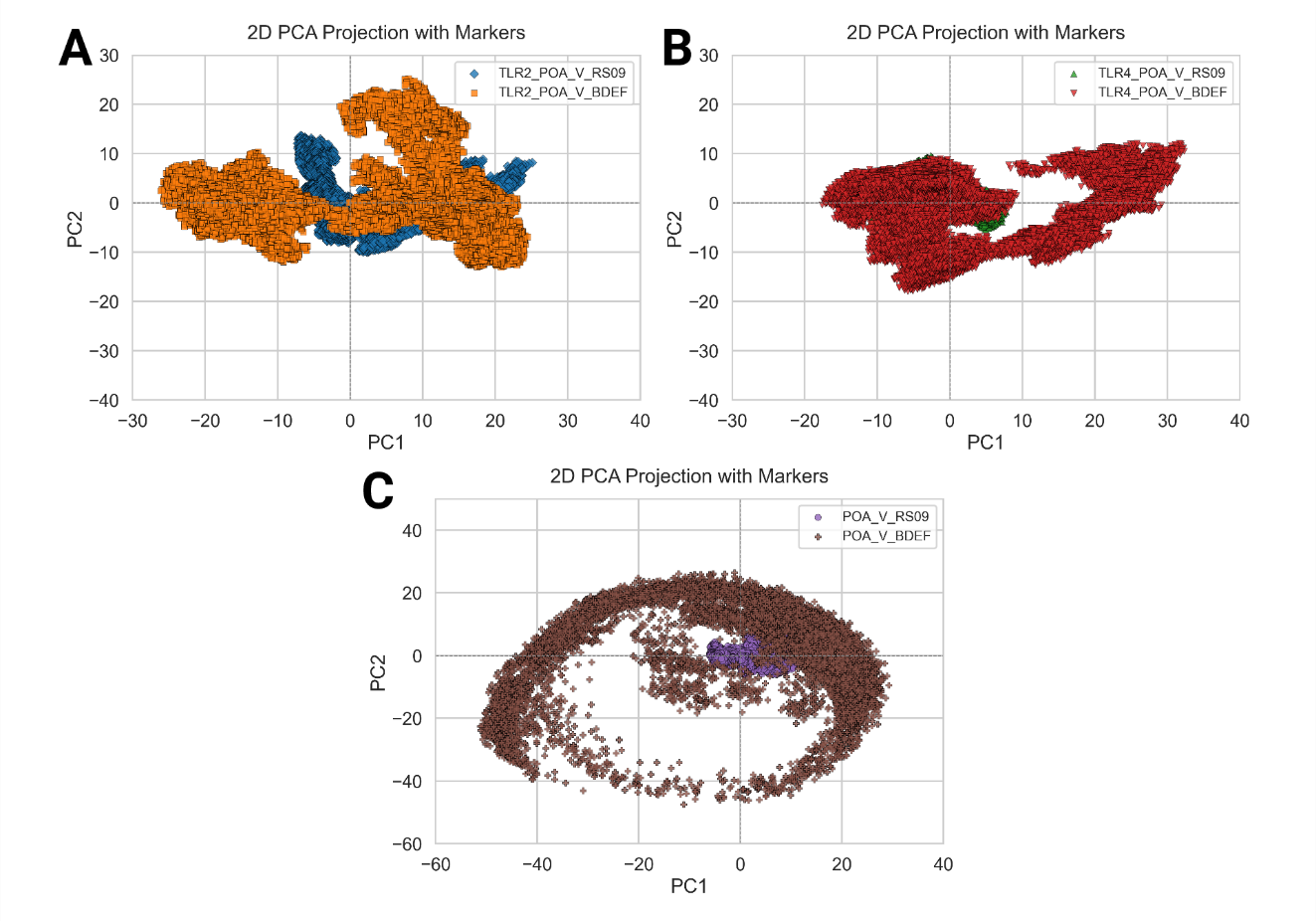


**Supplementary Figure 8:** PCA was conducted on the POA_V_RS09 and POA_V_BDEF constructs, extracted from their respective TLR2 and TLR4 complexes, to evaluate their conformational behavior throughout the 1000 ns molecular dynamics simulations. The plot shows that the POA_V_RS09 construct exhibits greater structural stability than POA_V_BDEF, as indicated by its compact clustering in the simulation trajectory.
