# Supplementary material for: Rational design of an epitope-centric vaccine against Pseudomonas aeruginosa using pangenomic insights and immunoinformatics approach
Source: Front Immunol. 2025 Sep 1;16:1617251. doi: 10.3389/fimmu.2025.1617251 (PMC12434008; doi:10.3389/fimmu.2025.1617251)
Supplement: Supplementary file 15 [file Table15.docx]

**Rational Design of an Epitope-Centric Vaccine Against *Pseudomonas aeruginosa* using Pangenomic Insights and Immunoinformatics Approach**


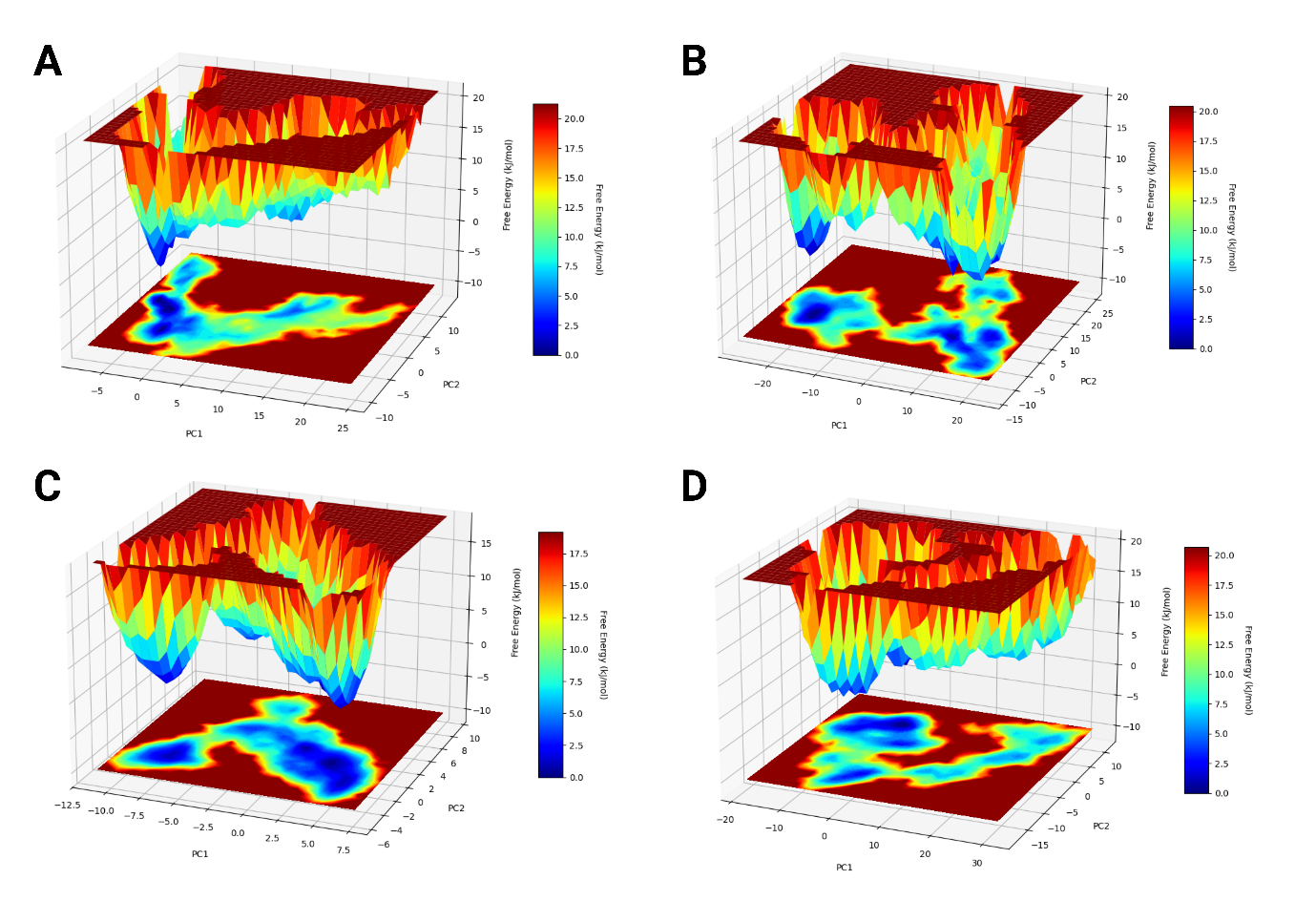


**Supplementary Figure 9**: Results of the free energy landscape (FEL) analysis. **(A-B)**TLR2_POA_V_RS09 and TLR2_POA_V_BDEF complexes. **(C-D)** TLR4_POA_V_RS09 and TLR4_POA_V_BDEF complexes.
